# Beyond the Threshold: A Study of Chalcogenophene-Based Two-Photon Initiators

**DOI:** 10.1021/acs.chemmater.1c04002

**Published:** 2022-03-22

**Authors:** Markus Lunzer, Joseph S. Beckwith, Franziska Chalupa-Gantner, Arnulf Rosspeintner, Giuseppe Licari, Wolfgang Steiger, Christian Hametner, Robert Liska, Johannes Fröhlich, Eric Vauthey, Aleksandr Ovsianikov, Brigitte Holzer

**Affiliations:** † Institute of Applied Synthetic Chemistry, TU Wien, Getreidemarkt 9/163, 1060 Vienna, Austria; ‡ Institute of Materials Science and Technology, TU Wien, Getreidemarkt 9/308, 1060 Vienna, Austria; § UpNano GmbH, Modecenterstraße 22/D36, 1030 Vienna, Austria; ∥ Department of Physical Chemistry, University of Geneva, 30 Quai Ernest-Ansermet, 1211 Geneva, Switzerland

## Abstract

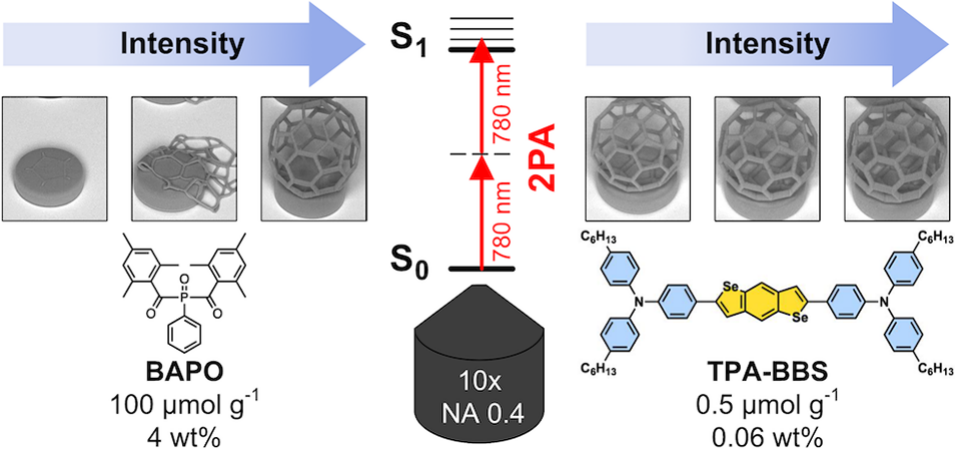

A series of nine soluble, symmetric chalcogenophenes bearing hexyl-substituted triphenylamines, indolocarbazoles, or phenylcarbazoles was designed and synthesized as potential two-photon absorption (2PA) initiators. A detailed photophysical analysis of these molecules revealed good 2PA properties of the series and, in particular, a strong influence of selenium on the 2PA cross sections, rendering these materials especially promising new 2PA photoinitiators. Structuring and threshold tests proved the efficiency and broad spectral versatility of two selenium-containing lead compounds as well as their applicability in an acrylate resin formulation. A comparison with commercial photoinitiators **Irg369** and **BAPO** as well as sensitizer **ITX** showed that the newly designed selenium-based materials **TPA-S** and **TPA-BBS** outperform these traditional initiators by far both in terms of reactivity and dose. Moreover, by increasing the ultralow concentration of **TPA-BBS**, a further reduction of the polymerization threshold can be achieved, revealing the great potential of this series for application in two-photon polymerization (2PP) systems where only low laser power is available.

## Introduction

Additive manufacturing is a rapidly growing, versatile fabrication technology that allows the digital design and production of 3D objects.^1−3^ Among the various advanced 3D printing technologies, two-photon lithography holds a special role as it permits the production of highly detailed 3D objects, the properties of which may be tailored to the application.^4,5^ Applications include but are not limited to advanced micro-optics,^6^ microfluidics,^7,8^ metamaterials,^9−11^ and micromachines^12−14^ as well as utilizations in the biological and biomedical field.^15−18^

Although two-photon polymerization (2PP) was first demonstrated more than 20 years ago,^19,20^ it remained an academic curiosity for more than a decade. Recently, however, reliable commercial 3D printing systems have become available.^21^ Despite this, the full potential of this technology, especially in an industrial context, has not been fully explored yet. As two-photon polymerization offers exceptionally high resolution and accuracy, typical print durations are accordingly long and depending on the part size, and chosen printing parameters can take from several hours to days. Hence, only low production volumes can be realized.^22^ In order to scale up the printing speed and production capabilities of two-photon lithography, several strategies have been presented, including the faster scanning of single voxels^23,24^ as well as parallel scanning of multiple foci created by microlens arrays,^25,26^ diffractive optical elements,^27^ or via spatial light modulators^28−30^ and by faster scanning thereof.^31^ Another interesting approach uses spatiotemporal focusing of a fs-pulsed laser^32^ to implement a projection-based layer-by-layer parallelization.^33^

Regardless of the chosen technology, increasing the printing speed requires the delivery of sufficient energy to polymerize an individual volume element within the short period of irradiation.^24^ On the 3D printing system side, the applied laser power can be increased along with the printing speed up to several watts, but this is limited by the available laser sources and difficulties that come with the handling of such immense laser powers. Apart from challenges in instrumental design and engineering, the laser intensity can only be increased to a certain amount until microexplosions occur in the printing material due to parasitic N-photon absorption of the monomer resin (this is known as the upper polymerization limit).^34,35^ Consequently, more sensitive two-photon active resins are necessary for the improvement of 3D printing technology.^36^

While the chemical curing rate of a resin can be optimized by combining different monomer components, the two-photon reactivity of a particular resin mixture strongly depends on the efficiency of the two-photon active initiator species. Two-photon absorption (2PA) is a nonlinear process with a wavelength-dependent molecular 2PA cross section (σ^(2)^) reported in the unit Goeppert-Mayer (1 GM = 10^–50^ cm^4^ s photon^–1^ molecule^–1^).^37^ However, a high σ^(2)^ may not necessarily result in a high two-photon polymerization efficiency at a certain wavelength as different relaxation pathways may ensue that do not contribute to the initiation process.

Commercial two-photon 3D-printing systems utilize either green laser light (∼520 nm, Microlight3D,^38^ Multiphoton Optics,^39^ Femtika^40^) or near infra-red (NIR) laser light (∼780 nm, Nanoscribe,^41^ UpNano^42^) meaning that photoinitiators with high efficiency at these wavelengths are indispensable. Due to the phenomenon of near-resonance enhancement of σ^(2)^, various two-photon active substance classes are known to exhibit exceptionally high two-photon absorption and good initiation properties in the green region of the spectrum (∼520 nm).^38,43^ In contrast, fine-tuning of the molecular design is still required to achieve high two-photon reactivity in the NIR spectral region (∼780 nm).^37,44^

Recently, we have reported on the synthesis and photophysical properties of a series of cap–linker–cap systems based on substituted thiophenes bearing various triphenylamines with two-photon absorption maxima in the NIR and efficient two-photon initiation properties (verified by polymerization tests).^45−47^ In this work, we expand on this previous design strategy to further increase the two-photon absorption and, thus, the efficiency of 2PA initiators based on quadrupolar cap–linker–cap type molecules.

**Figure 1:**
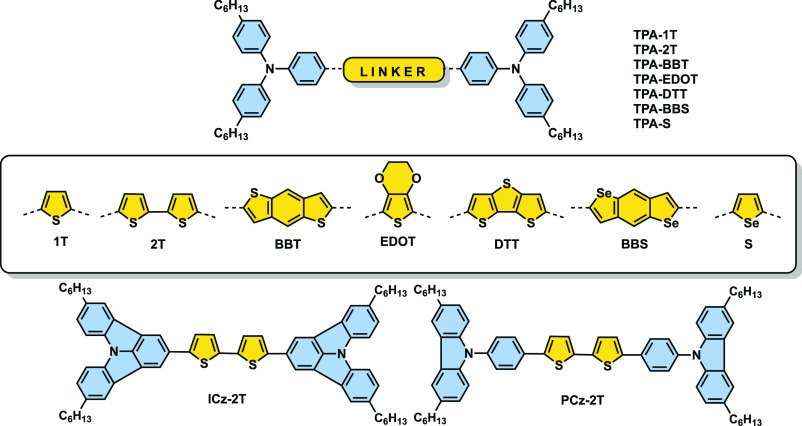
Target compounds based on triphenylamine (**TPA**), indolocarbazole (**ICz**), and phenylcarbazole (**PCz**).

Here, we focus on the design and application of two-photon active initiators exhibiting high polymerization efficiency in the NIR region. The following strategies are applied to increase the two-photon activity (Figure 1): (i) planarization of the triphenylamine (**TPA**) cap by introduction of indolocarbazole (**ICz**) or phenylcarbazole (**PCz**) moieties, (ii) elongation and planarization of the linker by integrating bithiophene (**2T**), benzo[1,2-*b*:4,5-*b*]dichalcogenophenes (**BBT** and **BBS**) or dithieno[3,2-*b*:2′,3′-*d*]thiophene (**DTT**), and (iii) enhancing the electron density of the linker using more electron-donating selenophene (**S**) or 2,3-dihydrothieno[3,4-*b*]-1,4-dioxine (**EDOT**) (Figure 1). To facilitate sufficient solubility in photopolymer resin formulations, all target compounds were designed bearing peripheral hexyl substituents on the triarylamine building blocks.

Based on a detailed photophysical study and TD-DFT calculations, we identify the most promising compound and then demonstrate its efficiency in an acrylic resin formulation. In contrast to commercially available UV-photoinitiators 2-benzyl-2-dimethylamino-1-(4-morpholinophenyl)-butanone-1 (**Irg369**)^48,49^ and phenylbis(2,4,6-trimethylbenzoyl)phosphine oxide (**BAPO**)^50^ as well as 2-isopropylthiaxanthone (**ITX**)^51^ with coinitiator methyldiethanolamine (**MDEA**), the new photoinitiators require lower laser powers for the fabrication of stable and accurate structures when excited by NIR-laser light (780 nm) and much lower necessary concentrations for use.

## Results and Discussion

### Synthesis

The cap scaffolds **TPA**, **ICz**, and **PCz** were connected to the chalcogenophene-based π-linkers toward symmetrical α,ω-bis(triarylamines) by cross-coupling reactions based on Suzuki or Stille coupling as well as CH activation. The synthesis of boronic ester-substituted **TPA**, **ICz**, and **PCz** was realized according to our recently published protocol.^52^ A Suzuki cross-coupling procedure developed in the same study^52^ was used for connecting thiophene, bithiophene, and dithieno[3,2-*b*:2′,3′-*d*]thiophene bromides with the obtained boronate, yielding **TPA-1T**, **TPA-2T**, and **TPA-DTT**. The same protocol was successfully applied for the combination of boronic ester-substituted **ICz** and **PCz** with bithiophene bromide toward target compounds **ICz-2T** and **PCz-2T**. The syntheses of **TPA-BBT**, **TPA-S**, and **TPA-BBS** were achieved by Stille cross coupling using benzo[1,2-*b*:4,5-*b*]dichalcogenophenes or selenophene distannane and bromide-substituted **TPA**. The introduction of the EDOT linker in **TPA-EDOT** was realized by CH activation, applying bromide-substituted **TPA** adapting a procedure by Liu et al*.*^53^ Detailed synthetic procedures are given in the Supporting Information. The obtained solid-to-viscous materials appear yellow to red in color and are soluble in common organic solvents. All target compounds were characterized by ^1^H/^13^C NMR spectroscopy and HR-MS analysis (Supporting Information). The data are consistent with the proposed structural formulations.

### One-Photon Steady-State and Transient Spectroscopy

**Figure 2:**
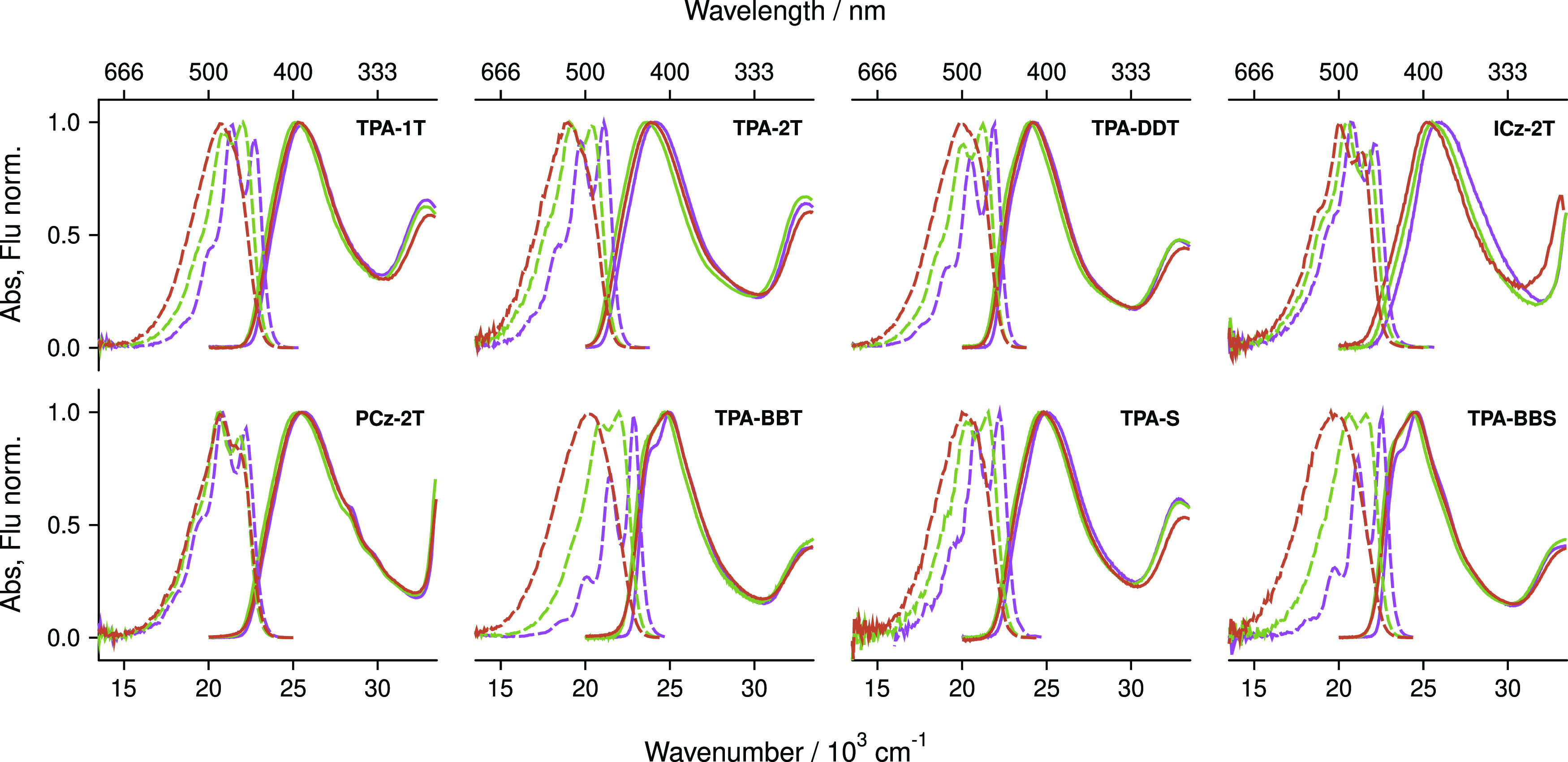
Absorption (solid lines) and fluorescence (broken lines) spectra of all photoinitiators save **TPA-EDOT** (qualitatively similar to **TPA-1 T**; spectrum shown in Figure S51) in solvents of different polarity. Red = acetonitrile/benzonitile, green = THF, purple = hexane.

Figure 2 shows the absorption and emission spectra of eight target molecules in solvents of different polarity (**TPA-EDOT** is not shown as the spectral shape is identical to **TPA-1T**). All of them exhibit an intense (ε > 4 × 10^4^ M^–1^ cm^–1^) broad absorption band with the maximum ranging from 25,360 cm^–1^ (394 nm) (**ICz-2T**) to 23,981 cm^–1^ (417 nm) (**TPA-2T**). From the intensity of this band, its absence in the spectra of the single branch analogues, and the TD-DFT calculations, this band can be attributed to a transition involving the two DA branches.^54^ A second transition, assigned to the local π–π* transition of the donor, occurs at higher energies, ca. 1 × 10^4^ cm^–1^, with a lower ε. All compounds fluoresce, and the key photophysical parameters (molar absorption coefficients, 2PA cross-sections, and 1PA and 2PA maximum wavelengths) are compiled in Table 1. Further photophysical data are shown in the Supporting Information.

**Table 1:** Lowest-Energy 1PA Maximum Wavelength (λ_1PA_), Extinction Coefficient (ε), 2PA Maximum Wavelength (λ_2PA_), and the Corresponding Cross-Sectional Value (σ^(2)^) in THF^b^

	λ_1PA_ (nm)	10^–3^ ε (M^–1^ cm^–1^)	λ_2PA_ (nm)	σ^(2)^ (GM)^a^
**TPA-1T**	395	66	706	500 (50)
**TPA-2T**	422	56	714	430 (170)
**TPA-DTT**	417	68	712	540 (130)
**ICz-2T**	392	53	682	160 (40)
**PCz-2T**	393	55	690	660 (50)
**TPA-BBT**	410	75	714	680 (170)
**TPA-S**	406	42	714	1000 (150)
**TPA-BBS**	414	70	734	1800 (720)
**TPA-EDOT**	403	47	690	350 (25)

a 1 GM = 10^–50^ cm^4^ s photon^–1^ molecule^–1^, ± 20%.

b The σ^(2)^ value at 780 nm is given in parentheses. For a comprehensive list of all photophysical properties, see the Supporting Information.

As an additional remark, the spectra are not mirror-symmetric. This is due to the different curvatures of the S_0_ and S_1_ potentials along the torsional coordinate of the linker. The linker is very flexible in the S_0_ state and becomes more rigid in the S_1_ state because of conjugation.^55^ Such data, particularly the absorption spectra, tell us that these compounds will be influenced relatively little by their environment when it comes to collecting photons (see the absence of absorption solvatochromism in Figure 2), which ensures that they would be of use in a range of resin polarities. That these molecules have more polar excited states could be of use in the photoinitation reaction.

The fluorescence lifetimes were also measured using time-resolved fluorescence and analyzed using single exponential functions in all but three cases. Eighty-seven percent of the χ_ν_^2^ values of the fits were below 1.8, with the remainder below 3. For cases in which a biexponential fit was necessary, the shortest time constant (∼3 ps) was ascribed to solvation, and the longer time constant was taken as the fluorescence lifetime.

By combining the fluorescence lifetimes τ_fl_ and quantum yields ϕ_fl_, the radiative rate constants *k*_r_ can be obtained using
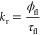
1and then, subsequently, one can obtain the nonradiative decay rate constant, *k*_nr_, using
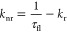
2

In order to decompose *k*_r_ into the intersystem crossing rate (*k*_ISC_) and internal conversion rate (*k*_IC_) constants, we have used transient absorption spectroscopy, which allows us to determine the intersystem crossing yield ϕ_ISC_ (see the SI). This allows painting a more quantitative picture of the reactive excited state populations and their contribution to photoinitiation.

**Figure 3:**
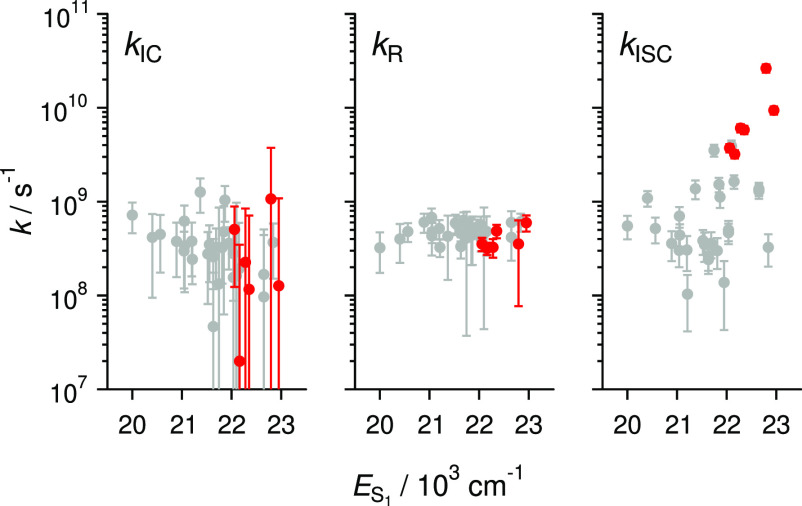
Internal conversion (*k*_IC_), radiative (*k*_r_), and intersystem crossing (*k*_ISC_) rate constants with respect to the singlet energy gap. The red points are selenium-based compounds. Singlet state energy was calculated as (ν_a_^max^ + ν_f_^cg^)/2.

The internal conversion rate constants, intersystem crossing rate constants, and radiative rate constants can be seen in Figure 3, and the ISC rate constants increase slightly as the singlet energy is increased. The increase of the intersystem crossing rate constants provides support for the notion that access from the S_1_ state to higher triplet states becomes more efficient as the solvent polarity is decreased as in a recently investigated series of photoinitiators.^56^ By contrast, on the same scale, the radiative and internal conversion rate constants remain virtually unchanged across the series. This clearly shows that the triplet yields are largely determined by the energy level change due to solvent. Additionally, a clear finding is highlighted by the red points, corresponding to selenium-containing compounds, that the use of selenium in the chromophores increases the triplet yields and enhances the intersystem crossing rate constants due to the heavy-atom effect. The integration of selenium in chromophores is an advantageous design strategy for applications where triplets are necessary for the photoinitation process. The necessity of triplets for the photoinitiation process is shown by virtue of a simple quenching experiment (Figure S53) in which the fluorescence lifetime shows no change with the addition of 1.2 M of trimethylolpropane triacrylate (TTA). This monomer is the same used in the structuring tests detailed below, and thus, the singlet cannot be relevant for the reactivity that leads to polymerization. As an aside, we do not have sufficient data to assign a mechanism for the photoinitiation process (and doing so goes beyond the scope of this work), but it must involve triplet excited states. Energy or electron transfer from the lowest-lying or higher-lying triplet states are possible, but an assignment of the mechanism requires further investigation.

### Two-Photon Absorption Spectroscopy

**Figure 4:**
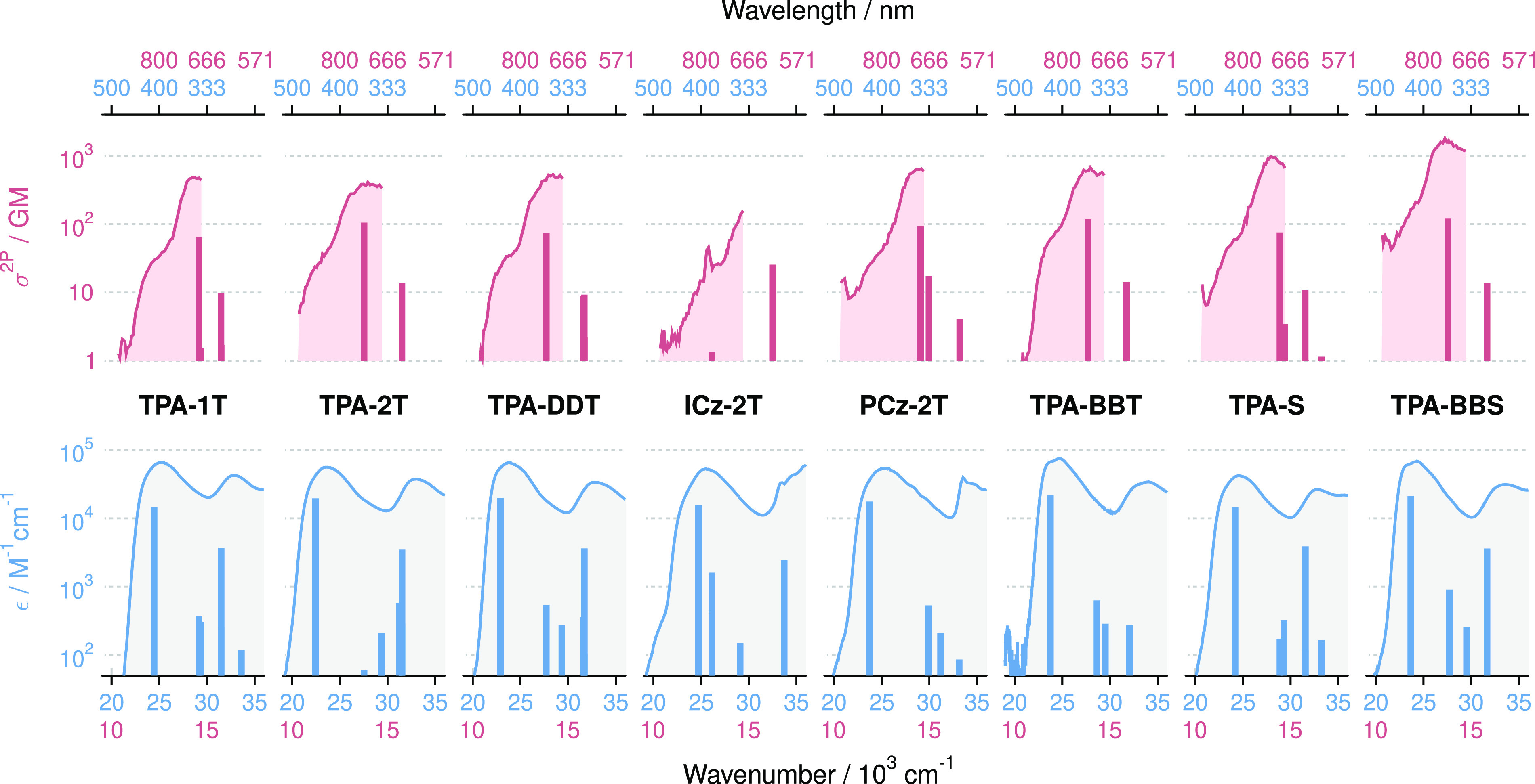
1PA (light blue) and 2PA (light red) spectra of **TPA-1T**, **TPA-2T**, **TPA-DTT**, **ICz-2 T**, **PCz-2 T**, **TPA-BBT**, **TPA-S**, and **TPA-BBS** in THF. Light blue sticks are calculated 1P transitions, and light red sticks are calculated 2P transitions (the calculated energies were downshifted by 4500 cm^–1^). A linear presentation of these spectra can be found in the Supporting Information.

The two-photon absorption cross sections of the photoinitiators were measured in THF by the two-photon excited fluorescence method,^57^ and the spectra are shown in Figure 4 along with a comparison to the one-photon absorption (1PA) spectra and calculated transitions strengths (sticks). The calculations agree reasonably well with the experiments, both in magnitude of strength and in position of transition. This allows us to observe that, for example, upon changing the cap from **TPA-2T** to **PCz-2T** to **ICz-2T**, the 2PA band shifts to higher energy, and the cross section slightly increases when going from **TPA-2T** to **PCz-2T**. This is ascribed to an increasing number of bonds between the phenyl rings, causing the cap to become more planar. This has two effects. On one hand, the 2PA cross section increases from **TPA-1T** to **TPA-BBS** (advantageous), while on the other hand, the 2PA peak is shifted more and more to higher energies and, thus, out of the technologically relevant window at 780 nm (adverse). **PCz-2T**, which is more blueshifted with respect to **TPA-2T** in the 1PA spectrum, is here sufficiently blueshifted that it is not possible to unequivocally measure the peak of the 2PA cross section using our setup.

The effect of varying the linker shows a few useful trends. Increasing the size and rigidity of the aromatic system of the linker to make the photoinitiator more quadrupolar appears to have a positive effect on the 2PA cross section (for example, upon changing the linker units from **TPA-1T** to **TPA-BBT**, the 2PA band both increases and shifts to lower energy). Going from sulfur- to selenium-containing photoinitiators also has a large effect on the maximum 2PA cross section σ_ECS_^(2)^ in going from **TPA-1T** to **TPA-S** or **TPA-BBT** to **TPA-BBS** as the 2PA cross section increases dramatically (factors of 2 and 2.6, respectively).

**Figure 5:**
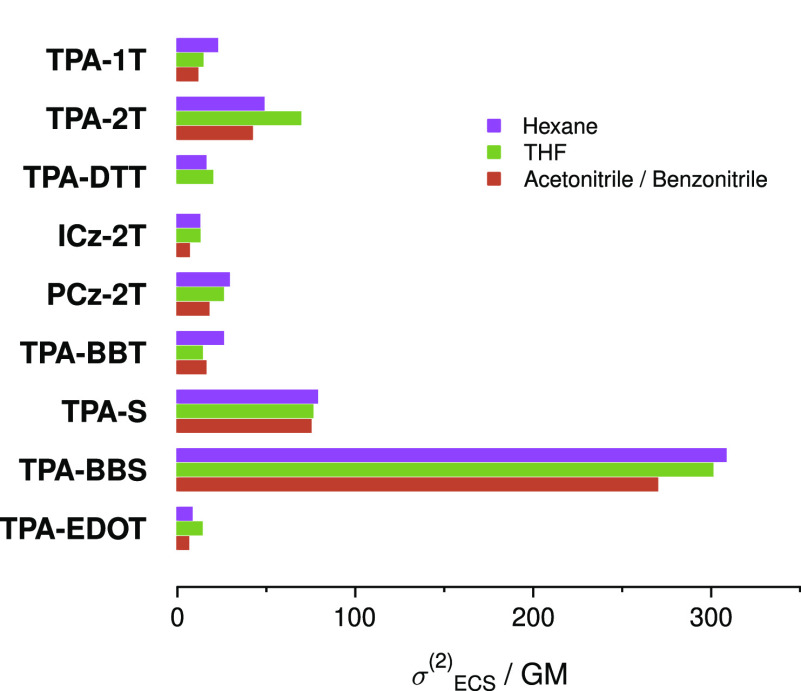
σ_ECS_^(2)^, calculated from eq 3 in solvents of varying polarity.

By combining the value at 780 nm (chosen due to its widespread use in commercial two-photon 3D printing systems) of the 2PA cross sections σ_780nm_^(2)^ (Table 1) and the ϕ_ISC_ values (assuming that the photoinitiation process starts from the triplet state), we can define the effective cross section (ECS)
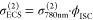
3and these are shown in Figure 5. As can be seen, most of the compounds’ σ_ECS_^(2)^s change with solvent polarity, making them less universal for use in a variety of polymer compounds. Additionally, the vast majority of the compounds show quite low σ_ECS_^(2)^, further limiting their applicability. The exceptions to this are the two selenium-containing compounds **TPA-S** and **TPA-BBS,** which, in addition to having high σ_ECS_^(2)^s, are affected minimally by polarity.

### 2PP Structuring Tests

**Figure 6:**
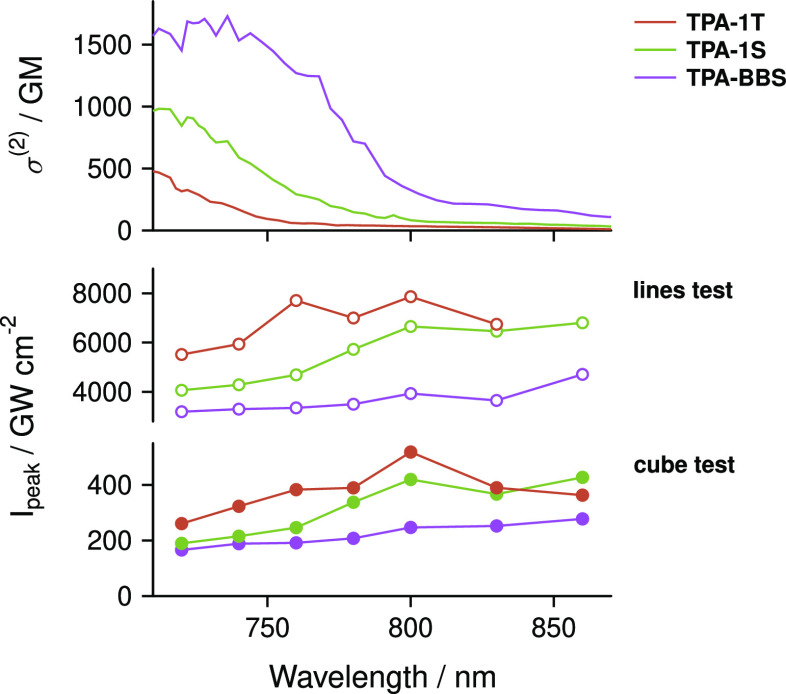
Spectral 2PP threshold tests were performed at wavelengths between 720 and 860 nm for **TPA-1 T**, **TPA-1S**, and **TPA-BBS** by structuring free-hanging single-voxel lines with a 63× objective and 100 μm × 100 μm × 100 μm cubes with a 10× objective. The 2P initiators were tested at a concentration of 0.5 μmol g^–1^. The upper panel shows the two-photon cross sections at the wavelengths tested. The middle panel shows the acquired *I*_peak,th_ for single-voxel line tests, and the lower panel shows the cube tests.

Due to the expected high 2PP efficiency of the studied compounds, 2PP threshold tests were performed at a very low concentration of 0.5 μmol g^–1^ (0.05–0.06 wt %) in an acrylate resin consisting of a 1:1 mixture of trimethylolpropane triacrylate (TTA) and ethoxylated-(20/3)-trimethylolpropane triacrylate (ETA) at wavelengths between 720 and 860 nm (Figure 6). In 2PP processing, resolution and throughput are counteracting parameters. Thus, depending on the desired outcome, different objectives can be used. In using high-magnification objectives, high-definition features can be fabricated. On the other hand, low-magnification objectives with a large field of view (FoV) enable high scanning speeds while sacrificing resolution, allowing the fabrication of designs in the mm and cm regime. Moreover, due to the larger FoV, low-magnification objectives allow the fabrication of parts larger than the FoV without or at a reduced number of overlap areas (stitches) when compared to high-magnification objectives. To investigate the sensitivity of selected compounds **TPA-1 T**, **TPA-S**, and **TPA-BBS** over a large spectral window for high-resolution and high-throughput structuring, threshold tests were performed with high (63×, 1.4 NA)- and low (10×, 0.4 NA)-magnification objectives.

Various approaches have been established to evaluate the polymerization threshold intensity *I*_th_ of a material for a given set of structuring parameters.^58−60^ Based on the characteristics of the objectives used, two different threshold tests have been performed. To determine the *I*_th_ with the 63× objective, free- hanging single-voxel lines of 10 μm length were structured in triplicates with a scanning speed of 100 mm s^–1^ between two preformed supporting pillars.^38,61^

Here, the average fabrication power *P*_avg_ was increased in steps of 2 mW (Figure S37), and the power that first yielded stable lines gave the polymerization threshold power *P*_th_ (Figure S38). In contrast, lines that were produced with the 10× objective showed poor mechanical stability, leading to unreliable results. Therefore, cubes with a side length of 100 μm were structured at variable power (steps of 2 mW) to determine *I*_th_ with the 10× objective (1000 mm s^–1^ scanning speed). The lowest *P*_avg_ at which polymerization could be observed was considered *P*_th_. To generalize our instrument-specific powers to values that can be compared across instrumentation, we calculated the laser pulse peak intensity (*I*_peak_).

Laser pulsing and the laser pulse peak intensity are crucial for second-order absorption and have a great impact on the polymerization threshold, hence making the conversion of instrument-specific powers to a nonspecific intensity necessary. The time-averaged power *P*_avg_ was measured after the objective to calculate *I*_peak,th_ for the polymerization thresholds, assuming a sech^2^-shaped pulse.^62,63^
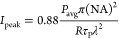
4

Equation 4 takes the average power (*P*_avg_), laser pulse repetition rate (*R*), pulse duration (τ_P_), fabrication wavelength (λ), and numerical aperture of the objective (NA) into account, which is crucial for spectral and objective comparison. Figure 6 shows the acquired spectral *I*_peak,th_ for both objectives. Despite the high scanning speed (1000 mm s^–1^) and a smaller NA, much lower *I*_peak,th_ values were required for inducing polymerization with the 10× objective. This can be explained by the more complex fabrication conditions present when creating a volumetric body, where additional radicals from adjacent irradiated areas affect the threshold.^36,64^ For both objectives and all tested compounds, the threshold behavior reflects the previously measured 2PA, showing a lower threshold at spectral regions with higher absorption and vice versa. However, with a maximum variation in the threshold of 2738 GW cm^–2^ (**TPA-S**, *I*_peak,th_ at 720 nm compared to 860 nm, middle panel of Figure 6) and overall low *I*_peak,th_, the synthesized compounds exhibited high 2PP initiation efficiency across the whole tested spectral window despite the high scanning speed (1000 mm s^–1^), proving them to be versatile and broadly applicable. As reference, 10× cube-tests were also performed with the commercial UV-photoinitiators **Irg369** and **BAPO** (Figures S40 and S41). Using a low concentration (1 μmol g^–1^), both initiators showed high 2PP thresholds, and for wavelengths longer than 780 nm (**BAPO**) and 800 nm (**Irg369**), it was not possible to achieve polymerization at the investigated conditions. *I*_peak,th_ values that were comparable to those acquired with the synthesized 2PA-initiators could only be achieved at a very high concentration of 100 μmol g^–1^, which is a factor of 200 higher than the concentration used for the synthesized 2PA initiators (0.5 μmol g^–1^).

To directly compare the efficiency of different 2PP photopolymer resins acquired under a variety of different experimental conditions, a dimensionless sensitivity figure-of-merit (FOM) was recently suggested in a publication by Kiefer et al.^36^ The aim was to establish a material property parameter that is independent of the measurement setup and conditions to be able to directly compare achieved threshold values. The FOM is based on certain experimental parameters including the focus velocity (ν), polymerization threshold power (*P*_th_), fabrication wavelength (λ), repetition rate (*R*), and pulse duration (τ_P_). The authors use their FOM to compare the efficiency of studied 2PP resins with already published data from literature. The crucial value to compare the efficiency of different resins is the minimum power *P*_th_, which has led to polymerization. In their own experiments, *P*_th_ was characterized as the power that has yielded a visible polymer dot. However, for other literature data, the criteria of *P*_th_ could vary. Although this methodology allows a simple comparison of experiments performed on different systems and conducted at quite different experimental parameters, this FOM does not take the PI concentration of a given 2PP resin into account.^i^ However, it indirectly depends on it as a lower 2PA-initiator concentration will give a lower FOM. Compared to the resin formulations listed by Kiefer et al.*,* the 2PP resins investigated in our study had a very low 2PA initiator concentration of 0.5 μmol g^–1^ (0.05–0.06 wt %), whereas the majority of the previously tested PIs were applied in concentrations above 1 wt %. However, despite the low 2PA initiator concentrations, the photoresins investigated in this study showed sensitivity FOMs between 14 and 382 for the line tests and between 2550 and 21,423 for the cubic structures (Supporting Information). Those values place the investigated resins for line tests at the wavelengths, at which they performed the best, among the 25% most efficient of the photoresins reviewed recently. The cube structuring threshold tests yielded sensitivity FOMs that were not reached by any of the previously reviewed photoresins despite the higher 2PA initiator concentrations used previously. Comparable values were achieved with the commercial PIs **Irg369** and **BAPO** only with a 200-fold higher molality (100 μmol g^–1^), showing the great potential of the presented PIs especially at low concentrations. In particular, using a low concentration is an advantage as this perturbs the properties of the monomer as little as possible, leading to material properties closer in line to the ideal one would predict from the monomer.

**Figure 7:**
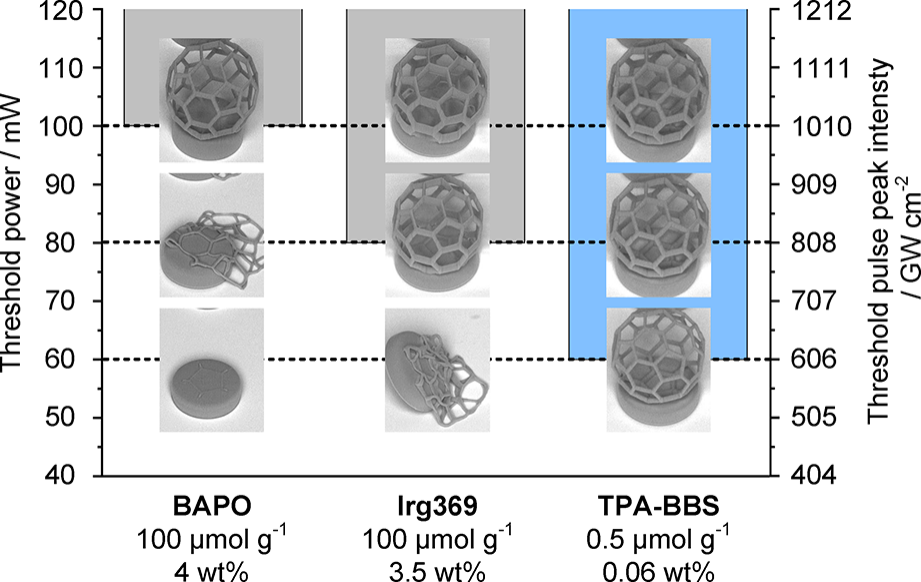
The superior two-photon initiation performance of **TPA-BBS** can be demonstrated by form-threshold tests. A fullerene-like structure (Ø = 250 μm) on a small platform was fabricated in triplicates at different laser powers to determine the threshold pulse peak intensity for formation of a stable structure. Despite its 200× lower molar concentration, **TPA-BBS** (0.5 μmol g^–1^) gives stable fullerene-structures already at 606 GW cm^–2^ compared to UV-photoinitiators (both 100 μmol g^–2^) **Irg369** (808 GW cm^–2^) and **BAPO** (1010 GW cm^–2^), due to its high σ_ECS_^(2)^ at 780 nm. Two-photon 3D printing system: NanoOne; objective: 10×/NA 0.4; wavelength: 780 nm; scanning speed: 600 mm s^–1^; hatch: 0.5 μm; slice distance: 2.5 μm.

Whereas *I*_peak,th_ acquired via scanning of isolated single lines may give an interesting comparison of the efficiency of different 2PA-initiator molecules, *I*_peak,th_ obtained by bulk polymerization of a certain photopolymer resin has more practical relevance. However, the cube tests give only a rough estimation of the actual pulse peak intensities required for 2P lithography. Hence, a form-threshold test was performed to determine *I*_peak,th_ (*P*_th_) for the formation of a stable structure (Figure 7 and Figure S42). The most efficient of the tested formulations based on **TPA-BBS** was compared with the highly concentrated UV-PI resins (**BAPO** and **Irg369**). To challenge the investigated photopolymer resin systems, a low-magnifying 10× objective (NA 0.4) was used. Due to its complexity, a fullerene-shaped object with a diameter of 250 μm was fabricated in triplicates. For better attachment to the glass substrate, a small platform (Ø = 250 μm, *h* = 40 μm) was printed underneath. A commercial two-photon 3D printing system (NanoOne, UpNano GmbH) operating at 780 nm was used. The structures were fabricated using the following parameters: scanning speed: 600 mm s^-1^; hatch: 0.5 μm; slicing distance: 2.5 μm. Despite their low σ^(2)^ values,^65^ commercial type I UV-initiators Irg369 (Figure S46) and BAPO (Figure S47) could be applied as photoinitiators for 2PP at very high concentrations (100 μmol g^–1^), giving stable structures at 808 GW cm^–2^ (80 mW) and 1010 GW cm^–2^ (100 mW) respectively. In contrast, due to its high σ_ECS_^(2)^ at 780 nm, TPA-BBS yields stable structures already at 606 GW cm^–2^ (60 mW), even when a 200× lower molar concentration (0.5 μmol g^–1^) was used (Figure S44). However, when doubling the concentration of TPA-BBS to 1.0 μmol g^–1^, the form-threshold can be further decreased to 404 GW cm^–2^ (40 mW) (Figure S45).^40^

For comparison, the type II initiator **ITX** was also tested (Figure S43). When used at a concentration of 100 μmol g^–1^ without coinitiator, the form-threshold was determined to be 1020 GW cm^–2^ (120 mW) (Figure S48). In contrast, when applied together with amine coinititator **MDEA** at an equimolar concentration, a noncollapsing structure formed at a laser power of as low as 404 GW cm^–2^ (40 mW) (Figure S49). However, no shape fidelity could be achieved since extreme overpolymerization occurred at any laser intensity used, leading to very distorted structures and a complete loss in *z* resolution. When the concentration of both **ITX** and **MDEA** was reduced to 25 μmol g^–1^, the shape-threshold was lowered to 1020 GW cm^–2^ (120 mW) (Figure S50). However, the resolution remained low due to overpolymerization along the *z* axis. Such overpolymerization is a phenomenon typically occurring when printing overhanging structures using UV–Vis light-based 3D printing techniques such as stereolithography (SLA) and digital light processing (DLP).^66^

However, it is rather unusual to observe in 2PP, where the polymerization region should be confined within the voxel.

In the present case, the tertiary amine most likely increases the polymerization efficiency and diminishes termination reactions by decreasing the concentration of dissolved molecular oxygen due to its ability to react with generated peroxyl radicals.^67^ Hence, the polymerization is not confined to the region of the voxel anymore but goes beyond, causing a decrease in resolution.

These results clearly highlight the fact that a mere consideration of the polymerization threshold intensity, at which a visible polymer dot has been formed, is not sufficient to benchmark the performance of a 2PP resin since with all investigated photoresist systems, polymerization of the disk platform, or fragments thereof, occurred at laser intensities at which no stable fullerene structures could be fabricated.

## Conclusions

A series of soluble, symmetric chalcogenophenes bearing hexyl-substituted triphenylamines, indolocarbazoles, or phenylcarbazoles was synthesized as two-photon polymerization initiators. Photophysical analysis showed a strong influence of the chalcogenophene unit on the two-photon absorption cross section, rendering the two selenium-containing compounds **TPA-S** and **TPA-BBS** promising new photoinitiators. Both compounds were tested as 2PA initiators in an acrylate resin formulation; structuring and threshold tests showed their efficiency and versatility for a broad spectral window and different fabrication conditions, including the use of high- and low-magnification objectives. The photoinitiators reported here outperform the commercial UV-initiators **Irg369** and **BAPO** as well as the sensitizer **ITX** at both lower powers and lower concentrations. In particular, by increasing the concentration of **TPA-BBS**, the threshold can be further reduced, showing the great potential of this compound for application in 2PP systems where much less laser power *P*_ave_ is available. Such applications include systems with more economical low-power lasers, which, due to the significant reduction of cost and footprint, allow the acquisition and operation of multiple 2PP systems as well as advanced technologies permitting ultrafast 2PP by dynamic optical tuning of the voxel size,^40,68^ simultaneous polymerization using multiple laser foci,^28,69,70^ or focal field engineering.^71^ All these applications further benefit from the use of high-performance 2P photoinitiators, especially if operated at elevated scanning speeds or when low-magnification objectives are used. Overall, due to these innovations inducing significant cost reductions of 2PP printed parts, the industrial-scale manufacturing of highly resolved micro- to even macroscale parts using the materials outlined here could be a future possibility.

## Experimental Section

### Molecular Synthesis and Characterization

Detailed synthetic procedures as well as ^1^H/^13^C NMR spectra and HR-MS analysis data are given in the Supporting Information.

### One-Photon Absorption and Fluorescence Spectroscopy

Absorption spectra were recorded on a Cary 50 absorption spectrometer. Emission and excitation spectra were collected on a Horiba Fluoromax at a controlled temperature of 20 °C. Absorption and emission spectra were baseline-corrected by subtracting the spectra of the corresponding pure solvent. Absorption spectra were obtained using samples with a maximal absorbance of 1, while the maximal absorbance for obtaining emission spectra was kept below 0.3. The emission spectra were corrected for the wavelength sensitivity of the applied spectrometer by using a set of secondary fluorescence standards.^72^ Emission quantum yields were obtained using Rhodamine 6G in degassed ethanol as a secondary emission standard (fluorescence quantum yield = 0.95)^73^ and eq 5
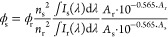
5where ϕ_*x*_ is the fluorescence quantum yield of the sample (s) and the reference (r), and *I_x_* denotes the corresponding integrated intensity of the fluorescence spectrum. *A_x_* is the absorbance at the excitation wavelength, with *n_x_* denoting the refractive index of the sample (s) and reference (r) solution. The value to correct for the inner filter effect of the spectrometer is 0.565.^74,75^

### Two-Photon Absorption Spectroscopy

Two-photon cross sections were determined via two-photon excitation spectra using a setup similar to the one described by Makarov et al.,^57^ which has been described previously.^76^ The two-photon cross section at a given wavenumber, σ_s_^(2)^(ν̃), were calculated as follows:^57^
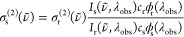
6Here *I_x_*(ν̃, λ_obs_) is the (two-photon induced) fluorescence intensity at excitation wavenumber ν̃ and observation wavelength λ_obs_ for either sample or reference (*x* ∈ {s, *r*}). *c_x_* and ϕ_*x*_(λ_obs_) are the concentration and differential fluorescence quantum yield (at the observation wavelength) of the sample and reference. Coumarin 153 in DMSO and rhodamine 6G in methanol were used as reference as in the work of the Rebane group.^77^

### Time-Correlated Single Photon Counting and Fluorescence Up-Conversion

Lifetimes above 300 ps were measured on a home-built time-correlated single photon counting device using a 400 or 470 nm laser diode (PicoQuant) as an excitation source.^78^ The time resolution, as judged from the full width at half maximum of the instrument response function (IRF) recorded with a scattering LUDOX solution, was ∼200 ps. The samples, located in a 10 mm × 10 mm quartz cell, had an absorbance of 0.1–0.2 at the excitation wavelength. The fluorescence time profiles were analyzed by iterative reconvolution of the IRF, with a single exponential function.

Lifetimes below 300 ps were measured using fluorescence up-conversion, with a setup previously described.^79,80^ Briefly, the samples were excited at 400 nm using the frequency-doubled output of a Kerr lens mode-locked Ti:Sapphire laser (Mai Tai, Spectra-Physics). The polarization of the pump pulses was at a magic angle relative to that of the gate pulses at 800 nm. The pump intensity on the sample was of the order of 5 μJ cm^–2^, and the FWHM of the IRF was ∼210 fs. Sample solutions with absorbances of 0.1–0.2 at the excitation wavelength were located in a rotating cell with a 500 μm optical path length.

### Transient Absorption Spectroscopy

The setups for fs and ns transient absorption (TA) have been described previously.^81,82^ For fs-TA, 400 nm was used as the excitation wavelength, and the instrument response function had a full width at half maximum of ∼150–300 fs as derived from Optical Kerr effect measurements in hexane, THF, benzonitrile, and acetonitrile. Samples were excited with a pump intensity of ∼1–2 mJ cm^–2^. Samples were saturated and then bubbled with nitrogen during the experiment to constantly refresh the excitation volume, thus avoiding sample decomposition. Changes in the sample concentration due to degradation and/or solvent evaporation were negligible as judged from absorption spectra before and after the experiments. Nanosecond TA experiments were performed with 355 nm excitation using the same procedures as for the fs-TA. Species-associated decay spectra and time constants were extracted using a global lifetime analysis.^83^

### Structuring Tests

#### Resin Formulations

Resin formulations of the compounds **TPA-1 T**, **TPA-S**, and **TPA-BBS** at concentrations of 0.5 μmol g^–1^ (**TPA-BBS** also 1.0 μmol g^–1^) were prepared in a 1:1 mixture of trimethylolpropane triacrylate (TTA, Genomer 1330) and ethoxylated-(20/3)-trimethylolpropane triacrylate (ETA, Sartomer 415). Acetone was used as co-solvent and later removed in a vacuum. Reference resins were prepared from commercial UV-photoinitiators at concentrations of 100 μmol g^–1^ (**BAPO** (4 wt %), **Irg369** (3.5 wt %), **ITX** (2.5 wt %), and **MDEA** (1.1 wt %)) and 1 μmol g^–1^ (**BAPO** (0.04 wt %) and **Irg369** (0.035 wt %)). The formulation containing 100 μmol g^–1^**ITX** and **MDEA** was further diluted to a concentration of 25 μmol g^–1^.

#### Two-Photon Structuring Tests

2PP threshold tests (nanowires and cubes) were performed with a 2PP setup based on a tunable Ti:Sapphire femtosecond NIR laser (Mai Tai eHP DeepSee, Spectra-Physics) with a pulse width of 90–70 fs, depending on the wavelength used, and a repetition rate of 80 MHz. The setup has been described in detail by Dobos et al.^23^ Experiments were performed at wavelengths of 700, 720, 740, 760, 780, 800, 830, and 860 nm. The laser is equipped with a precompensation unit (DeepSee, Spectra-Physics) that allows compensation for the dispersion introduced downstream by the optical system of the 2PP setup. Since the efficiency of the polymerization depends on the pulse width, the minimal achievable pulse width for each wavelength was determined by threshold tests. Hence, threshold tests were performed at constant parameters varying only the DeepSee setting. The setting that gave the lowest achievable polymerization threshold was considered the optimal setting of the DeepSee module and the lowest achievable pulse width for a certain wavelength. The respective pulse widths for optimized DeepSee Module settings were then measured with an autocorrelator that was coupled into the system. Time averaged threshold powers were measured with a powermeter (Fieldmax II, Coherent Inc.) after the objective. Nanowires were created with a 63× oil-immersion objective (Plan-Apochromat, NA 1.4, Zeiss) using a scanning speed of 100 mm s^–1^. The supporting pillars were structured with 150 mm s^–1^. Cube-tests (100 μm × 100 μm × 100 μm) were performed with a 10× objective (UPLSAPO10X, NA 0.4, Olympus) at a fabrication speed of 1000 mm s^–1^, a line distance (hatch, Δ*xy*) of 0.3 μm, and a slicing distance (Δ*z*) of 0.4 μm. The microstructures were written on glass coverslips that were functionalized with methacrylate prior to the experiments.^84^ After structuring, the samples were developed for several hours in IPA and washed afterwards with HMDS to reduce surface tension during evaporation. The microstructures were then imaged with a differential interference contrast microscope (LSM 700, Zeiss). The figure of merit (FOM) established by Kiefer et al.^36^ was calculated for each threshold value with the following specifications: repetition rate: 80 MHz; pulse width: 90 fs (720 nm), 75 fs (740 nm), and 70 fs (other wavelengths); scanning speed: 100 mm s^–1^ (wires) and 1000 mm s^–1^ (cubes); numerical aperture: 0.4 (10× objective) and 1.4 (63× objective).

### Two-Photon Polymerization Form-Threshold Tests

Threshold tests (fullerene shapes) were performed using a NanoOne high-resolution 3D printing system (UpNano GmbH, Austria) equipped with a 10× air objective (NA 0.4, UPLXAPO10X, Olympus) in vat mode. Here, the laser (80 MHz repetition rate, 90 fs pulse length, and 780 nm wavelength) is focused through a high-precision cover glass into a material vat containing the resin and maintained at a constant height above the glass window. Methacrylized^84^ borosilicate glass substrates were used. For layer-wise 3D structuring, the laser is scanned along the *xy* plane by a galvanometer scanner, and the objective together with the vat is lowered along the *z* axis using a piezo stage. All microstructures were fabricated in triplicates at a scanning speed of 600 mm s^–1^ using laser powers from 10–200 mW (measured after the objective using a thermal power sensor S175C, Thorlabs). The *x* and *y* planes were sliced alternately in the *x*- and *y*-direction using a line distance (hatch, Δ*xy*) of 0.50 μm and a slicing distance (Δ*z*) of 2.5 μm. The nominal voxel size was estimated to be 0.73 μm laterally and 8.81 μm along the *z* axis.^85^ The microstructures were then imaged by scanning electron microscopy (SEM) in variable-pressure mode using a BSE detector (FlexSEM 1000, Hitachi).

## Supporting Information Available

Synthetic procedures; ^1^H and ^13^C NMR spectra of compounds **TPA-1T**, **TPA-2T**, **TPA-DTT**, **ICz-2T**, **PCz-2T**, **TPA-BBT**, **TPA-S**, **TPA-BBS**, and **TPA-EDOT**; further structuring tests; quantum chemical calculations; photophysical data (PDF)

## Footnotes

i The authors argue that a limitation of their methodology is the fact that a certain FOM would be lower than what could be achievable if the investigated photoresist system is used at a concentration below the solubility limit. Nevertheless, operating at the solubility limit is quite impractical as highly saturated solutions are temperature-sensitive. Especially commercial resins are most likely not formulated close to the solubility limit.
