# Supplementary material for: Beyond the Threshold: A Study of Chalcogenophene-Based Two-Photon Initiators
Source: Chem Mater. 2022 Mar 22;34(7):3042–52. doi: 10.1021/acs.chemmater.1c04002 (PMC9009090; doi:10.1021/acs.chemmater.1c04002)
Supplement: Supplementary file 1 — cm1c04002_si_001.pdf [file cm1c04002_si_001.pdf]

# Beyond the Threshold: A Study of Chalcogenophene-Based Two-Photon Initiators

Markus Lunzer,[†,‡,ǁ,¶*] Joseph S. Beckwith,[§,¶] Franziska Chalupa-Gantner,[‡] Arnulf Rosspeintner,[§] Giuseppe Licari,[§] Wolfgang Steiger,[‡] Christian Hametner,[†] Robert Liska,[†] Johannes Fröhlich,[†] Eric Vauthey,[§] Aleksandr Ovsianikov,[‡] and Brigitte Holzer[†*]

[†]Institute of Applied Synthetic Chemistry, TU Wien, Getreidemarkt 9/163, 1060 Vienna, Austria

[‡]Institute of Materials Science and Technology, TU Wien, Getreidemarkt 9/308, 1060 Vienna, Austria

[ǁ]UpNano GmbH, Modecenterstraße 22/D36, 1030 Vienna, Austria

[§]Department of Physical Chemistry, University of Geneva, 30 Quai Ernest-Ansermet, 1211 Geneva, Switzerland

markus.lunzer@upnano.at

brigitte.holzer@tuwien.ac.at

**A) Materials and Methods**

**B) Synthesis**

**C) NMR Spectra**

**D) Computational Chemistry**

**E) Structuring Tests**

**F) Photophysical Data**

**G) References**

# A) Materials and Methods

2,3-Dihydrothieno[3,4-*b*]-1,4-dioxine (EDOT), anhydrous *N*,*N*-dimethyl-formamide (DMF), anhydrous *N*,*N*-dimethylacetamide (DMAc), n-butyllithium solution (2.5 M in hexanes), n-hexyllithium solution (n-HexLi, 2.3 M in hexanes), hexamethyldisilazan (HMDS) and methyl diethanolamine (MDEA) were purchased from Sigma-Aldrich. Commercial photoinitiators phenylbis(2,4,6-trimethylbenzoyl)-phosphine oxide (BAPO, CAS 162881-26-7), 2-benzyl-2-(dimethylamino)-4-morpholinobutyro-phenone (Irg369, CAS 119313-12-1) and 2-isopropylthioxanthone (ITX, CAS 5495-84-1) were purchased from TCI Europe. All substances purchased from commercial sources were used as received without further purification. Isopropyl pinacol borate (CAS 61676-62-8),[1] allyl[1,3-bis(2,6-diisopropylphenyl)imidazol-2-ylidene]chloropalladium(II) ((IPr)Pd(allyl)Cl, CAS 478980-03-9),[2] 3,6-dihexyl-9*H*-carbazole (CAS 1131605-21-4),[3] 1,1'-benzo[1,2-*b*:4,5-*b'*]dithiophene-2,6-diylbis(1,1,1-trimethylstannane) (CAS 1242077-07-1),[4] 1,1'-benzo[1,2-*b*:4,5-*b'*]diselenophene-2,6-diylbis(1,1,1-trimethylstannane) (CAS 1465181-53-6),[5] 1,1'-(2,5-selenophenediyl)bis(1,1,1-trimethylstannane) (CAS 220770- 41-2),[6] 2,5-dibromothiophene (CAS 3141-27-3),[7] 5,5'-dibromo-2,2'-bithiophene (CAS 4805-22-5)[8] and 2,6-dibromodithieno[3,2-*b*:2',3'-*d*]thiophene (CAS 67061-69-2)[9] were synthesized according to literature. Isopropylalcohol (IPA) and dimethylsulfoxide (DMSO) were used in p.a. quality. Acrylate resins trimethylolpropane triacrylate (TTA, SR351, CAS 15625-89-5) and ethoxylated-(20/3)-trimethylolpropane triacrylate (ETA, SR 415, CAS 28961-43-5) were received from Sartomer Europe. Technical grade solvents were distilled prior to use. Analytical TLC was performed on Merck silica gel 60 F254 plates. Chromatographic separations at preparative scale were carried out on silica gel (Merck silica gel 60, 40 - 63 µm). Nuclear magnetic resonance (NMR) spectra were obtained using a Bruker DPX-200 or Avance DRX-400 Fourier transform spectrometer operating at the following frequencies: DPX-200: 200.1 MHz ($^1$H) and 50.3 MHz ($^{13}$C); DRX-400: 400.1 MHz ($^1$H) and 100.6 MHz ($^{13}$C). The chemical shifts are reported in delta (δ) units, parts per million (ppm) downfield from tetramethylsilane using residual solvent signals for calibration. Coupling constants are reported in Hertz; multiplicity of signals is indicated by using following abbreviations: s=singlet, d=doublet, t=triplet, q=quartet. The multiplicity of $^{13}$C signals was obtained by measuring JMOD spectra. High-resolution mass spectra (HRMS) were acquired using a Thermo Scientific LTQ Orbitrap XL hybrid FTMS (Fourier Transform Mass Spectrometer) equipped with Thermo Fischer Exactive Plus Orbitrap (LC-ESI+) and a Shimadzu IT-TOF Mass Spectrometer.

## B) Synthesis

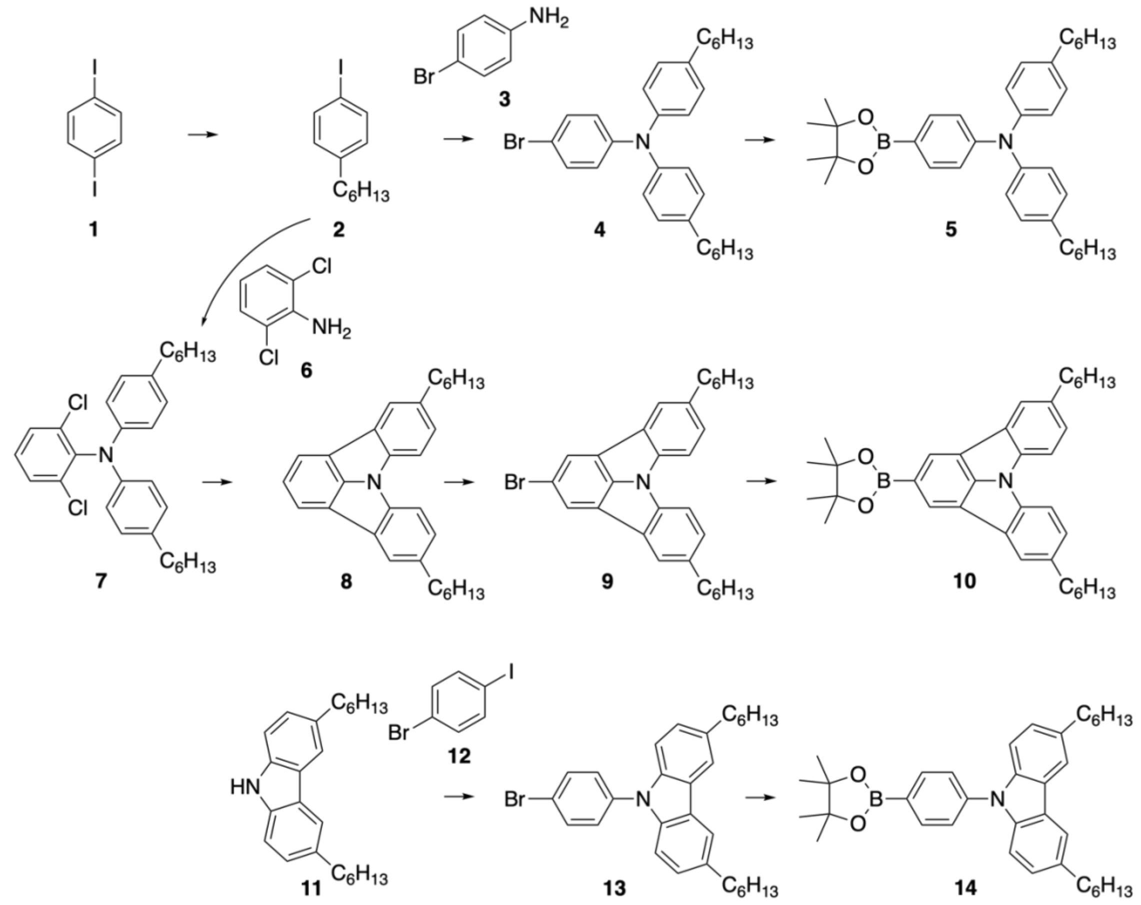

4-Iodo-1-hexylbenzene (**2**). The synthesis of **2** was adapted from a procedure described by Doszczak.[10] 1,4-Diiodobenzene **1** (49.49 g, 150.0 mmol, 1.00 eq) was suspended in anhydrous $Et_2O$ (150 mL) under argon atmosphere and cooled below -78 ºC. Subsequently n-HexLi (66 mL, 151.5 mmol, 1.01 eq) was added dropwise over 2 hours to the reaction mixture. After complete addition the suspension was stirred for 15 min below -78 ºC and then warmed to -20 ºC. Thereafter it was cooled to -70 ºC and 1-iodohexane (39.76 g, 187.5 mmol, 1.25 eq) was slowly added before the cooling bath was replaced by an ice-bath and the suspension was stirred overnight. The reaction mixture was quenched with 100 mL water and extracted repeatedly with $Et_2O$. The combined organic layers were dried over $Na_2SO_4$, filtered and dried *in vacuo*. Remains of 1-iodohexane were removed on a high-vacuum rotary evaporator before the crude product was fractionated by high-vacuum distillation (88 - 98 °C, 0.0018 mbar). Compound **2** was obtained in sufficient purity as a pinkish liquid (28.88 g, 100.2 mmol, 67%). $^1$H NMR (200 MHz, $CDCl_3$): δ = 7.62 - 7.55 (m, 2 H), 6.95 – 6.91 (m, 2 H), 2.54 (t, J = 7.6 Hz, 2 H), 1.61 - 1.50 (m, 2 H), 1.42 - 1.18 (m, 6H), 0.88 (t, J = 7.7, 3 H) ppm. $^{13}$C NMR (50 MHz, $CDCl_3$): δ = 142.4, 137.2, 130.5, 90.5, 35.4, 31.6, 31.2, 28.8, 22.5, 14.1 ppm.

General procedure for the synthesis of triphenylamines **4** and **7** according to Goodbrand.[11] Substituted aniline (1.0 eq), iodobenzene (2.2 eq), KOH (7.8 eq), Cu(I)Cl (0.04 eq) and 1,10-phenanthroline monohydrate (0.04 eq) were suspended in anhydrous toluene in a round-bottomed flask equipped with a Dean-Stark trap under a reflux condenser and an argon balloon. The reaction mixture was stirred magnetically at reflux until GC-MS analysis showed full conversion. After cooling the reaction mixture to room temperature water was added to the stirred suspension until the solid KOH residue was dissolved. The aqueous phase was extracted with toluene repeatedly. The combined organic layers were washed with brine, dried over $Na_2SO_4$ and filtered before the toluene was removed by rotary evaporation. Purification was performed as stated in the detailed descriptions.

4-Bromo-*N*,*N*-bis(4-hexylphenyl)benzenamine (**4**). 4-Bromoaniline **3** (9.77 g, 57 mmol, 1.0 eq), 1-hexyl-4-iodobenzene **2** (36.00 g, 125 mmol, 2.2 eq), KOH (24.87 g, 443 mmol, 7.8 eq), Cu(I)Cl (230 mg, 2.3 mmol, 0.04 eq) and 1,10-phenanthroline monohydrate (450 mg, 2.3 mmol, 0.04 eq) were refluxed in 120 mL of anhydrous toluene until TLC showed full conversion (4 d). The crude product was flashed over a pad of silica. Remaining impurities were distilled off (120 °C, 0.24 mbar) and **4** was obtained as yellow oil (24.17 g, 49 mmol, 86%). $R_f$ = 0.44 (light petroleum). $^1$H NMR (200 MHz, $CH_2Cl_2$): δ = 7.31 - 7.24 (m, 2 H), 7.11 - 7.06 (m, 4 H), 6.98 - 6.94 (m, 4 H), 6.90 - 6.83 (m, 2 H), 2.56 (t, J = 7.9 Hz, 4 H), 1.63 - 1.52 (m, 4 H), 1.42 - 1.31 (m, 12 H), 0.89 (t, J = 6.6 Hz, 6 H) ppm. $^{13}$C NMR (50 MHz, $CDCl_3$): δ = 147.4 (s), 145.0 (s), 138.0 (s), 131.9 (d), 129.2 (d), 124.5 (d), 124.0 (d), 113.6 (s), 35.4 (t), 31.7 (t), 31.5 (t), 29.1 (t), 22.6 (t), 14.1 (q) ppm.

2,6-Dichloro-*N*,*N*-bis(4-hexylphenyl)benzenamine (**7**). The synthesis of **7** followed the general procedure, 2,6-dichloroaniline **6** (4.86 g, 30 mmol, 1.0 eq), 1-hexyl-4-iodobenzene **2** (17.79 g, 61.7 mmol, 2.06 eq), KOH (13.13 g, 234 mmol, 7.8 eq), Cu(I)Cl (120 mg, 1.2 mmol, 0.04 eq) and 1,10-phenanthroline monohydrate (240 mg, 1.2 mmol, 0.04 eq) were refluxed in 43 mL anhydrous toluene until GC-MS showed full conversion (8 d). After work-up according to the general procedure, the crude product (15.94 g) was flashed over silica gel (420 g) applying light petroleum. Impure fractions were further purified by distilling impurities off *via* Kugelrohr distillation (140 °C, 0.014 mbar) yielding **7** as a yellowish oil (5.09 g, 10.5 mmol, 35%). $R_f$ = 0.47 (light petroleum). $^1$H NMR (200 MHz, $CDCl_3$): δ = 7.44 - 7.39 (m, 2 H), 7.24 - 7.16 (m, 1 H), 9.12 – 6.98 (m, 4 H), 6.98 - 6.84 (m, 4 H), 2.57 (t, J = 7.6 Hz, 4 H), 1.69 - 1.53 (m, 4 H), 1.39 - 1.28 (m, 12 H), 0.91 (t, J = 6.6 Hz, 6 H) ppm. $^{13}$C NMR (100 MHz, $CDCl_3$): δ = 143.1 (s), 140.6 (s), 137.2 (s), 136.4 (s), 129.5 (d), 128.8 (d), 128.1 (d), 120.3 (d), 35.3 (t), 31.7 (t), 31.5 (t), 29.1 (t) 22.6 (t), 14.1 (q) ppm.

9-(4-Bromophenyl)-3,6-dihexyl-9*H*-carbazole (**13**). The synthesis of **13** was realized according to Aizawa.[12] **11** (4.64 g, 13.8 mmol, 1.0 eq), 1-bromo-4-iodobenzene **12** (4.30 g, 15.2 mmol, 1.1 eq), Cu(0) powder (2.73 g, 42.9 mmol, 3.1 eq) and $K_2CO_3$ (5.93 g, 42.9 mmol, 3.1 eq) were refluxed in 30 mL anhydrous DMF under argon atmosphere until TLC (light petroleum) showed full conversion (20 h). After cooling to room temperature, the reaction mixture was suspended in DCM and filtered over Celite. The solution was washed with water, dried over $Na_2SO_4$ and filtered before it was concentrated under high vacuum. **13** was isolated by column chromatography (200 g silica gel, light petroleum) as a white solid (6.08 g, 12.4 mmol, 90%). $R_f$ = 0.51 (light

petroleum). $^1$H NMR (200 MHz, $CDCl_3$): δ = 7.91 – 7.81 (m, 2 H), 7.68 - 7.61 (m, 2 H), 7.47 - 7.35 (m, 2 H), 7.24 - 7.14 (m, 4 H), 2.74 (t, J = 7.5 Hz, 4 H), 1.67 (quin, J = 7.5 Hz, 4 H), 1.42 - 1.23 (m, 12 H), 0.85 (t, J = 6.9 Hz, 6 H) ppm. $^{13}$C NMR (50 MHz, $CDCl_3$): δ = 139.2 (s), 137.3 (s), 134.8 (s), 133.0 (d), 128.4 (d), 126.6 (d), 123.6 (s), 120.3 (s), 119.6 (d), 109.2 (d), 36.0 (t), 32.3 (t), 31.8 (t), 29.0 (t), 22.7 (t), 14.1 (q) ppm. HR-ESI-FTMS $[M+H]^+$ m/z calcd. 490.2104 for $C_{30}H_{37}BrN^+$, found 490.2098.

5,11-Dihexylindolo[3,2,1-*jk*]carbazole (**8**). The synthesis of **8** followed an adapted protocol by Campeau.[13] In order to adjust the water content of the solvent to 1000 ppm deionized water (22 mg) was added to anhydrous DMAc. **7** (4.66 g, 9.6 mmol, 1.0 eq) and $K_2CO_3$ (2.68 g, 19.3 mmol, 2.0 eq) were suspended in 25 mL degassed DMAc before (IPr)Pd(allyl)Cl (276 mg, 0.5 mmol, 5 mol%) was added in a countercurrent flow of argon and the reaction mixture was heated to 130 °C. The reaction progress was monitored by GC-MS analysis. After 3 days of heating no further conversion could be observed and additional catalyst (276 mg, 0.5 mmol, 5 mol%) was added and the temperature was raised to 160 °C. After 4 hours the reaction was complete. The reaction mixture was poured onto water and extracted with DCM. The combined organic layers were dried over $Na_2SO_4$, filtered and dried under reduced pressure. The crude product (7.52 g) was flashed over a silica pad (60 g silica gel, light petroleum : DCM, 2 : 1) yielding **8** (3.70 g, 9.0 mmol, 94%) as an off-white powder. $R_f$ = 0.52 (light petroleum). $^1$H NMR (400 MHz, $CDCl_3$): δ = 8.02 (d, J = 7.4 Hz, 2 H), 7.97 – 7.91 (m, 2 H), 7.77 (d, J = 8.2 Hz, 2 H), 7.56 (t, J = 8.1 Hz, 1 H), 7.39 – 7.32 (m, 2 H), 2.82 (t, J = 7.6 Hz, 4 H), 1.76 (quin, J = 7.6 Hz, 4 H), 1.45 - 1.35 (m, 12 H), 0.93 (t, J = 7.0 Hz, 6 H) ppm. $^{13}$C NMR (100 MHz, $CDCl_3$): δ = 144.3 (s), 137.0 (s), 136.2 (s), 130.0 (s), 127.0 (d), 122.8 (d), 122.4 (d), 119.1 (d), 118.5 (s), 111.5 (d), 36.1 (t), 32.1 (t), 31.8 (t), 29.0 (t), 22.7 (t), 14.1 (q) ppm. HR-ESI-FTMS $[M+H]^+$ m/z calcd. 410.2842 for $C_{30}H_{36}N^+$, found 410.2836.

2-Bromo-5,11-dihexylindolo[3,2,1-*jk*]carbazole (**9**). The bromination of **8** was performed according to Bintinger.[13] Dihexylindolocarbazole **8** (3.50 g, 8.5 mmol, 1.0 eq) was suspended in a 1:1 mixture of $CHCl_3$ and AcOH (42 mL) under argon atmosphere and heated to 55 °C. To this suspension *N*-bromosuccinimide (1.52 g, 8.5 mmol, 1.0 eq) was added in small portions over a period of 2.5 hours. During the addition a white precipitate formed. The mixture was further stirred for 30 min before it was poured onto 300 mL 2 N NaOH solution and extracted with DCM. **9** was purified by recrystallization from acetonitrile and obtained as fine yellowish needles (3.54 g, 7.2 mmol, 85%). $R_f$ = 0.58 (light petroleum). $^1$H NMR (400 MHz, $CDCl_3$): δ = 8.06 (s, 2H), 7.85 - 7.79 (m, 2 H), 7.72 - 7.64 (m, 2 H), 7.38 - 7.30 (m, 2 H), 2.78 (t, J = 7.8 Hz, 4 H), 1.73 (quin, J = 7.1 Hz, 4 H), 1.42 - 1.33 (m, 12 H), 0.91 (t, J = 6.7 Hz, 6 H) ppm. $^{13}$C NMR (100 MHz, $CDCl_3$): δ = 142.4 (s), 137.3 (s), 136.6 (s), 129.1 (s), 127.7 (d), 122.9 (d), 122.0 (d), 119.6 (s), 115.3 (s), 111.7 (d), 36.0 (t), 32.0 (t), 31.8 (t), 29.0 (t), 22.6 (t), 14.1 (q) ppm. HR-ESI-FTMS $[M+H]^+$ m/z calcd. 488.1947 for $C_{30}H_{35}BrN^+$, found 488.1946.

General procedure for the synthesis of **5**, **10** and **14** according to Anémian.[14] The brominated precursor (1.0 eq) was dissolved in anhydrous THF (~0.2 M) under argon atmosphere and cooled below -78 °C. To the stirred solution n-BuLi (1.2 eq) was added dropwise and the reaction mixture was stirred below -65 °C for at least 1.5 h before isopropyl pinacol borate (1.2 eq) was added. After warming the solution to room temperature slowly it

was stirred overnight. THF was removed *in vacuo* before the residue was partitioned between DCM and water. The aqueous phase was extracted with DCM. The combined organic layers were dried over $Na_2SO_4$ followed by evaporation of the solvent *in vacuo*.

*N*,*N*-Bis(4-hexylphenyl)-4-(4,4,5,5-tetramethyl-1,3,2-dioxaborolan-2-yl)benzenamine (**5**). Starting from bromide **4** (13.21 g, 26.8 mmol, 1.0 eq), n-BuLi (11.8 mL, 29.4 mmol, 1.1 eq) and isopropyl pinacol borate (5.47 g, 29.4 mmol, 1.1 eq) in 120 mL of anhydrous THF **5** (11.11 g, 20.5 mmol, 77%) was isolated as a pale yellowish oil by column chromatography (350 g silica gel, light petroleum : $Et_2O$, 2 $\rightarrow$ 3%). $R_f$ = 0.26 (light petroleum : DCM, 17 : 3). $^1$H NMR (400 MHz, $CDCl_3$): $\delta$ = 7.66 - 7.60 (m, 2 H), 7.10 - 6.93 (m, 10 H), 2.57 (t, J = 7.8 Hz, 4 H), 1.68 - 1.54 (m, 4 H), 1.39 - 1.25 (m, 24 H), 0.90 (t, J = 6.5 Hz, 6 H) ppm. $^{13}$C NMR (100 MHz, $CDCl_3$): $\delta$ = 151.0 (s), 145.0 (s), 138.2 (s), 135.7 (d), 129.2 (d), 125.1 (d), 120.7 (d), 83.4 (s), 35.4 (t), 31.7 (t), 31.4 (t), 29.1 (t), 24.8 (q), 22.6 (t), 14.1 (q) ppm. HR-ESI-FTMS $[M+H]^+$ m/z calcd. 540.4007 for $C_{36}H_{51}BNO_2^+$, found 540.4005.

5,11-Dihexyl-2-(4,4,5,5-tetramethyl-1,3,2-dioxaborolan-2-yl)indolo[3,2,1-*jk*]carbazole (**10**). Starting from bromide **9** (3.17 g, 6.5 mmol, 1.0 eq), n-BuLi (3.1 mL, 7.8 mmol, 1.2 eq) and isopropyl pinacol borate (1.45 g, 7.8 mmol, 1.2 eq) in 110 mL anhydrous THF, **10** was isolated by recrystallization from acetonitrile and subsequent flash chromatography (34 g silica gel, light petroleum : ethyl acetate, 0 $\rightarrow$ 7%) as a grayish solid (2.02 g, 3.8 mmol, 58%). $R_f$ = 0.53 (light petroleum : DCM, 7 : 3). $^1$H NMR (400 MHz, $CD_2Cl_2$): $\delta$ = 8.51 (s, 2 H), 8.01 - 7.94 (m, 2 H), 7.82 - 7.94 (m, 2 H), 7.42 – 7.34 (m, 2 H), 2.82 (t, J = 7.8 Hz, 4 H), 1.74 (quin, J = 7.6 Hz, 4 H), 1.44 - 1.34 (m, 24 H), 0.92 (t, J = 6.8 Hz, 6 H) ppm. $^{13}$C NMR (100 MHz, $CD_2Cl_2$): $\delta$ = 146.9 (s), 137.7 (s), 137.3 (s), 130.4 (s), 127.8 (d), 126.5 (d), 123.4 (d), 118.8 (s), 112.2 (d), 84.4 (s), 36.6 (t), 32.6 (t), 32.4 (t), 29.6 (t), 25.4 (q), 23.3 (t), 14.5 (q) ppm. HR-ESI-FTMS $[M+H]^+$ m/z calcd. 536.3694 for $C_{36}H_{47}BNO_2^+$, found 536.3687.

3,6-Dihexyl-9-[4-(4,4,5,5-tetramethyl-1,3,2-dioxaborolan-2-yl)phenyl]-9*H*-carbazole (**14**). The synthesis of **14** followed the general protocol starting from bromide **13** (2.55 g, 5.2 mmol, 1.0 eq), n-BuLi (2.5 mL, 6.2 mmol, 1.2 eq) and isopropyl pinacol borate (1.16 g, 6.2 mmol, 1.2 eq) in 26 mL of anhydrous THF. **14** was isolated as a white solid (2.09 g, 3.9 mmol, 75%) by passing through a pad of silica (15 g silica gel, light petroleum : DCM). $R_f$ = 0.20 (light petroleum: DCM, 17 : 3). $^1$H NMR (400 MHz, $CD_2Cl_2$): $\delta$ = 8.02 (d, J = 8.5 Hz, 2 H), 7.97 - 7.89 (m, 2 H), 7.61 (d, J = 8.5 Hz, 2 H), 7.39 (d, J = 8.5 Hz, 2 H), 7.25 (dd, J = 8.5, 1.6 Hz, 2 H), 2.81 (t, J = 7.5 Hz, 4 H), 1.81 - 1.67 (m, 4 H), 1.49 - 1.30 (m, 24 H), 0.93 (t, J = 6.9 Hz, 6 H) ppm. $^{13}$C NMR (100 MHz, $CD_2Cl_2$): $\delta$ = 141.4 (s), 139.17 (s), 136.8 (d), 135.4 (s), 127.2 (d), 126.2 (d), 124.2 (s), 120.0 (d), 110.1 (d), 84.6 (s), 36.5 (t), 33.0 (t), 32.5 (t), 29.7 (t), 25.3 (q), 23.3 (t), 14.5 (q) ppm. HR-ESI-FTMS $[M+H]^+$ m/z calcd. 538.3809 for $C_{36}H_{49}BNO_2^+$, found 538.3847.

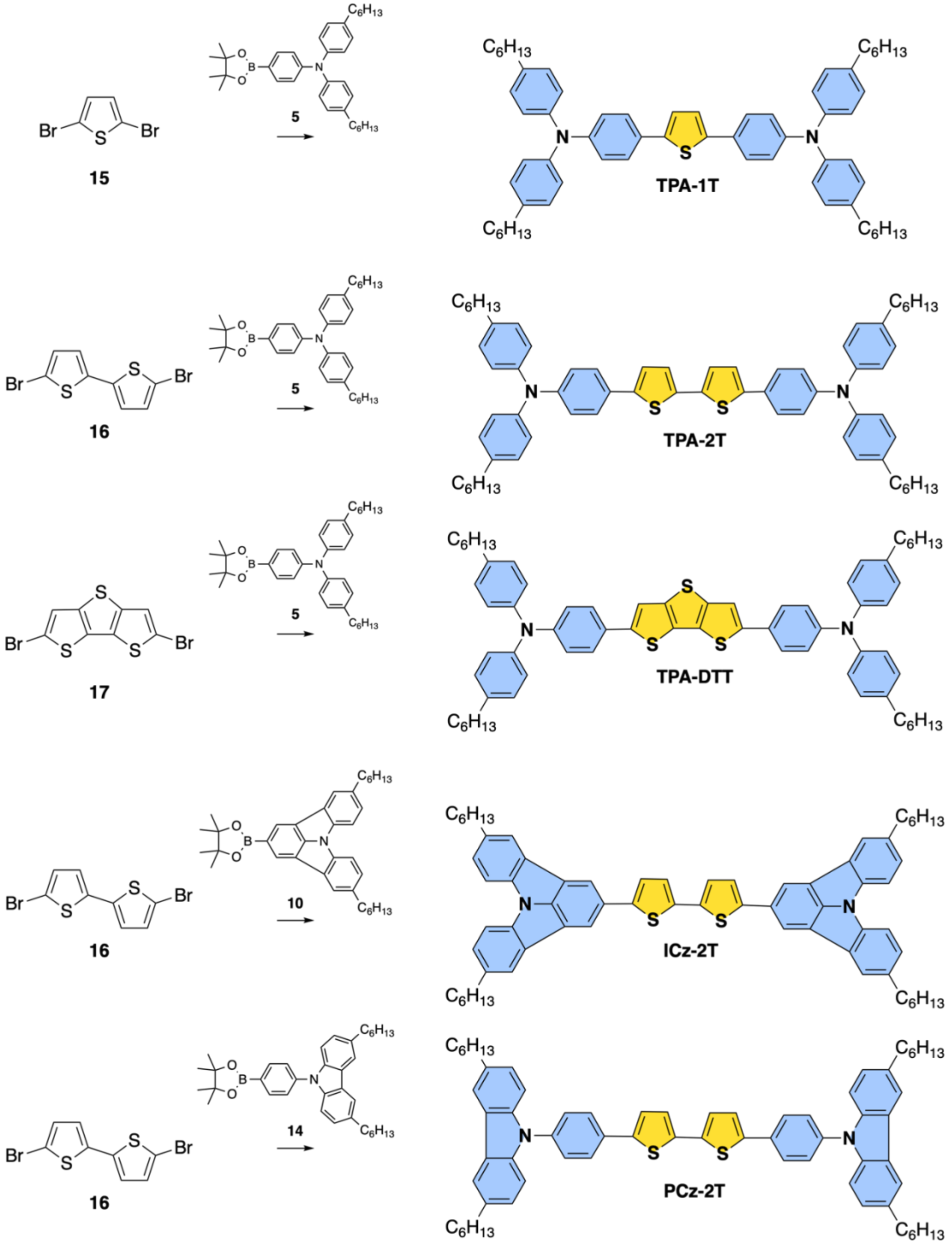


General procedure for the synthesis of **TPA-1T**, **TPA-2T**, **TPA-DTT**, **ICz-2T** and **PCz-2T** according to Marion.[15] To a suspension of the dihalogenide (1 eq), the boronic ester (~ 3 eq) and KOtBu (3 eq) in a mixture of IPA / $H_2O$ (3 : 1) under argon atmosphere (NHC)Pd(allyl)Cl (20 µmol, 2 mol%) was added. The reaction mixture was refluxed until TLC showed full conversion (2 - 4 h). After cooling of the reaction mixture, it was poured onto water and repeatedly extracted with DCM. The combined organic layers were dried over $Na_2SO_4$ and concentrated under reduced pressure.

4,4'-(2,5-Thiophenediyl)bis[*N*,*N*-bis(4-hexylphenyl)benzenamine] (**TPA-1T**). To a suspension of boronic ester **5** (1715 mg, 3.2 mmol 3.2 eq), bromide **15** (242 mg, 1.0 mmol, 1.0 eq) and KOtBu (337 mg, 3.0 mmol, 3.0 eq) in a mixture of 16 mL IPA / $H_2O$ under argon atmosphere (IPr)Pd(allyl)Cl (11 mg, 20 μmol, 2 mol%) was added. The reaction mixture was refluxed until TLC showed full conversion (2 h). After standard workup procedure purification of **TPA-1T** was performed by column chromatography (90 g silica gel, light petroleum : DCM, 9 : 1 ), recrystallization from n-BuOH and column chromatography (90 g silica gel, light petroleum : toluene, 4 : 1). **TPA-1T** could be obtained as a green sticky mass (518 mg, 0.57 mmol, 57%). $^{1}H$ NMR (400 MHz, $CD_2Cl_2$): δ = 7.49 - 7.41 (m, 4 H), 7.17 (s, 2 H), 7.12 - 7.06 (m, 8 H), 7.04 - 6.95 (m, 12 H), 2.57 (t, J = 7.9 Hz, 8 H), 1.65 - 1.57 (m, 8 H), 1.40 - 1.32 (m, 24 H), 0.90 (t, J = 6.7 Hz, 12 H) ppm. $^{13}C$ NMR (100 MHz, $CD_2Cl_2$): δ = 148.2 (s), 145.7 (s), 143.1 (s), 138.7 (s), 129.8 (d), 128.0 (s), 126.6 (d), 125.2 (d), 123.4 (d), 122.9 (d), 35.9 (t), 32.3 (t), 32.1 (t), 29.6 (t), 23.2 (t), 14.4 (q) ppm. HR-ESI-FTMS $[M+H]^+$ m/z calcd. 907.5958 for $C_{64}H_{79}N_2S^+$, found 907.5931.

4,4'-(2,2'-Bithiophene-5,5'-diyl)bis[*N*,*N*-bis(4-hexylphenyl)benzenamine] (**TPA-2T**). To a suspension of boronic ester **5** (1680 mg, 3.1 mmol 3.1 eq), bithiophene **16** (324 mg, 1.0 mmol, 1.0 eq) and KOtBu (337 mg, 3.0 mmol, 3.0 eq) in a mixture 20 mL of IPA / $H_2O$ in argon atmosphere (IPr)Pd(allyl)Cl (11 mg, 20 μmol, 2 mol%) was added. The reaction mixture was refluxed until TLC showed full conversion (1.5 h). After standard workup procedure purification of **TPA-2T** was performed by column chromatography (90 g silica gel, light petroleum : DCM) followed by recrystallization from n-BuOH yielding **TPA-2T** as a red sticky mass (809 mg, 0.82 mmol, 82%). $^{1}H$ NMR (400 MHz, $CD_2Cl_2$): δ = 7.52 - 7.33 (m, 4 H), 7.19 - 7.06 (m, 12 H), 7.05 - 6.93 (m, 12 H), 2.58 (t, J = 7.7 Hz, 8 H), 1.63 - 1.58 (m, 8 H), 1.41 - 1.34 (m, 24 H), 0.92 - 0.89 (m, 12 H) ppm. $^{13}C$ NMR (100 MHz, $CD_2Cl_2$): δ = 148.5 (s), 145.6 (s), 143.6 (s), 138.9 (s), 136.2 (s), 129.8 (d), 127.5 (s), 126.7 (d), 125.4 (d), 124.8 (d), 123.2 (d), 122.7 (d), 35.9 (t), 32.2 (t), 32.1 (t), 29.7 (t), 23.2 (t), 14.5 (q) ppm. HR-ESI-FTMS $[M+H]^+$ m/z calcd. 989.5836 for $C_{68}H_{81}N_2S_2^+$, found 989.5795.

4,4'-(Dithieno[3,2-*b*:2',3'-*d*]thiophene-2,6-diyl)bis[*N*,*N*-bis(4-hexylphenyl)benzenamine] (**TPA-DTT**). Boronic ester **5** (1619 mg, 3.0 mmol, 3 eq), dithienothiophene **17** (354 mg, 1.0 mmol, 1 eq), KOtBu (337 mg, 3.0 mmol, 3 eq) and (IPr)Pd(allyl)Cl (11 mg, 20 μmol, 2 mol%) were refluxed in a mixture of 20 mL IPA / $H_2O$ for 2 h. Purification was performed by column chromatography (90 g silica gel, light petroleum : toluene, 9 → 14%) yielding **TPA-DTT** as a yellow glass (590 mg, 0.58 mmol, 58%). $^{1}H$ NMR (400 MHz, $CD_2Cl_2$): δ = 7.50 - 7.43 (m, 4 H), 7.40 (s, 2 H), 7.15 - 7.08 (m, 8 H), 7.04 - 6.97 (m, 12 H), 2.58 (t, J = 7.7 Hz, 8 H), 1.65 - 1.58 (m, 8 H), 1.40 - 1.30 (m, 24 H), 0.90 (t, J = 6.9 Hz, 12 H) ppm. $^{13}C$ NMR (100 MHz, $CD_2Cl_2$): δ = 148.7 (s), 145.6 (s), 145.5 (s), 142.1 (s), 139.0 (s), 129.9 (s), 129.8 (d), 127.8 (s), 126.8 (d), 125.4 (d), 122.6 (d), 115.8 (d), 35.9 (t), 32.3 (t), 32.1 (t), 29.6 (t), 23.2 (t), 14.5 (q) ppm. HR-ESI-FTMS $[M+H]^+$ m/z calcd. 1019.5400 for $C_{68}H_{79}N_2S_3^+$, found 1019.5360.

9,9'-(2,2'-Bithiophene-5,5'-diyldi-4,1-phenylene)bis[3,6-dihexyl-9*H*-carbazole] (**ICz-2T**). Boronic ester **10** (1619 mg, 3.0 mmol, 3 eq), bithiophene **16** (324 mg, 1.0 mmol, 1 eq), KOtBu (337 mg, 3.0 mmol, 3 eq) and (IPr)Pd(allyl)Cl (11 mg, 20 □mol, 2 mol%) were refluxed in a mixture of 20 mL IPA / $H_2O$ for 2 h. Purification was performed by column chromatography (90 g silica gel, light petroleum : DCM, 9 : 1) followed by recrystallization from n-heptane yielding **ICz-2T** as a yellow solid (827 mg, 0.84 mmol, 84%). $^{1}$H NMR (400 MHz, $CD_2Cl_2$): $\delta$ = 7.98 - 7.90 (m, 4 H), 7.88 - 7.79 (m, 4 H), 7.65 - 7-56 (m, 4 H), 7.40 - 7.38 (m, 6 H), 7.29 - 7.24 (m, 6 H), 2.80 (t, J = 7.8 Hz, 8 H), 1.76 - 1.69 (m, 8 H), 1.41 - 1.34 (m, 24 H), 0.91 (t, J = 6.9 Hz, 12 H) ppm. $^{13}$C NMR (100 MHz, $CD_2Cl_2$): $\delta$ = 142.9 (s), 139.8 (s), 138.1 (s), 137.4 (s), 135.4 (s), 133.1 (s), 127.6 (d), 127.4 (d), 127.2 (d), 125.4 (d), 124.9 (d), 124.1 (s), 120.1 (d), 110.0 (d), 36.5 (t), 32.9 (t), 32.4 (t), 29.7 (t), 23.3 (t), 14.5 (q) ppm. HR-ESI-FTMS $[M+H]^+$ m/z calcd. 985.5523 for $C_{68}H_{77}N_2S_2^+$, found 985.5490.

2,2'-(2,2'-Bithiophene-5,5'-diyl)bis[5,11-dihexylindolo[3,2,1-*jk*]carbazole] (**PCz-2T**). Boronic ester **14** (1607 mg, 3.0 mmol, 3 eq), bithiophene **16** (324 mg, 1.0 mmol, 1 eq), KOtBu (337 mg, 3.0 mmol, 3 eq) and (IPr)Pd(allyl)Cl (11 mg, 20 µmol, 2 mol%) were refluxed in a mixture of 20 mL IPA / $H_2O$ for 4 h. **PCz-2T** was isolated as an orange solid (642 mg, 0.65 mmol, 65%) after column chromatography (90 g silica gel, light petroleum : DCM, 10% $\rightarrow$ 100%) and subsequent digesting in acetone. $^{1}$H NMR (400 MHz, $CD_2Cl_2$): $\delta$ = 8.31 (s, 4 H), 8.00 (s, 4 H), 7.80 (d, J = 8.2 Hz, 4 H), 7.44 - 7.41 (m, 6 H), 7.32 (d, J = 3.5 Hz, 2 H), 2.83 (t, J = 7.8 Hz, 8 H), 1.76 (quin, J = 7.7 Hz, 8 H), 1.45 - 1.33 (m, 24 H), 0.92 (t, J = 7.8 Hz, 12 H) ppm. $^{13}$C NMR (100 MHz, $CD_2Cl_2$): $\delta$ = 145.9 (s), 144.8 (s), 138.1 (s), 137.4 (s), 136.6 (s), 130.2 (s), 130.1 (s), 128.2 (d), 124.9 (d), 123.9 (d), 123.5 (d), 119.3 (s), 118.0 (d), 112.4 (s), 36.6 (t), 32.7 (t), 32.4 (t), 29.6 (t), 23.2 (t), 14.5 (q) ppm. HR-ESI-FTMS $[M+H]^+$ m/z calcd. 981.5210 for $C_{68}H_{73}N_2S_2^+$, found 981.5216.

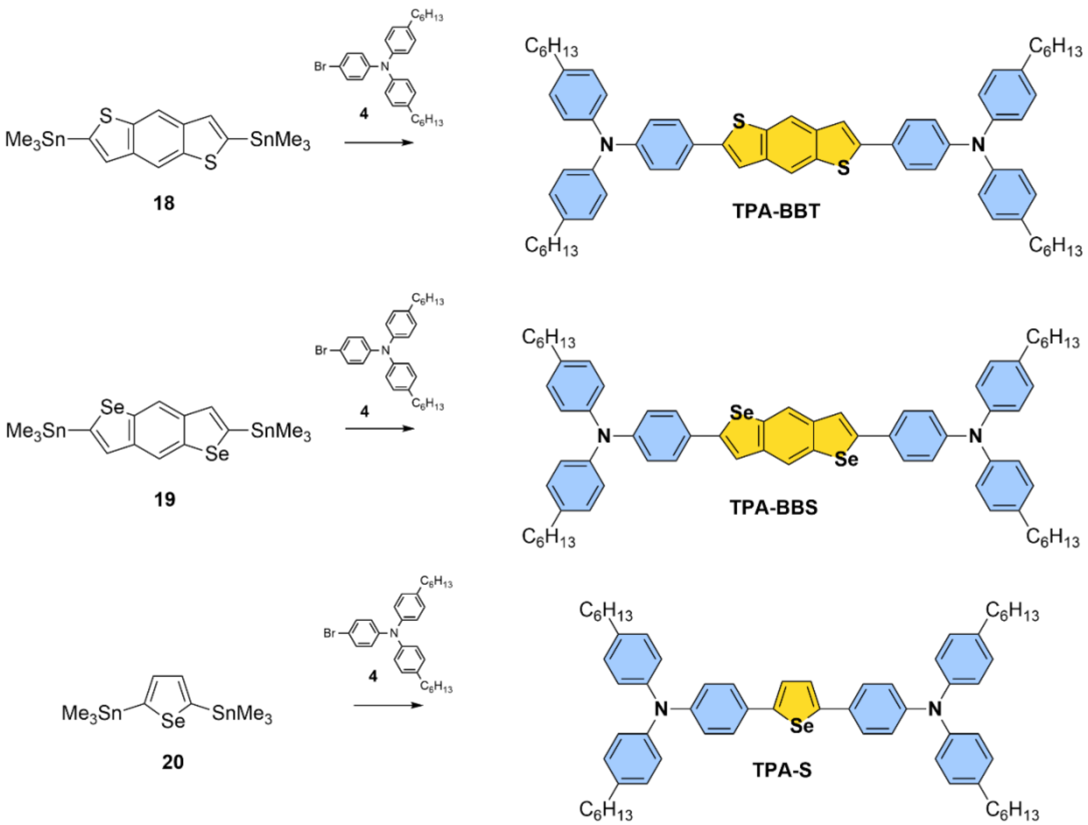


General procedure for the synthesis of **TPA-BBT**, **TPA-BBS** and **TPA-S** according to Haid.[6] Organodistannane (1.0 eq), bromide (2.1 eq) and $Pd(PPh_3)_4$ (5 mol%) were dissolved in degassed anhydrous DMF under argon atmosphere. The solution was degassed again before it was stirred at 80 °C until TLC showed full conversion. After cooling of the solution, it was poured onto water and repeatedly extracted with DCM. The combined organic layers were dried over $Na_2SO_4$ and concentrated *in vacuo*. Purification was performed as stated in the detailed descriptions.

4,4'-(Benzo[1,2-*b*:4,5-*b*']dithiophene-2,6-diyl)bis[*N*,*N*-bis(4-hexylphenyl)benzenamine] (**TPA-BBT**). Bromide **4** (1034 mg, 2.1 mmol, 2.1 eq), organodistannane **18** (516 mg, 1.0 mmol, 1 eq) and $Pd(PPh_3)_4$ (58 mg, 50 µmol, 5 mol%) were heated to 80 °C in anhydrous degassed DMF (25 mL) under argon atmosphere for 44 h. Purification was performed by column chromatography (110 g silica gel, light petroleum : toluene, 9 → 22%, 90 g Silica gel, light petroleum : DCM, ~10%, 489 mg, 48%) followed by solving the product in light petroleum yielding **TPA-BBT** as an orange glass (405 mg, 0.58 mmol, 40%). $^1$H NMR (400 MHz, $CD_2Cl_2$): δ = 8.09 (s, 2 H), 7.58 - 7.50 (m, 4 H), 7.43 (s, 2 H), 7.16 – 7.07 (m, 8 H), 7.06 - 6.96 (m, 12 H), 2.58 (t, J = 7.7 Hz, 8 H), 1.64 - 1.58 (m, 8 H), 1.43 - 1.34 (m, 24 H), 0.91 (t, J = 6.7 Hz, 12 H) ppm. $^{13}$C NMR (100 MHz, $CD_2Cl_2$): δ = 149.2 (s), 145.5 (s), 144.8 (s), 139.2 (s), 139.1 (s), 137.3 (s), 129.9 (d), 127.5 (d), 127.4 (s), 125.6 (d), 122.3 (d), 117.5 (d), 116.5 (d), 35.9 (t), 32.3 (t), 32.1 (t), 29.7 (t), 23.2 (t), 14.5 (q) ppm. HR-ESI-FTMS $[M+H]^+$ m/z calcd. 1013.5836 for $C_{70}H_{81}N_2S_2^+$, found 1013.5796.

4,4'-(Benzo[1,2-*b*:4,5-*b'*]diselenophene-2,6-diyl)bis[*N*,*N*-bis(4-hexylphenyl)benzenamine] (**TPA-BBS**). Bromide **4** (1034 mg, 2.1 mmol, 2.1 eq), organodistannane **19** (610 mg, 1.0 mmol, 1 eq) and $Pd(PPh_3)_4$ (58 mg, 50 µmol, 5 mol%) were heated to 90 °C in 25 mL anhydrous degassed DMF under argon atmosphere for 54 h.

**TPA-BBS** was isolated as a yellow glass (364 mg, 0.33 mmol, 33%) after column chromatography (110 g silica gel, light petroleum : toluene, 7 → 15%). $^1$H NMR (400 MHz, $CD_2Cl_2$): δ = 8.11 (s, 2 H), 7.57 (s, 2 H), 7.49 - 7.40 (m, 4 H), 7.15 - 7.06 (m, 8 H), 7.06 - 6.93 (m, 12 H), 2.59 (t, J = 7.6 Hz, 8 H), 1.64 - 1.59 (m, 8 H), 1.39 - 1.35 (m, 24 H), 0.92 (t, J = 6.9 Hz, 12 H) ppm. $^{13}$C NMR (100 MHz, $CD_2Cl_2$): δ = 149.1 (s), 147.7 (s), 145.5 (s), 141.6 (s), 139.1 (s), 137.8 (s), 129.9 (d), 129.3 (s), 127.9 (d), 125.6 (d), 122.3 (d), 121.7 (d), 121.0 (d), 35.9 (t), 32.3 (t), 32.1 (t), 29.7 (t), 23.2 (t), 14.5 (q) ppm. HR-ESI-FTMS $[M+H]^+$ m/z calcd. 1109.4725 for $C_{70}H_{81}N_2Se_2^+$, found 1109.4704.

4,4'-(2,5-Selenophenediyl)bis[*N*,*N*-bis(4-hexylphenyl)benzenamine] (**TPA-S**). Bromide **4** (940 mg, 1.91 mmol, 2.2 eq), organodistannane **20** (400 mg, 0.88 mmol, 1 eq) and $Pd(PPh_3)_4$ (51 mg, 44 µmol, 5 mol%) were heated to 80 °C in 20 mL anhydrous degassed DMF under argon atmosphere for 4 h. **TPA-S** was obtained as a green glass (460 mg, 0.48 mmol, 55%) after column chromatography (90 g silica gel, light petroleum : DCM, 10%; 90 g silica gel, light petroleum : toluene, 9 → 14%). $^1$H NMR (400 MHz, $CD_2Cl_2$): δ = 7.43 - 7.35 (m, 4 H), 7.32 (s, 2 H), 7.14 – 7.07 (m, 8 H), 7.04 - 6.99 (m, 8 H), 6.99 – 6.93 (m, 4 H), 2.57 (t, J = 7.8 Hz, 8 H), 1.65 - 1.57 (m, 8 H), 1.40 - 1.28 (m, 24 H), 0.90 (t, J = 7.0 Hz, 12 H) ppm. $^{13}$C NMR (100 MHz, $CD_2Cl_2$): δ = 149.0 (s), 148.4 (s), 145.6 (s), 138.8 (s), 130.0 (s), 129.8 (d), 127.0 (d), 125.5 (d), 125.3 (d), 122.8 (d), 35.9 (t), 32.3 (t), 32.1 (t), 29.6 (t), 23.2 (t), 14.5 (q) ppm. HR-ESI-FTMS $[M+H]^+$ m/z calcd. 955.5403 for $C_{64}H_{79}N_2Se^+$, found 955.5368.

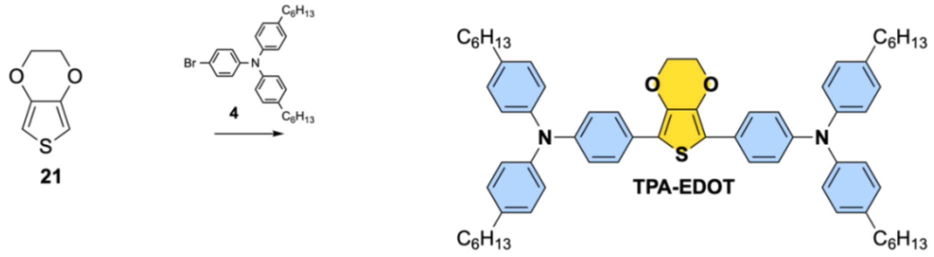


4,4'-(2,3-Dihydrothieno[3,4-*b*]-1,4-dioxine-5,7-diyl)bis[*N*,*N*-bis(4-hexylphenyl)benzenamine] (**TPA-EDOT**). **TPA-EDOT** was synthesized adapting a procedure from literature.[16] To a solution of bromide **4** (1034 mg, 2.1 mmol, 2.1 eq) and $Cs_2CO_3$ (782 mg, 2.4 mmol, 2.4 eq) in anhydrous degassed toluene (5 mL) were added $Pd(OAc)_2$ (11 mg, 50 µmol, 5 mol%), $P(m\text{-}tol)_3$ (30 mg, 0.1 mmol, 0.10 eq) and EDOT **21** (142 mg, 1.0 mmol, 1.0 eq) under argon atmosphere. The reaction mixture was the refluxed for 40 h before it was diluted with DCM and washed with water. The organic phase was dried over $Na_2CO_3$ and concentrated in vacuo. Purification of **TPA-EDOT** was performed by column chromatography (90 g silica gel, light petroleum : DCM, 15%), recrystallization from n-BuOH and column chromatography (90 g silica gel, light petroleum : toluene, 4 : 1) yielding **TPA-EDOT** as green sticky mass (361 mg, 0.37 mmol, 37%). $^1$H NMR (400 MHz, $CD_2Cl_2$): δ = 7.57 (bm, 4 H), 7.10 - 7.08 (bm, 8 H), 7.00 - 6.98 (bm, 12 H), 4.29 (bs, 4 H), 2.58 (t, J = 7.5 Hz, 8 H), 1.65 - 1.58 (m, 8 H), 1.41 - 1.28 (m, 24 H), 0.91 (t, J = 6.4 Hz, 12 H) ppm. $^{13}$C NMR (400 MHz, $CD_2Cl_2$): δ = 147.3 (s), 145.8 (s), 138.5 (s), 138.5 (s), 129.8 (d), 127.2 (d), 127.1 (s), 125.1 (d), 123.1 (d), 65.2 (t), 35.9 (t), 32.4 (t), 32.2 (t), 29.7 (t), 23.3 (t), 14.5 (q) ppm. HR-ESI-FTMS $[M+H]^+$ m/z calcd. 965.6013 for $C_{66}H_{81}N_2O_2S^+$, found 965.6157.

## C) NMR Spectra

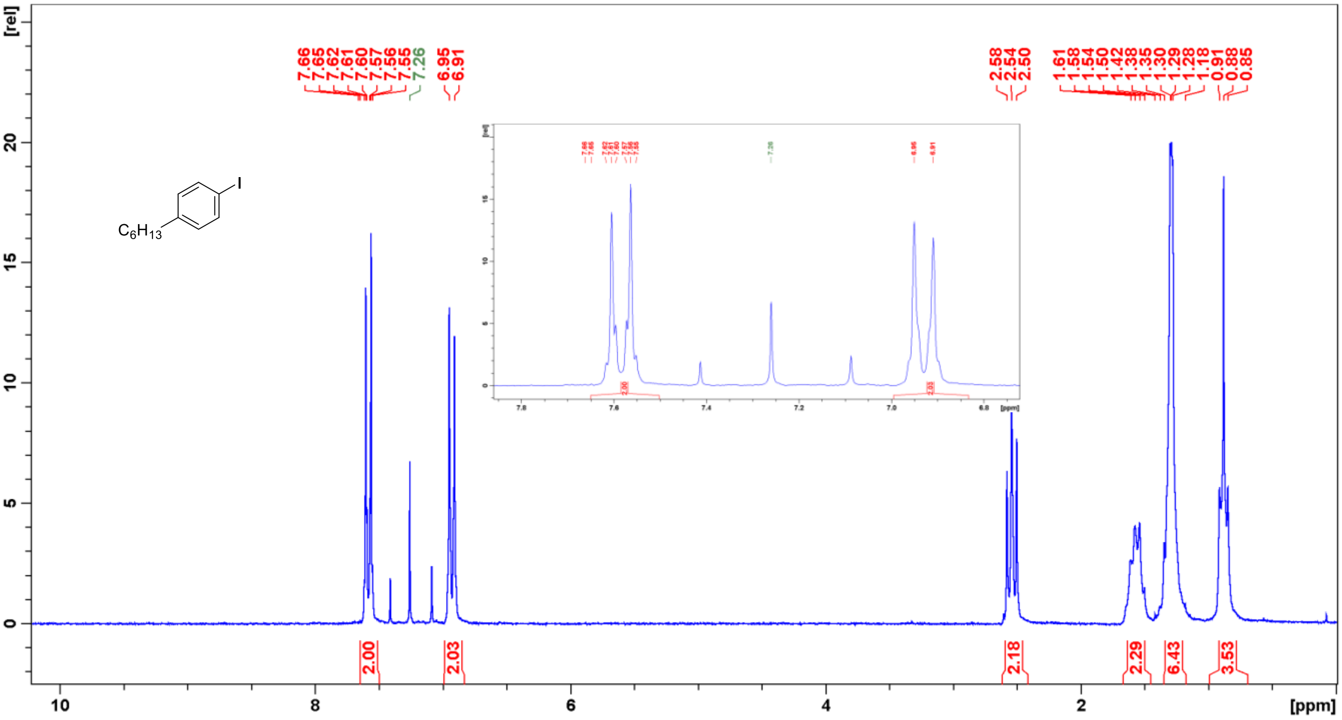


Figure S1. Proton NMR spectrum of compound **2**.

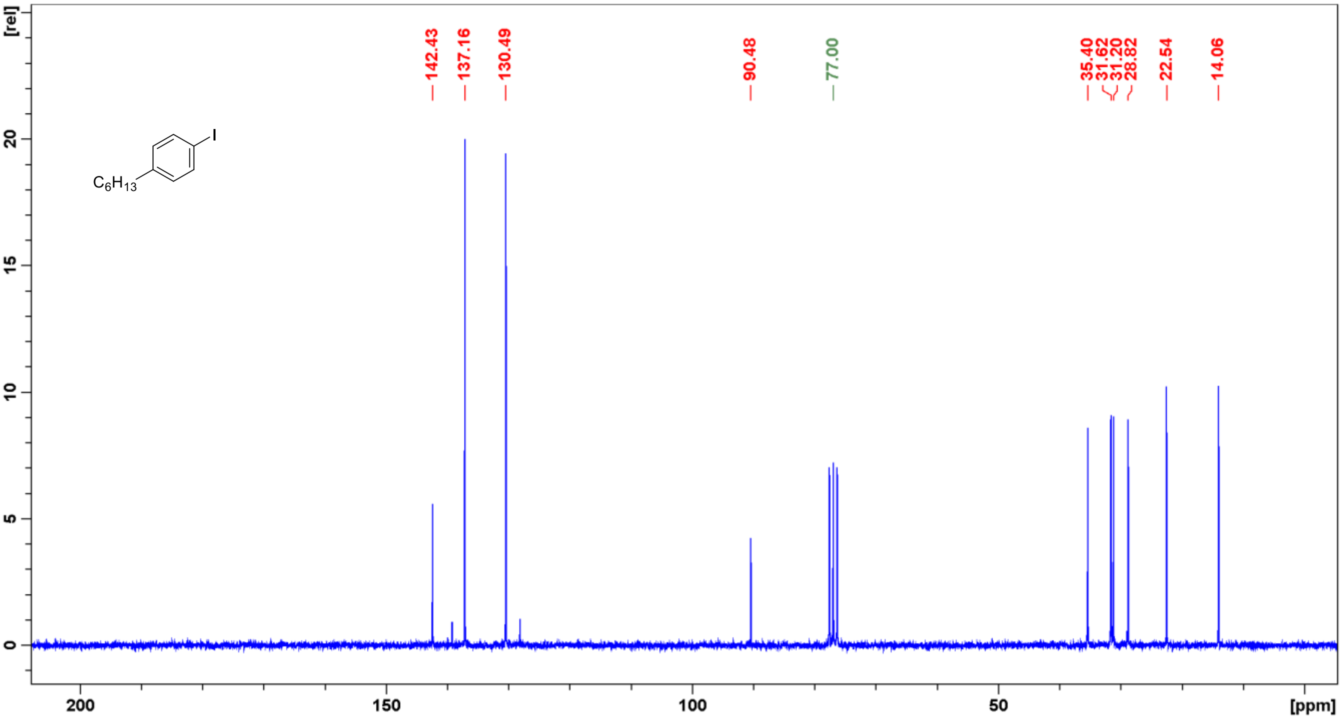


Figure S2. Carbon NMR spectrum of compound **2**.

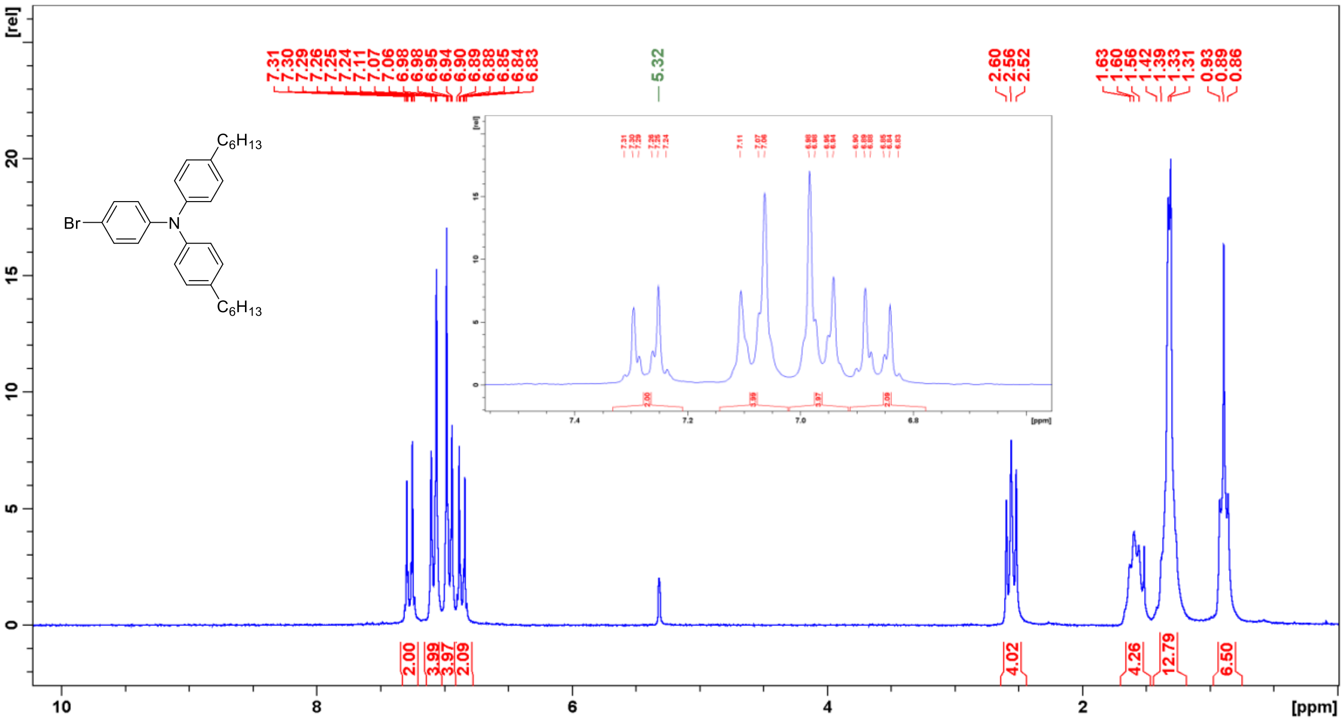

Figure S3. Proton NMR spectrum of compound **4**.

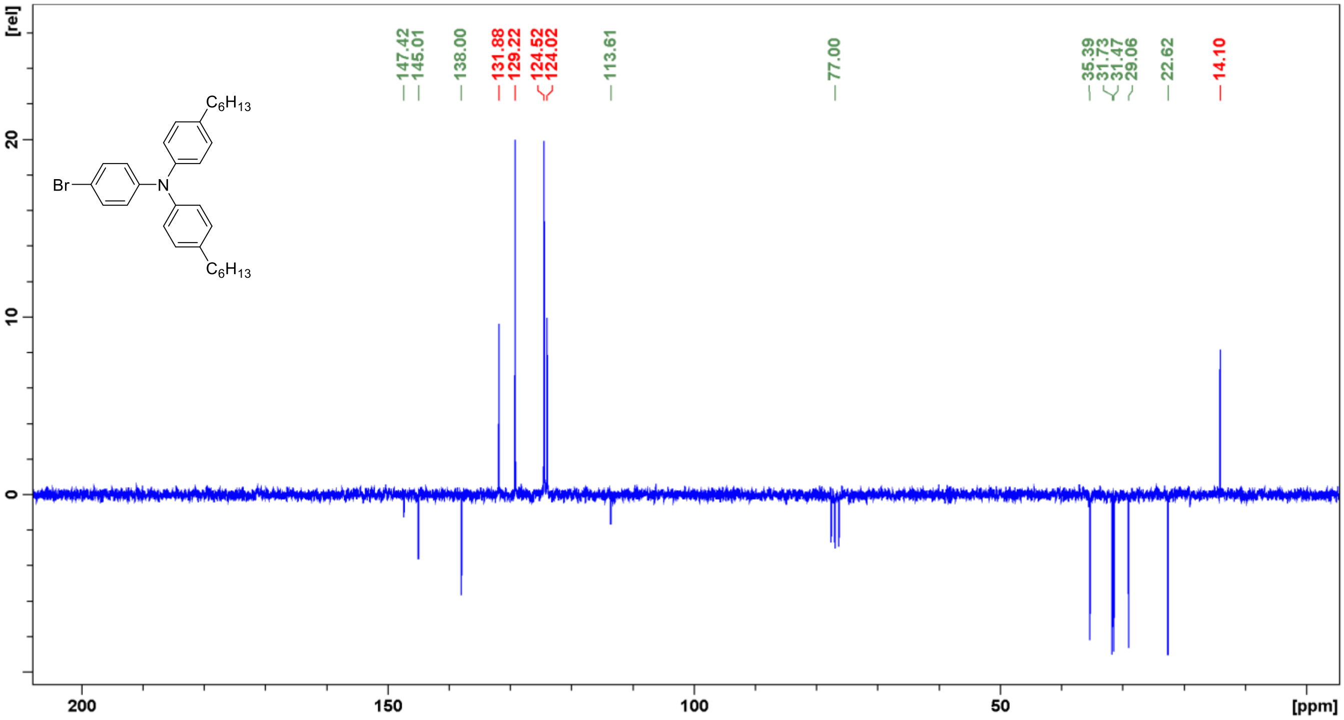

Figure S4. Carbon NMR spectrum of compound **4**.

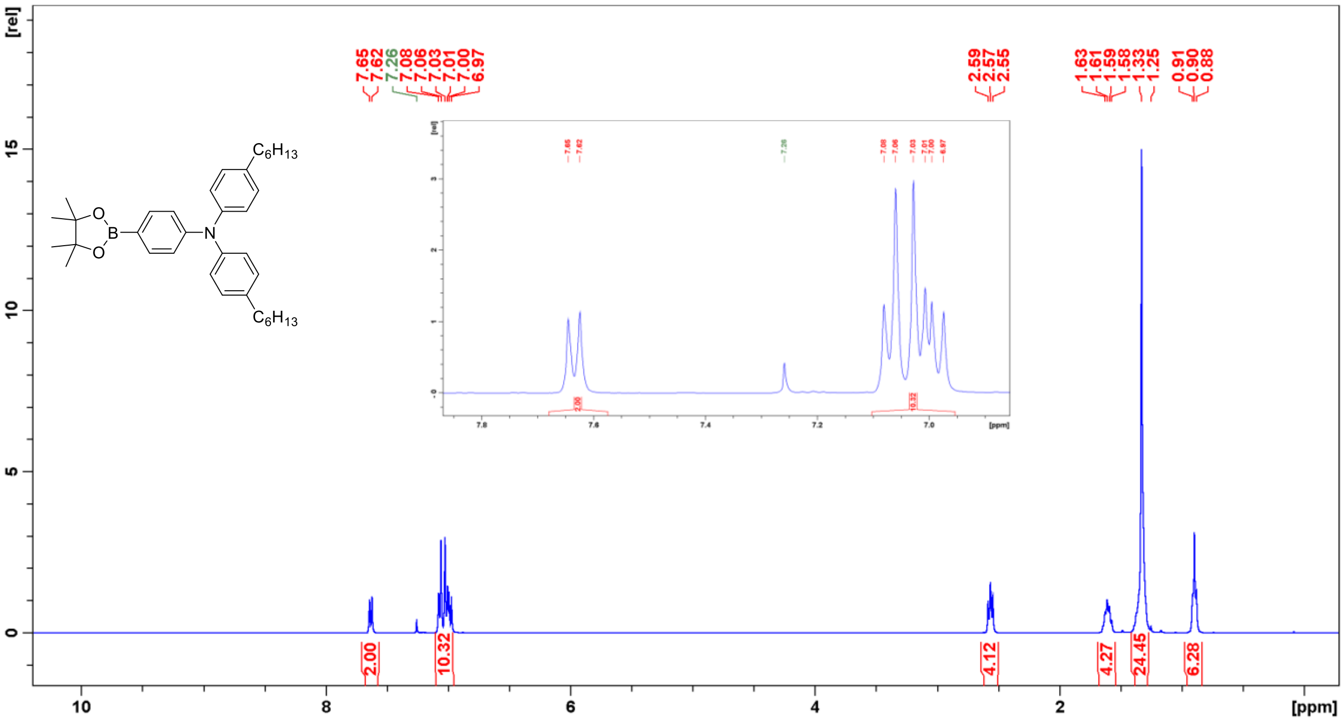

Figure S5. Proton NMR spectrum of compound **5**.

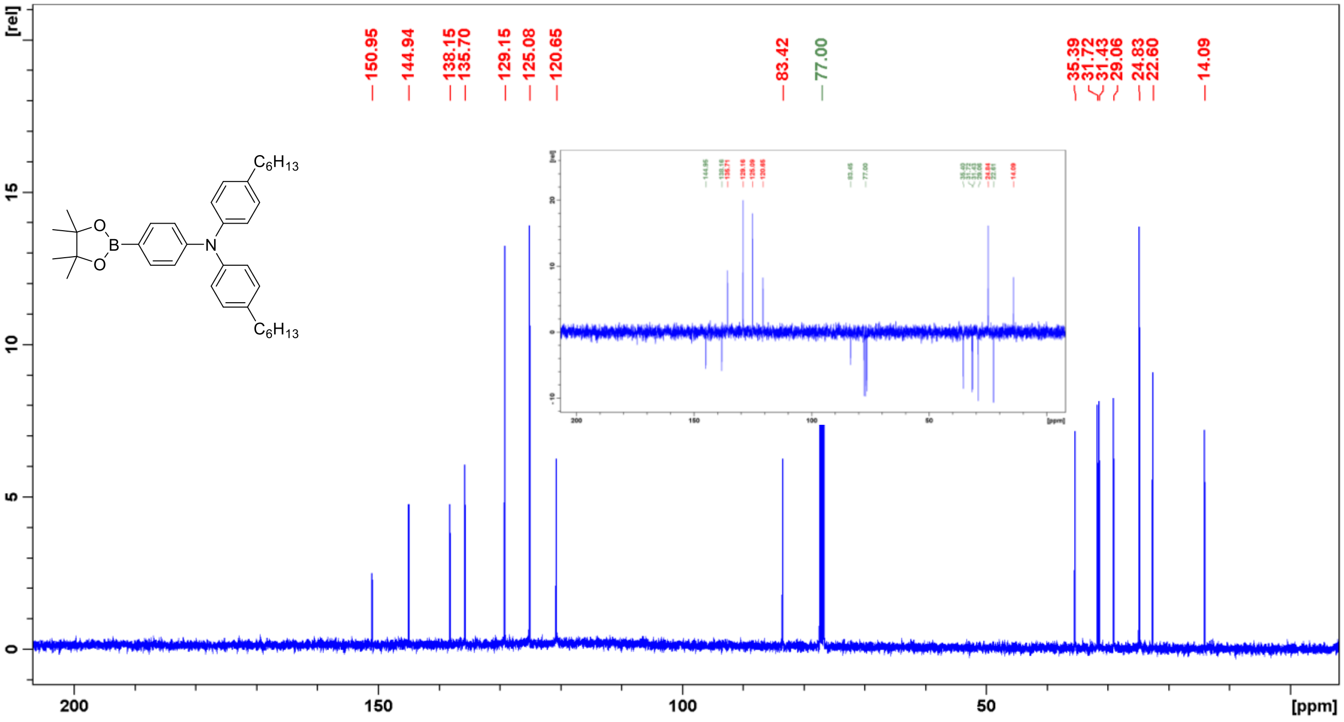

Figure S6. Carbon NMR spectrum of compound **5**.

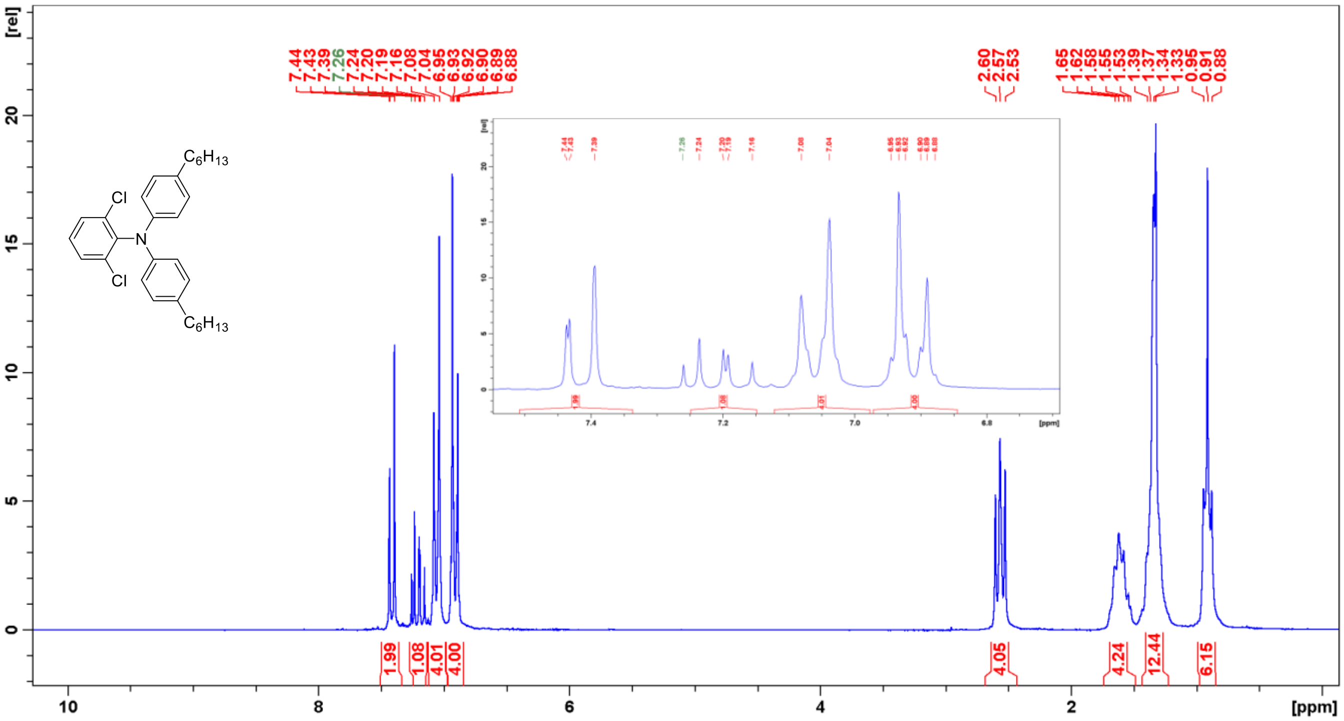

Figure S7. Proton NMR spectrum of compound **7**.

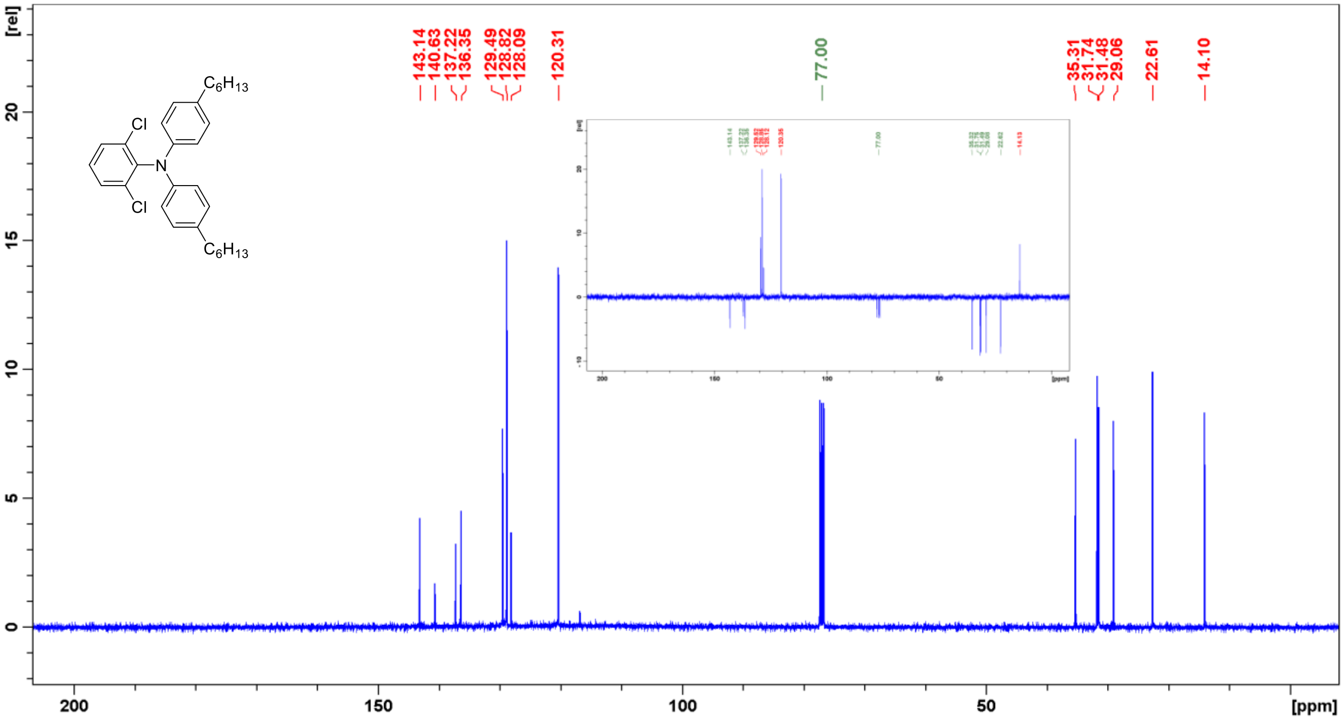

Figure S8. Carbon NMR spectrum of compound **7**.

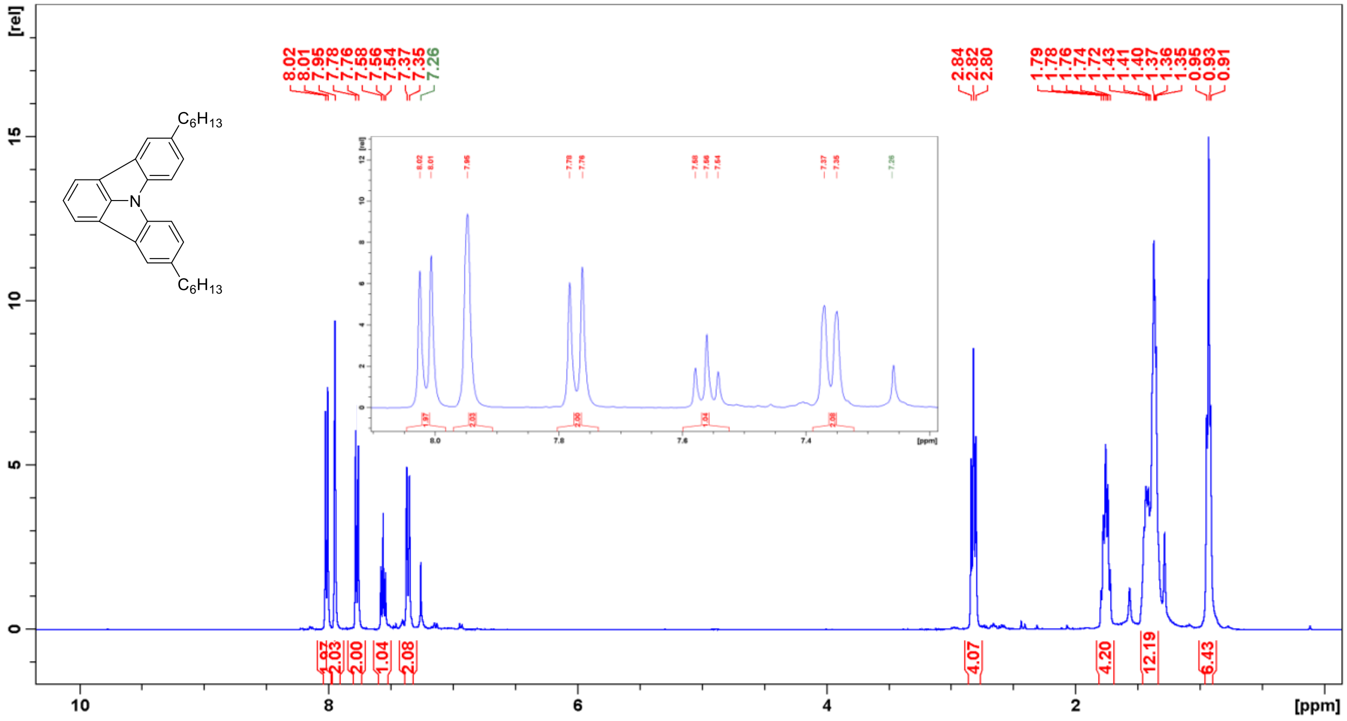

Figure S9. Proton NMR spectrum of compound **8**.

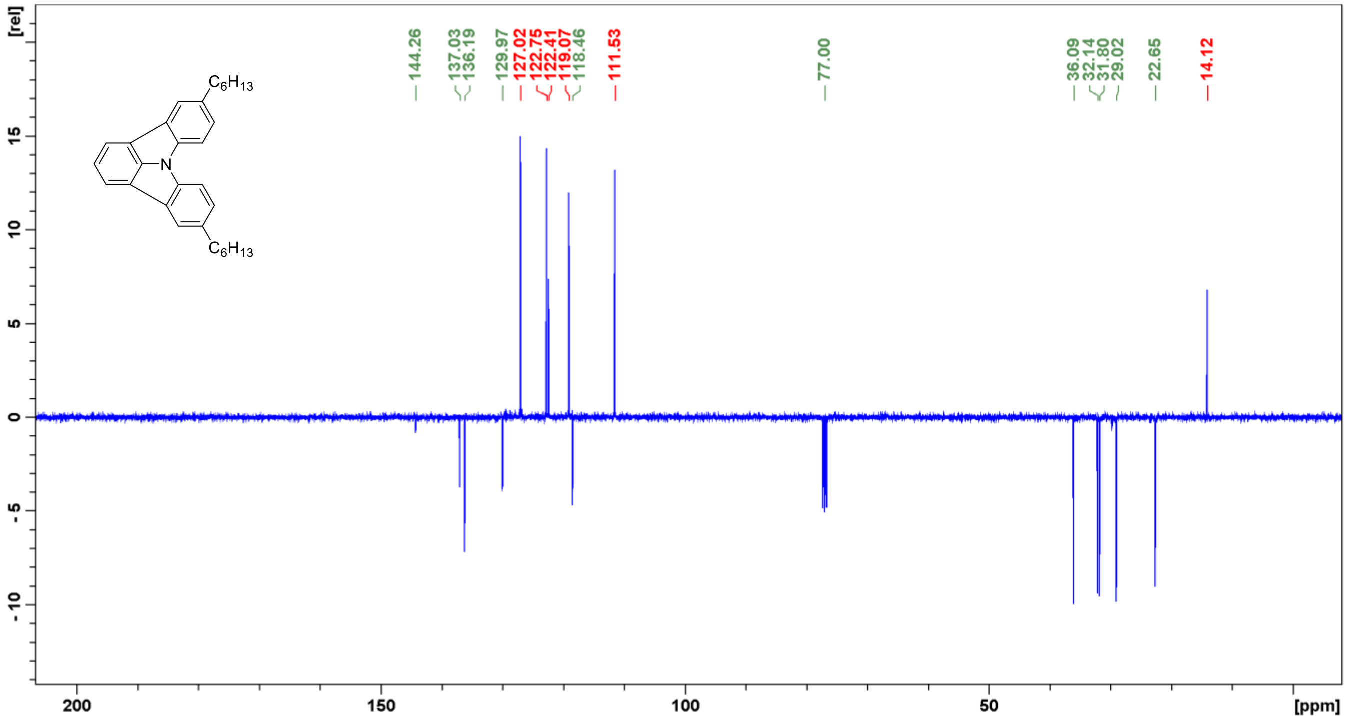

Figure S10. Carbon NMR spectrum of compound **8**.

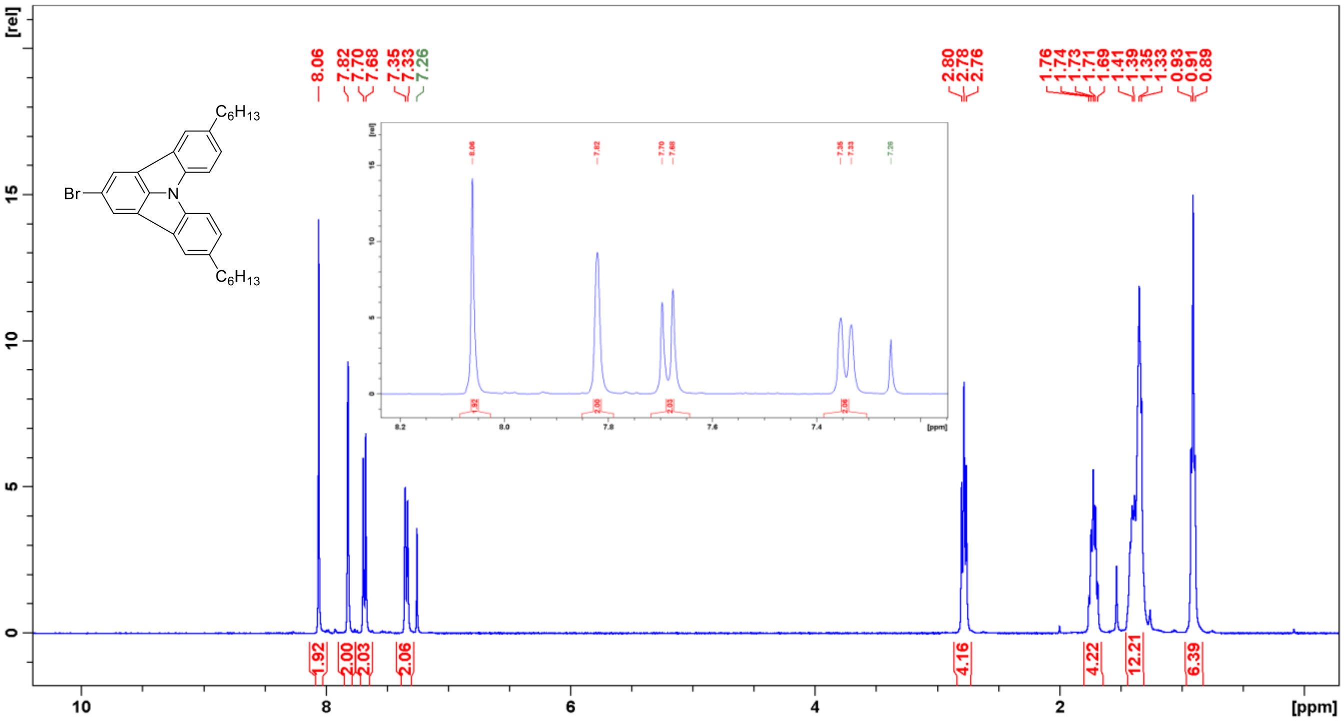


Figure S11. Proton NMR spectrum of compound **9**.

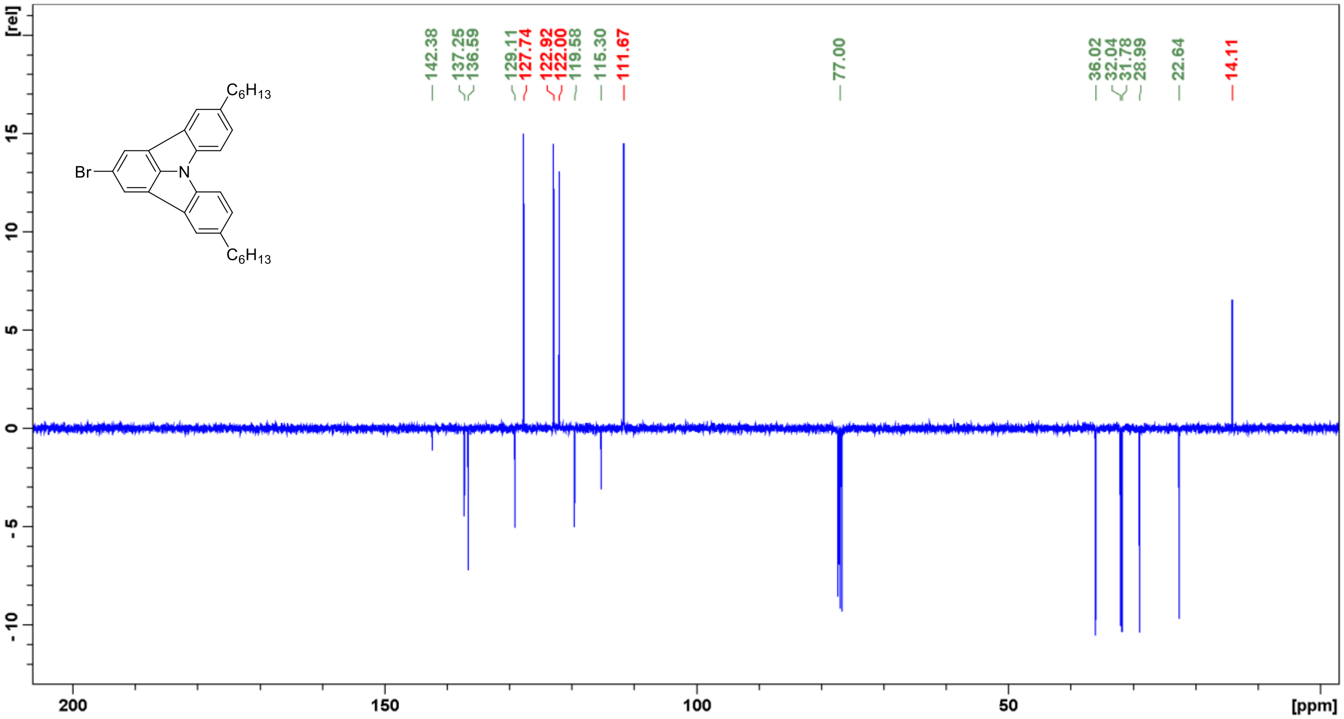


Figure S12. Carbon NMR spectrum of compound **9**.

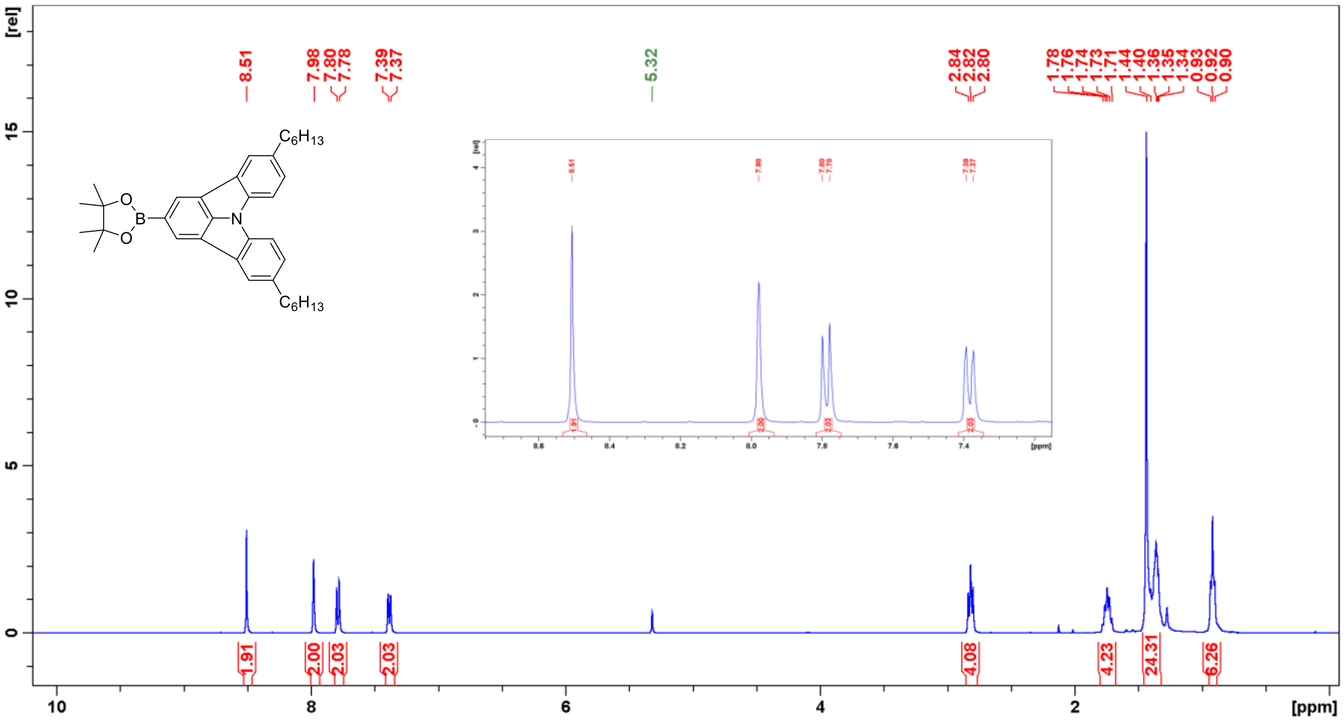


Figure S13. Proton NMR spectrum of compound **10**.

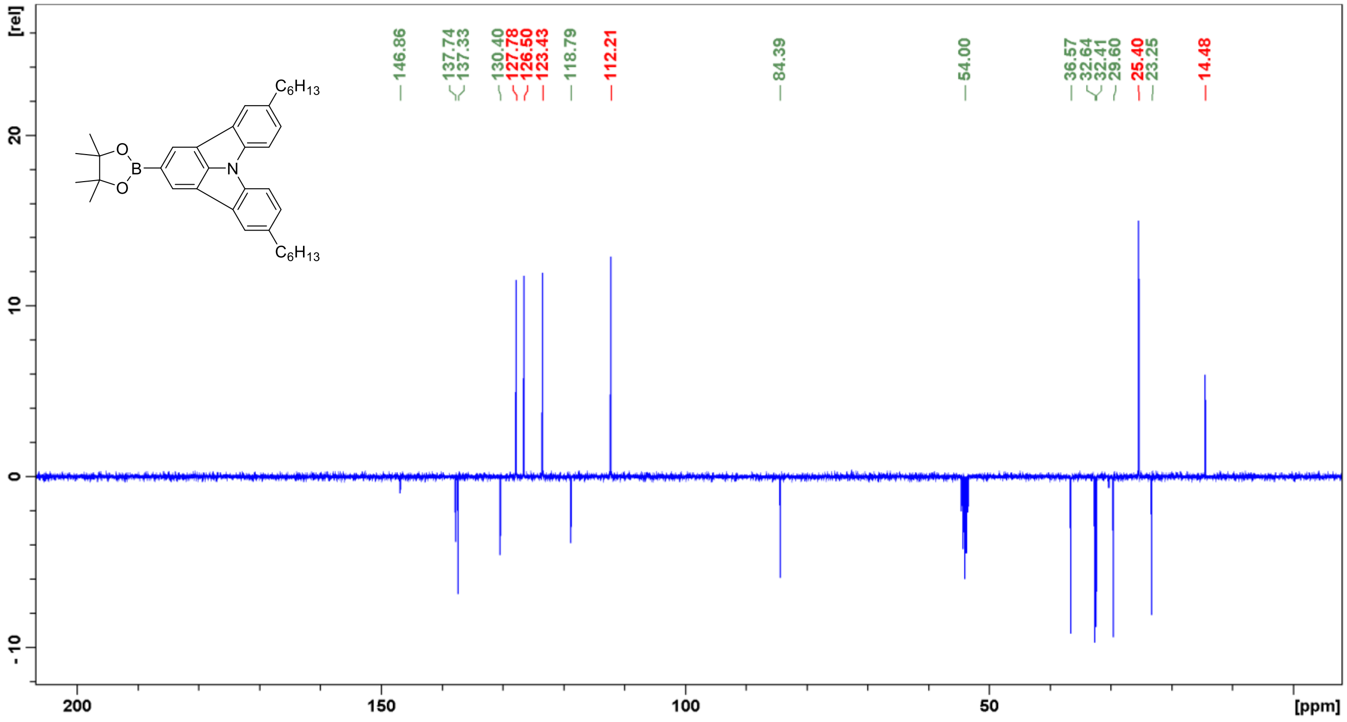


Figure S14. Carbon NMR spectrum of compound **10**.

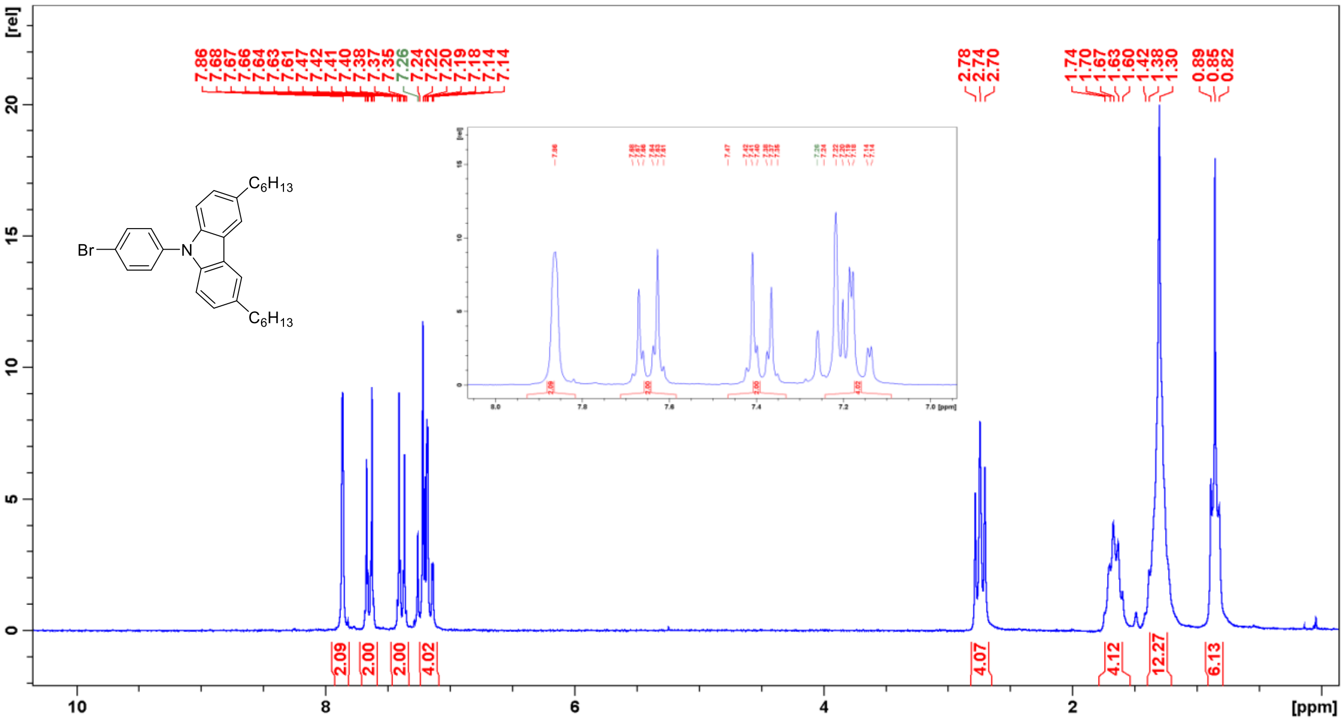


Figure S15. Proton NMR spectrum of compound **13**.

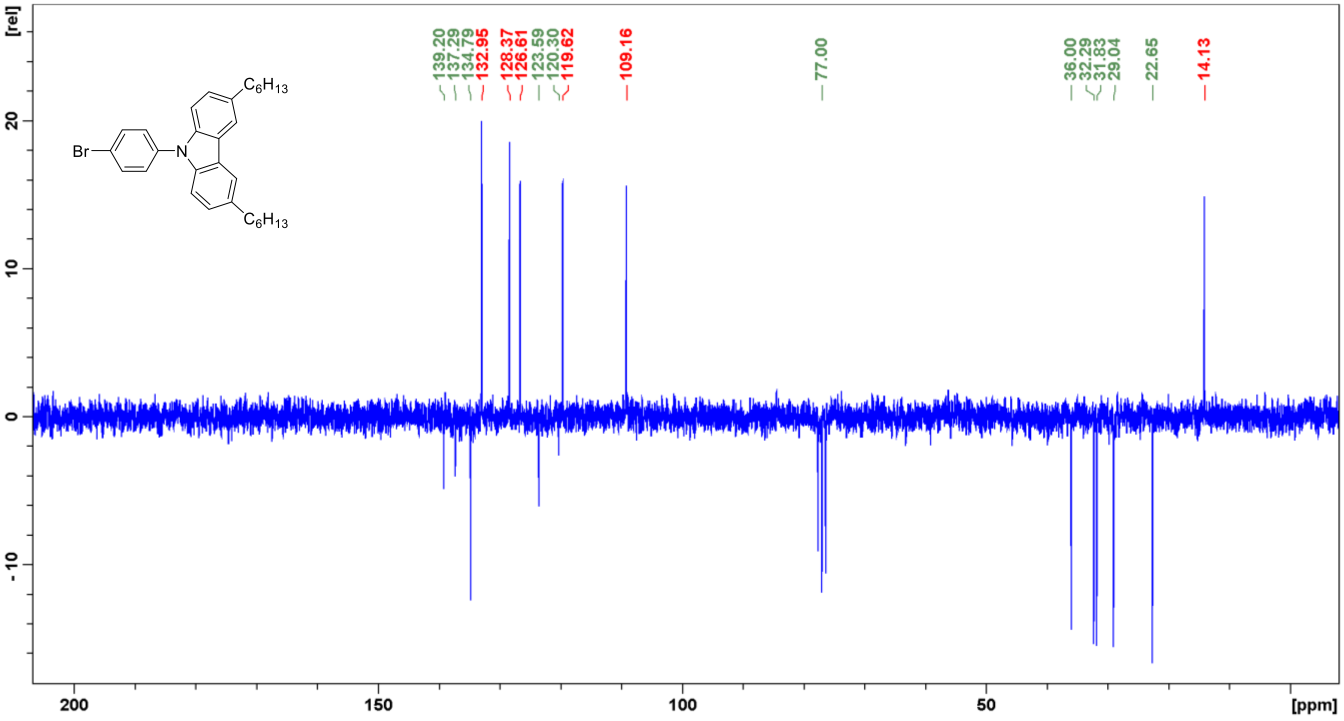


Figure S16. Carbon NMR spectrum of compound **13**.

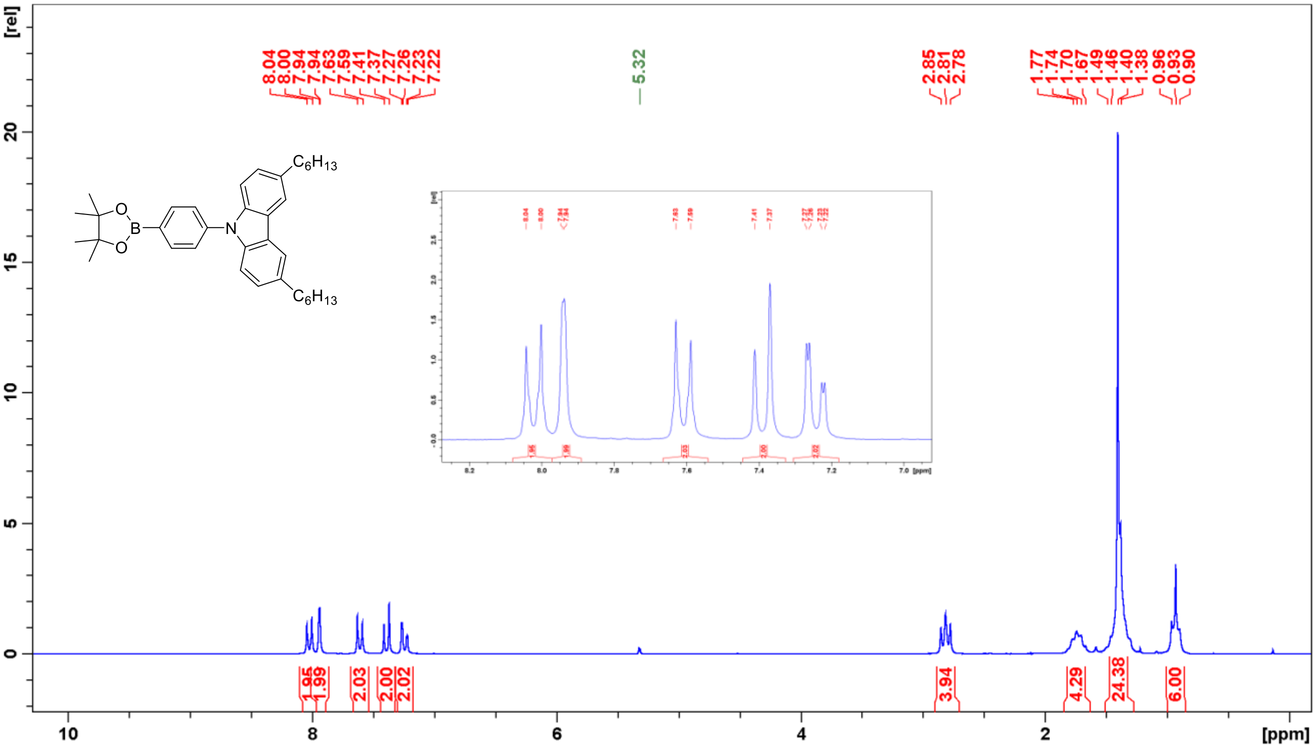

Figure S17. Proton NMR spectrum of compound **14**.

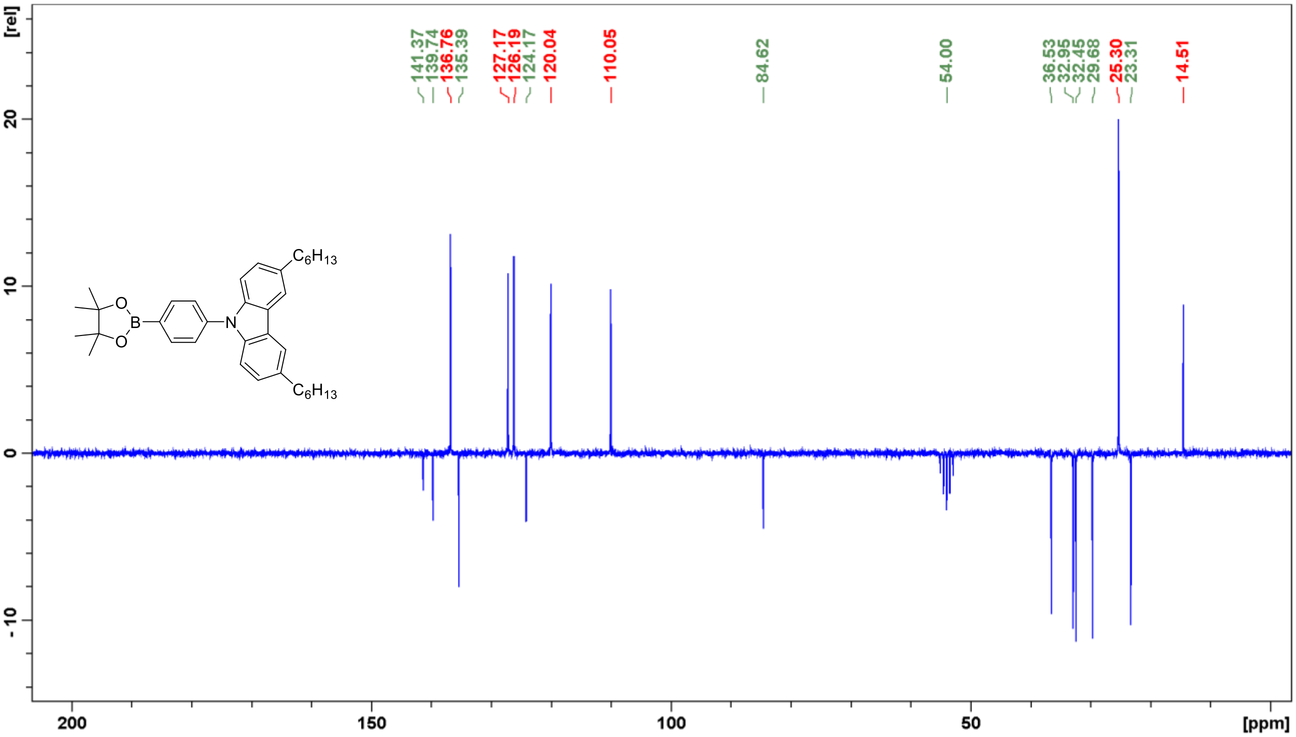

Figure S18. Carbon NMR spectrum of compound **14**.

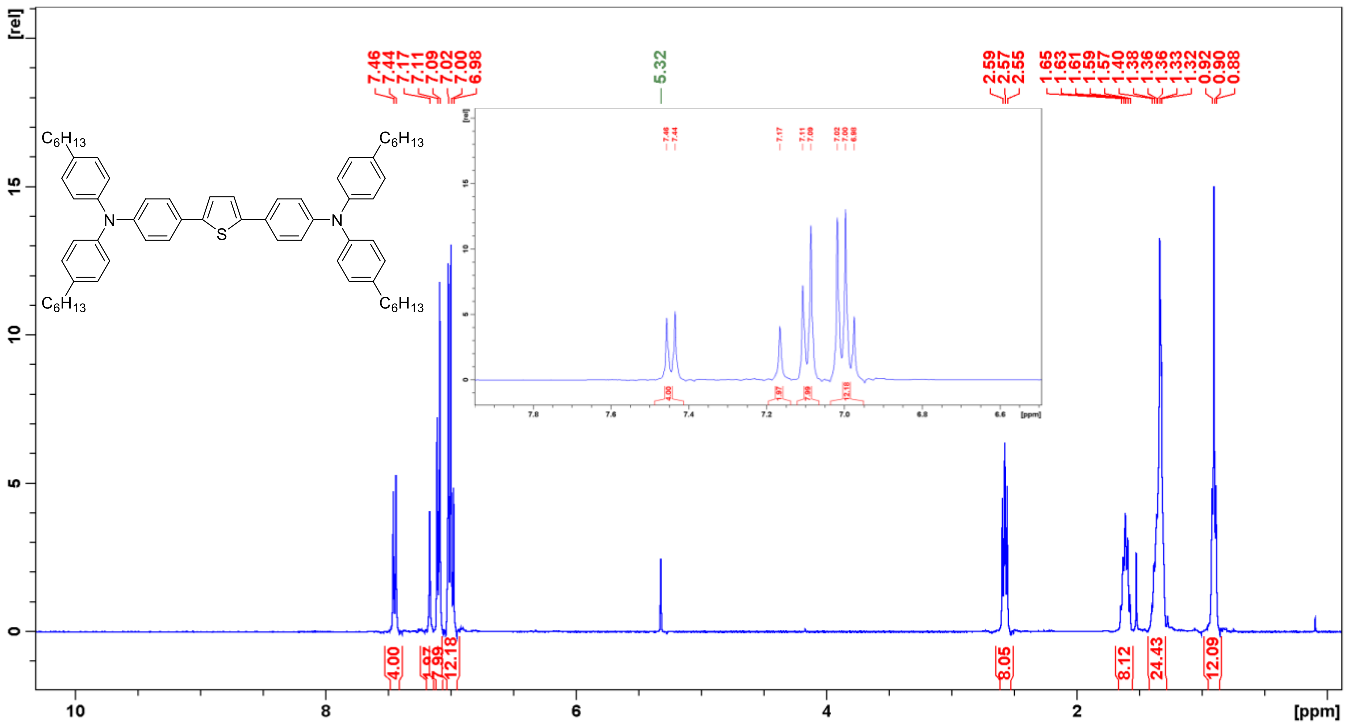


Figure S19. Proton NMR spectrum of compound **TPA-1T**.

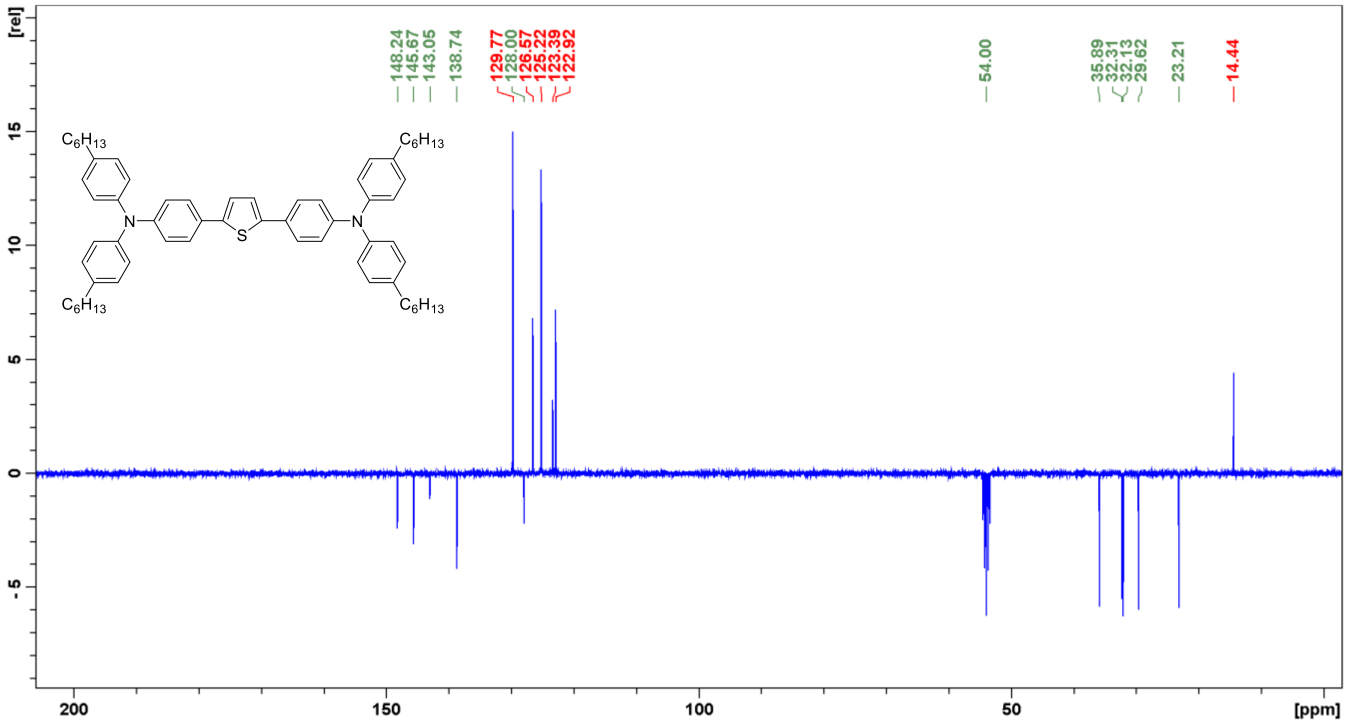


Figure S20. Carbon NMR spectrum of compound **TPA-1T**.

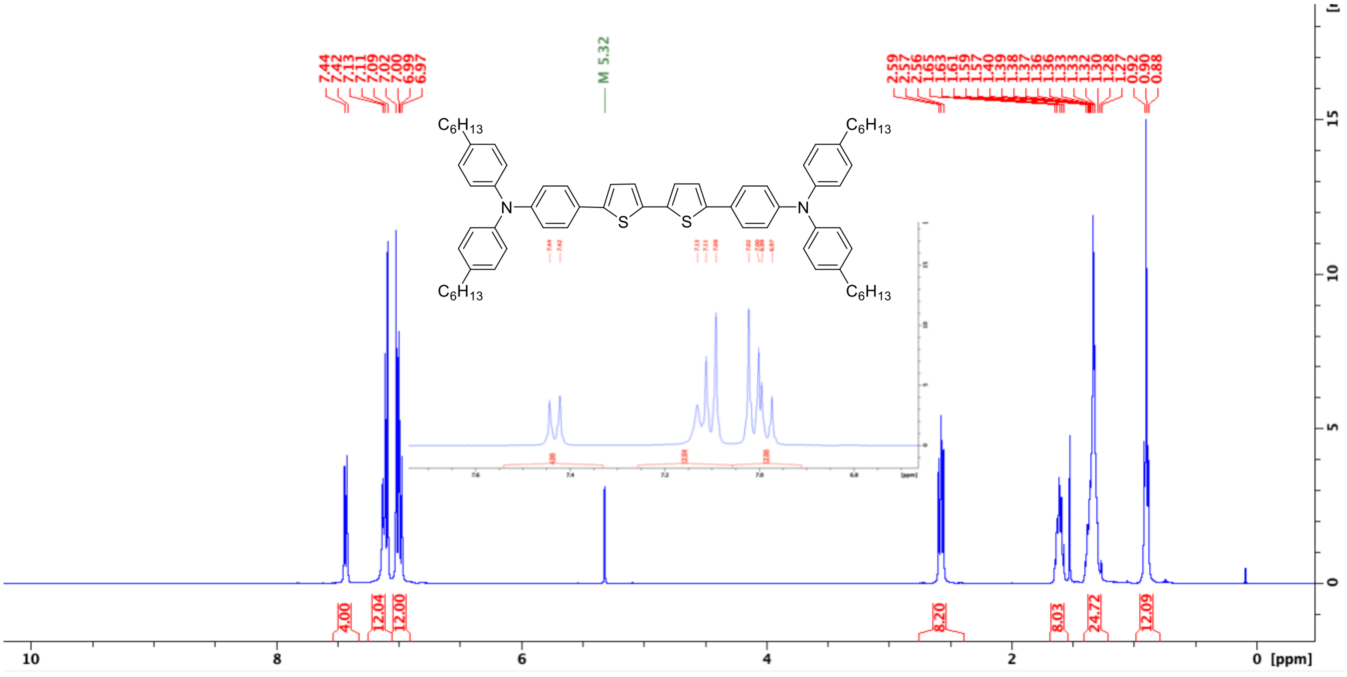

Figure S21. Proton NMR spectrum of compound **TPA-2T**.

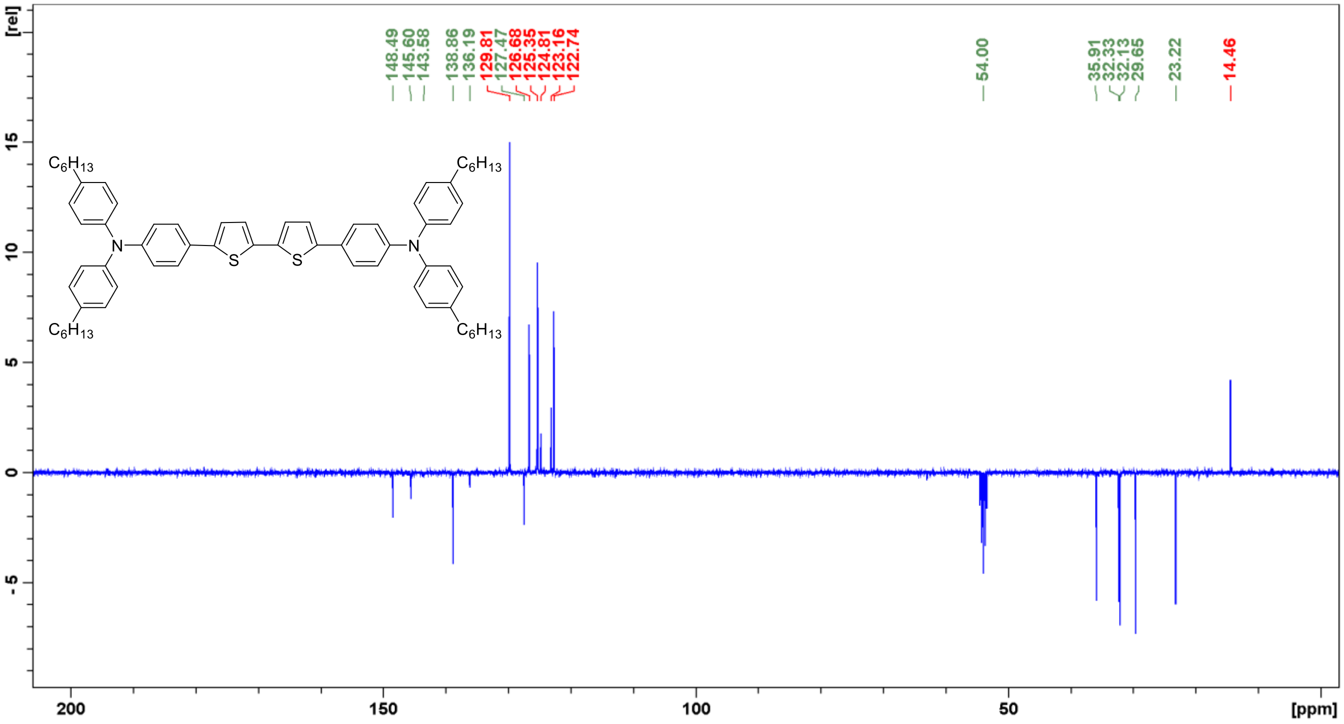

Figure S22. Carbon NMR spectrum of compound **TPA-2T**.

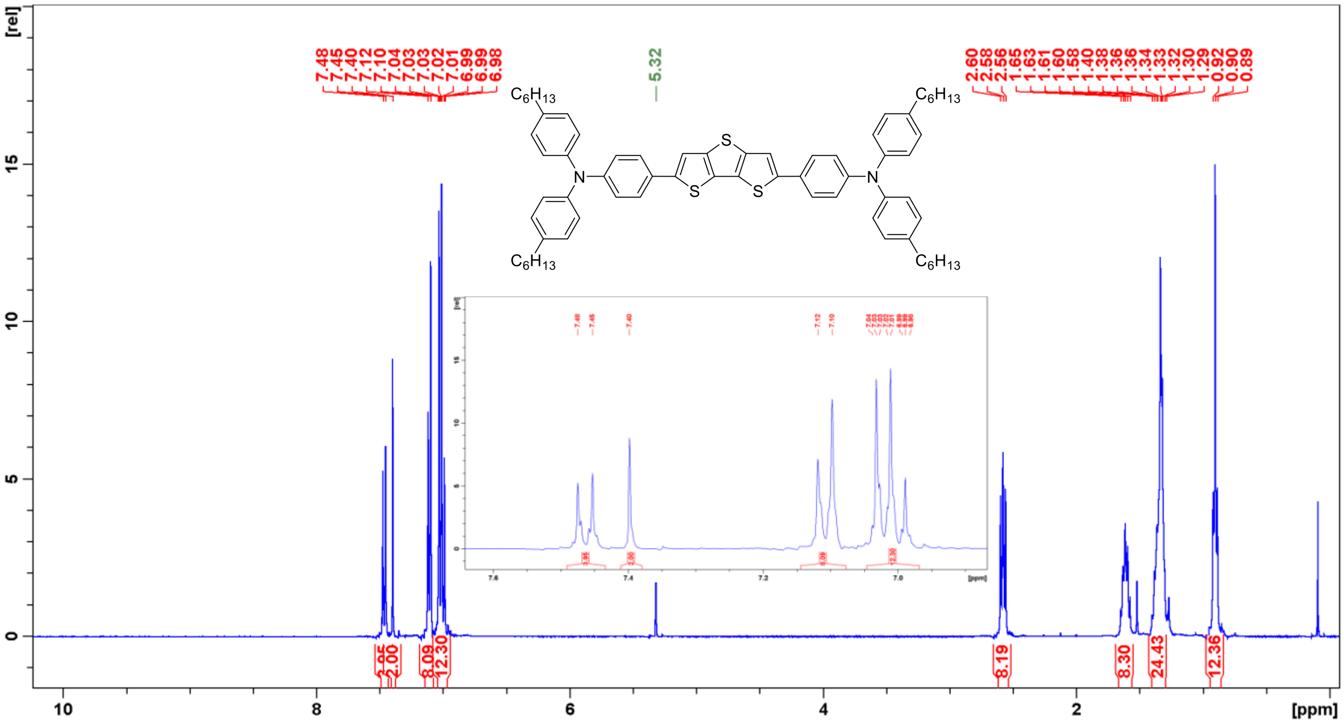


Figure S23. Proton NMR spectrum of compound **TPA-DTT**.

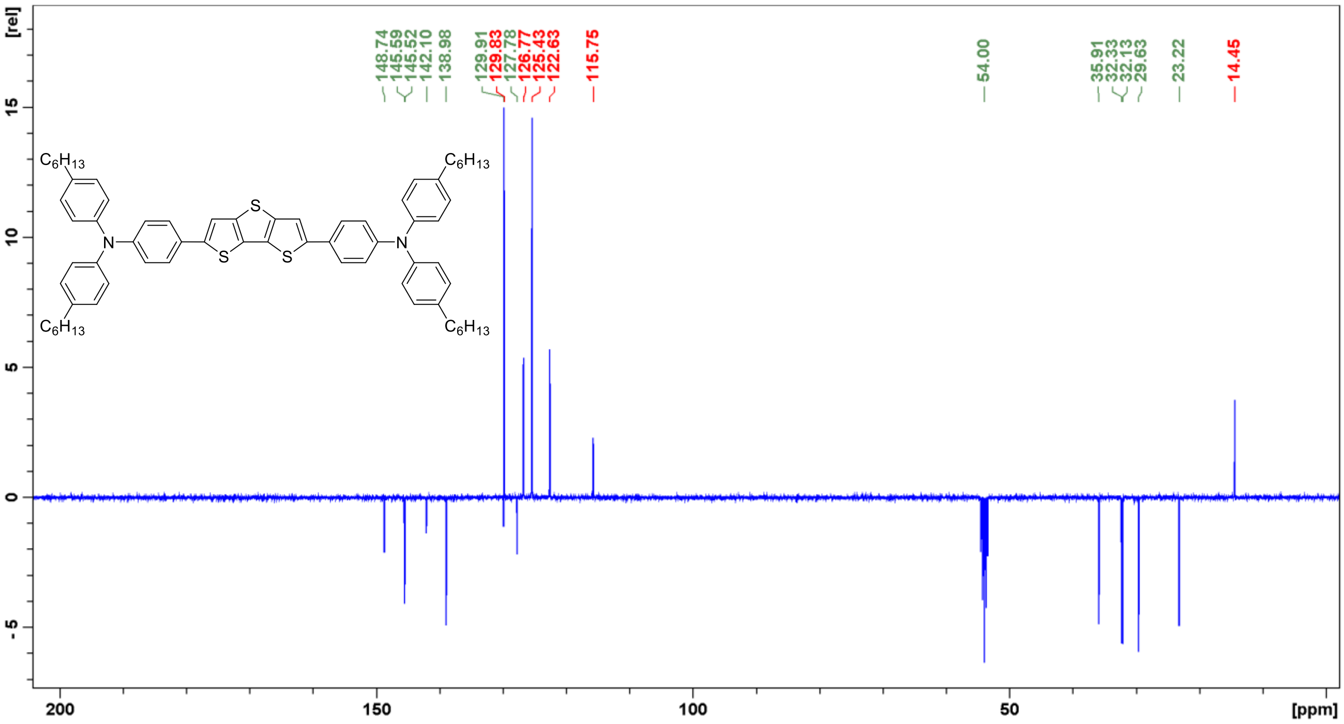


Figure S24. Carbon NMR spectrum of compound **TPA-DTT**.

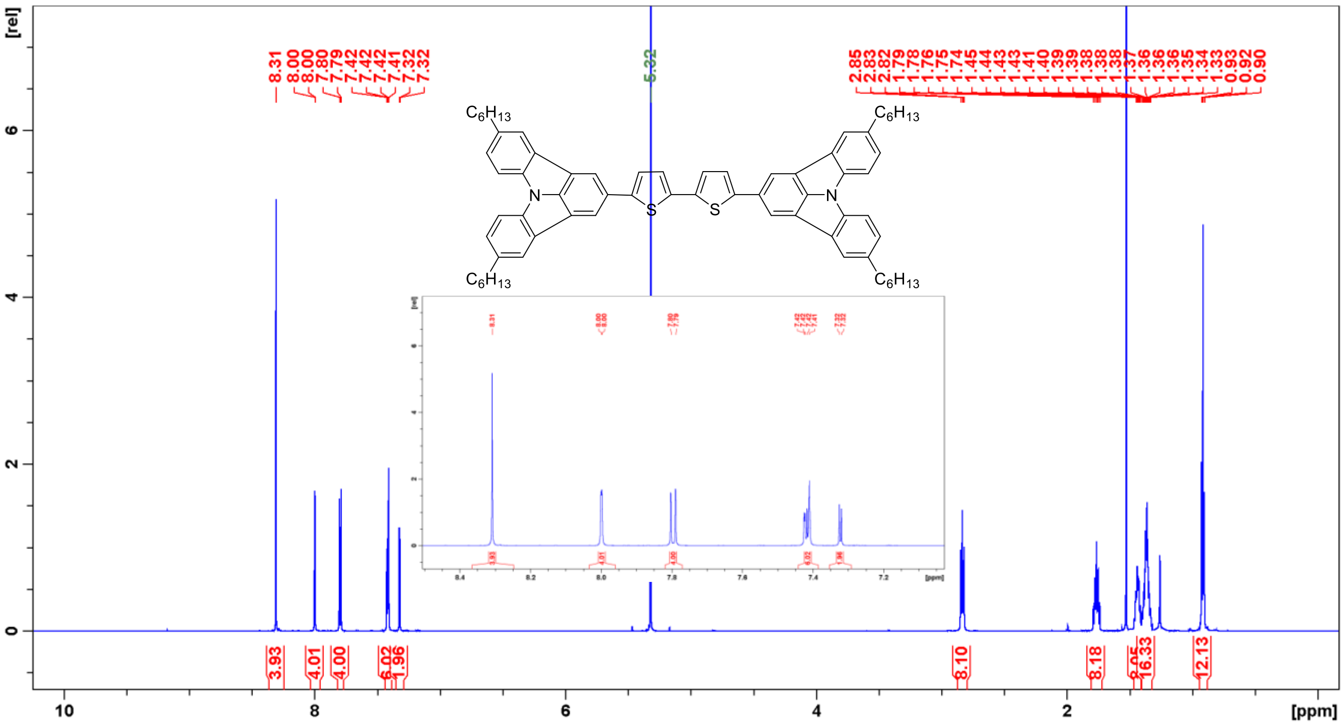

Figure S25. Proton NMR spectrum of compound **ICz-2T**.

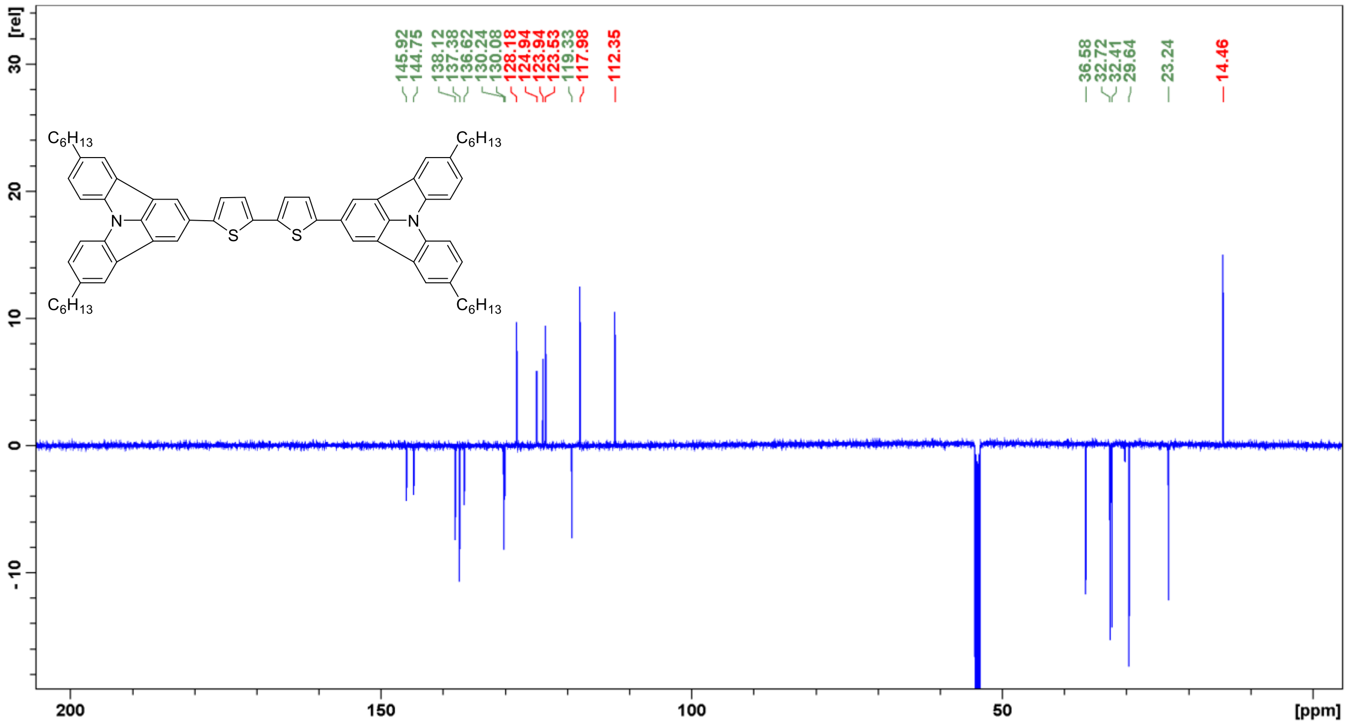

Figure S26. Carbon NMR spectrum of compound **ICz-2T**.

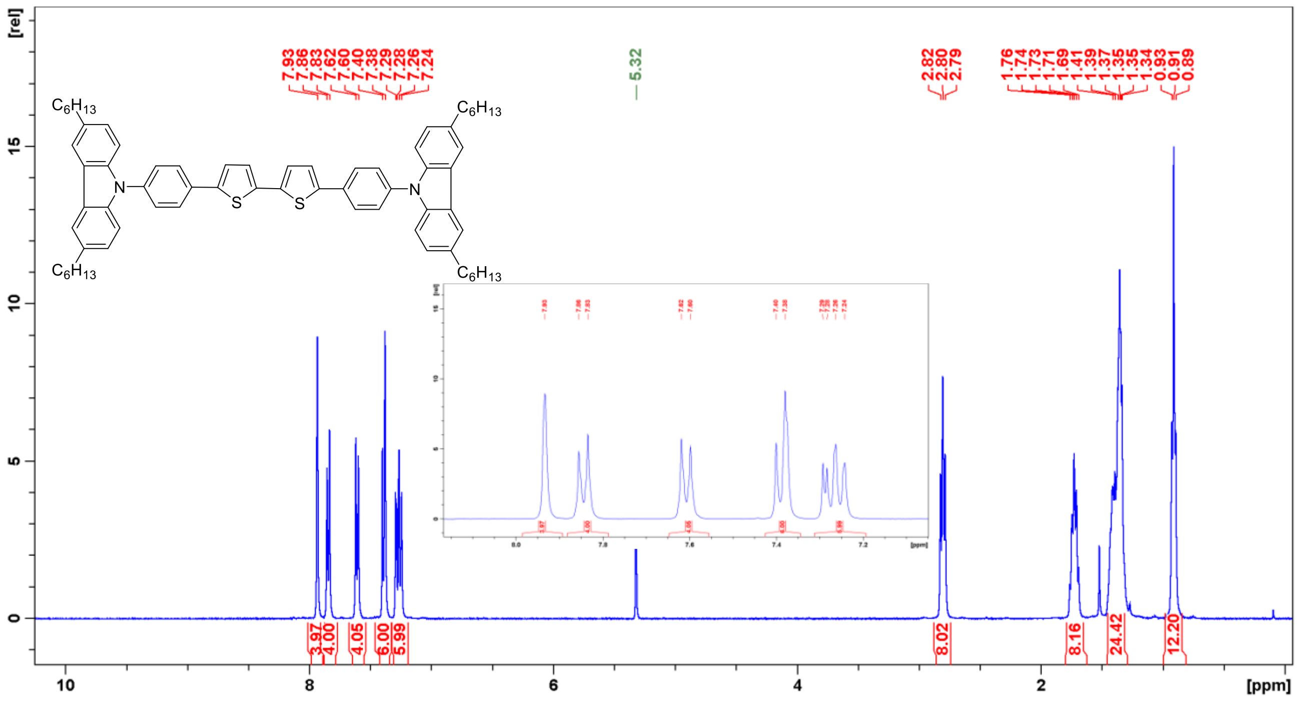

Figure S27. Proton NMR spectrum of compound **PCz-2T**.

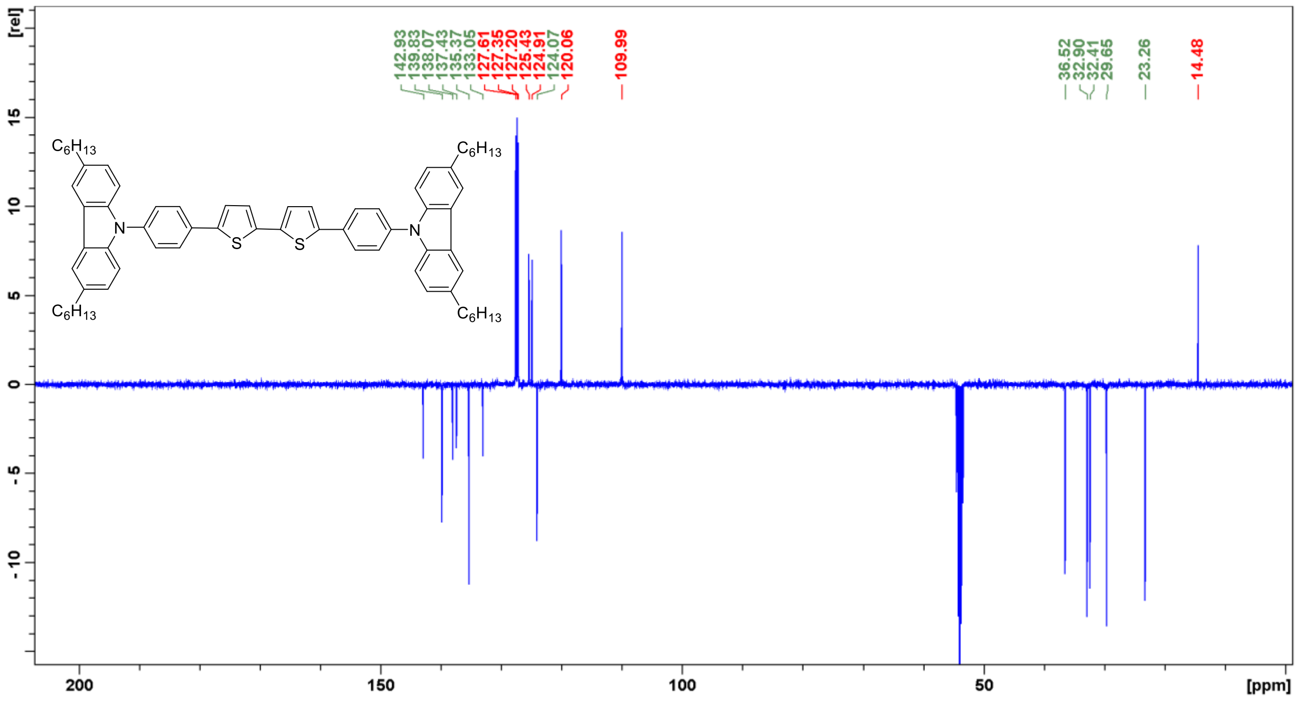

Figure S28. Carbon NMR spectrum of compound **PCz-2T**.

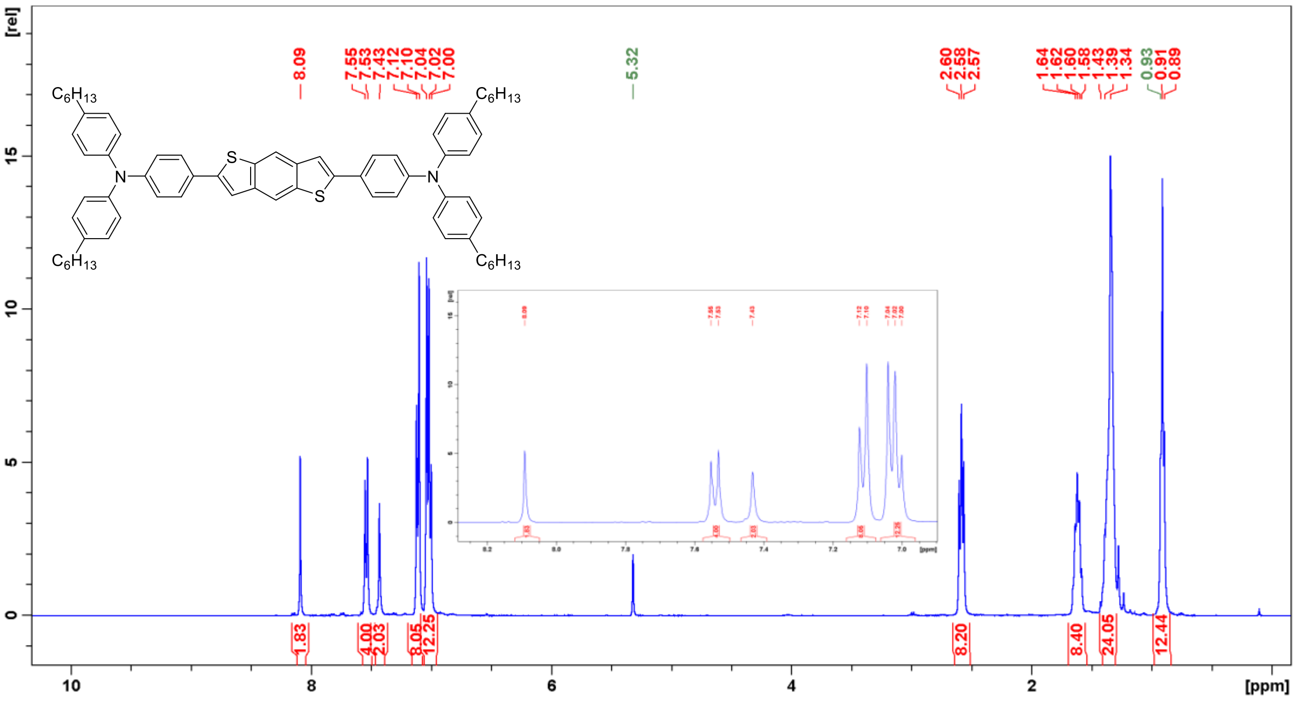


Figure S29. Proton NMR spectrum of compound **TPA-BBT**.

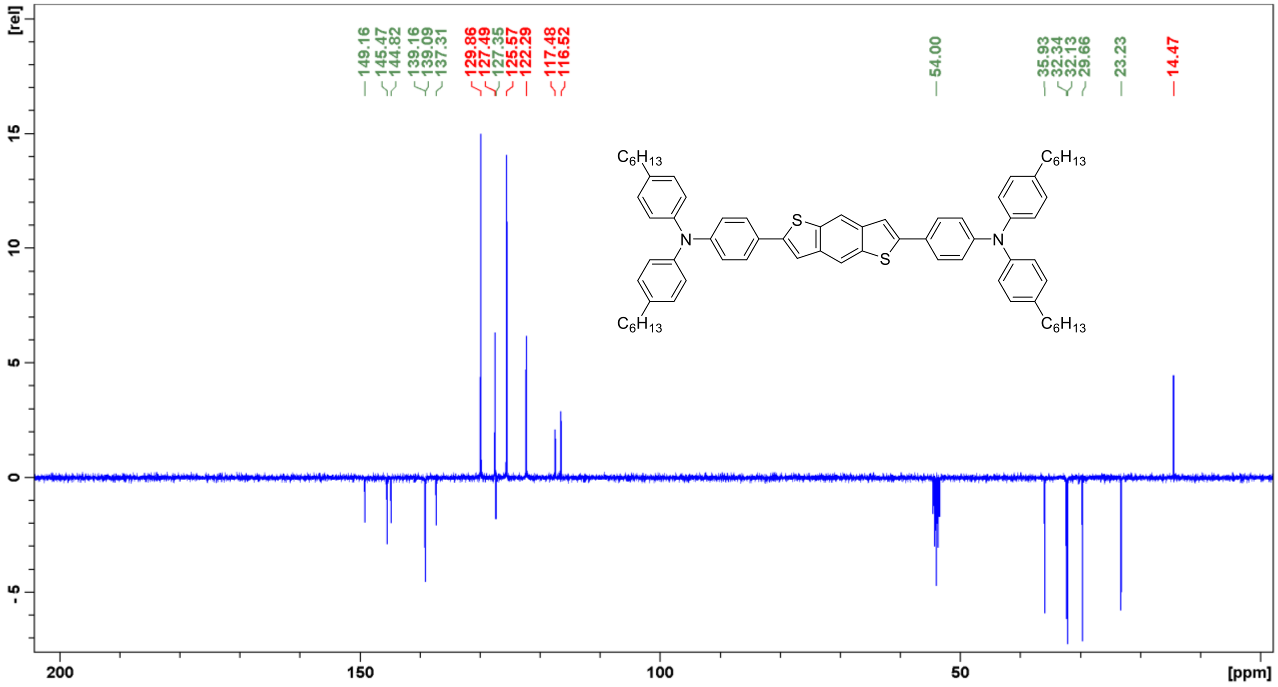


Figure S30. Carbon NMR spectrum of compound **TPA-BBT**.

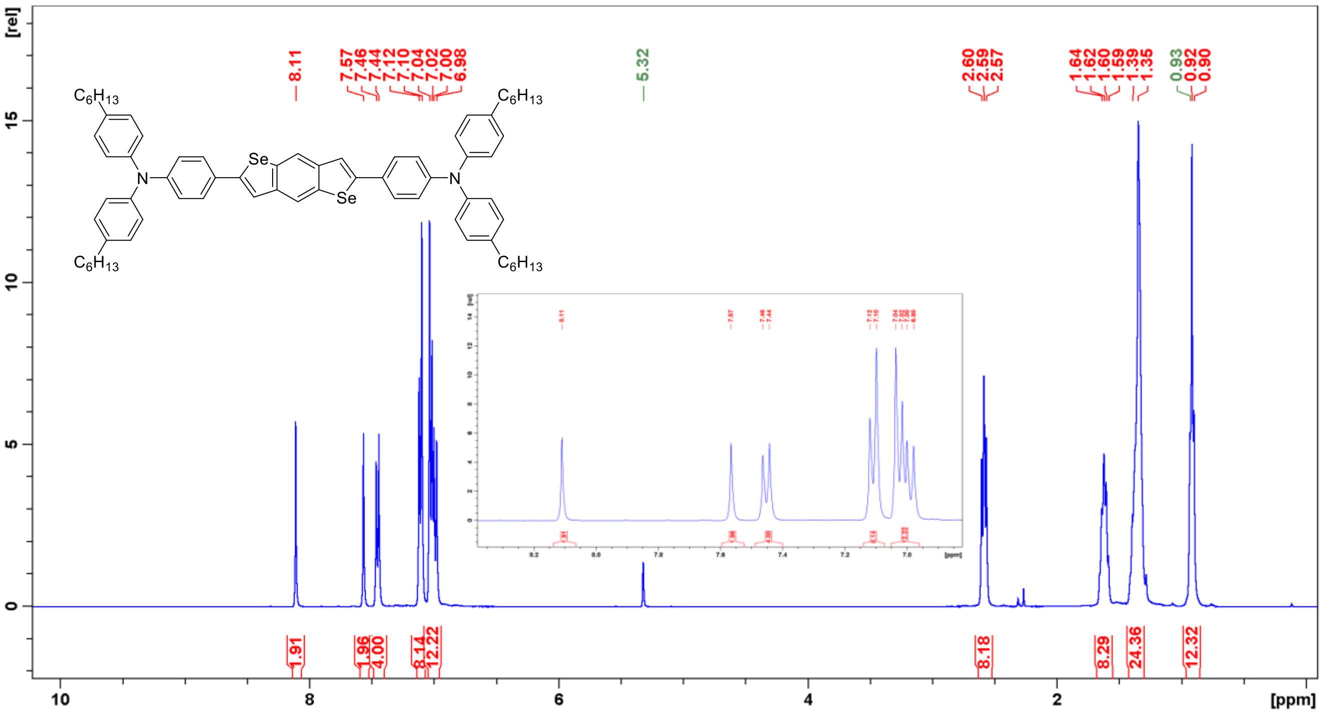

Figure S31. Proton NMR spectrum of compound **TPA-BBS**.

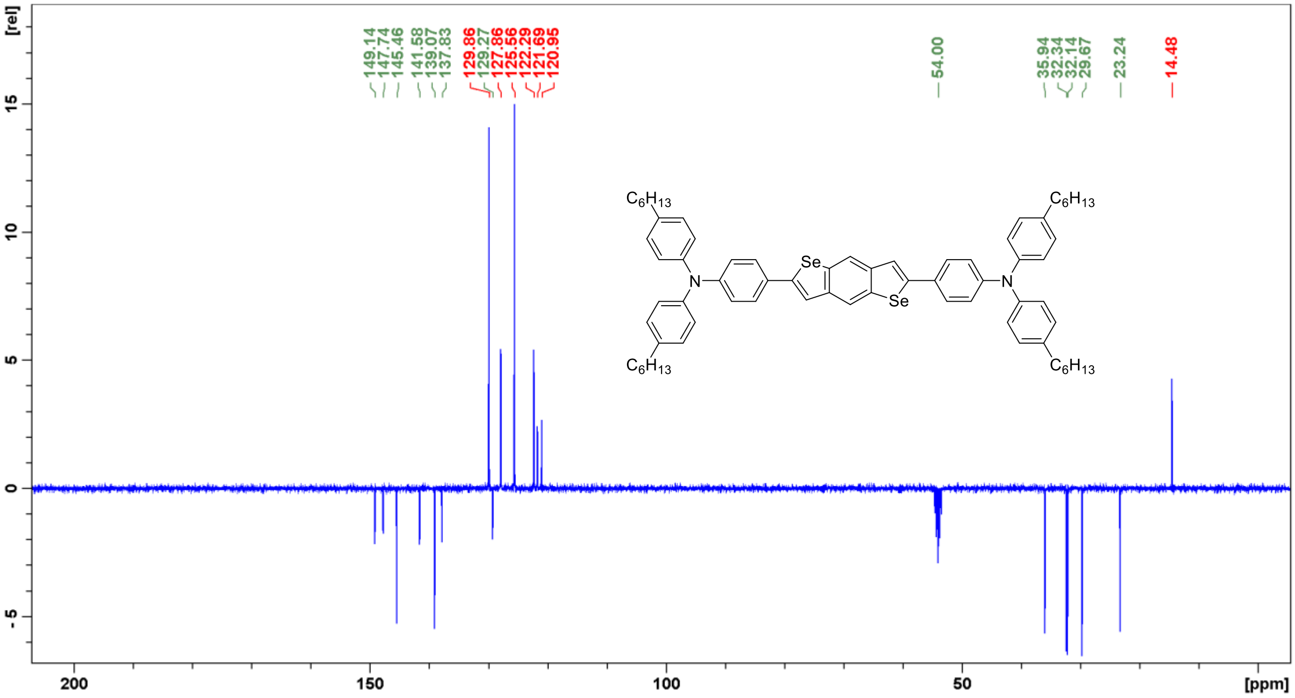

Figure S32. Carbon NMR spectrum of compound **TPA-BBS**.

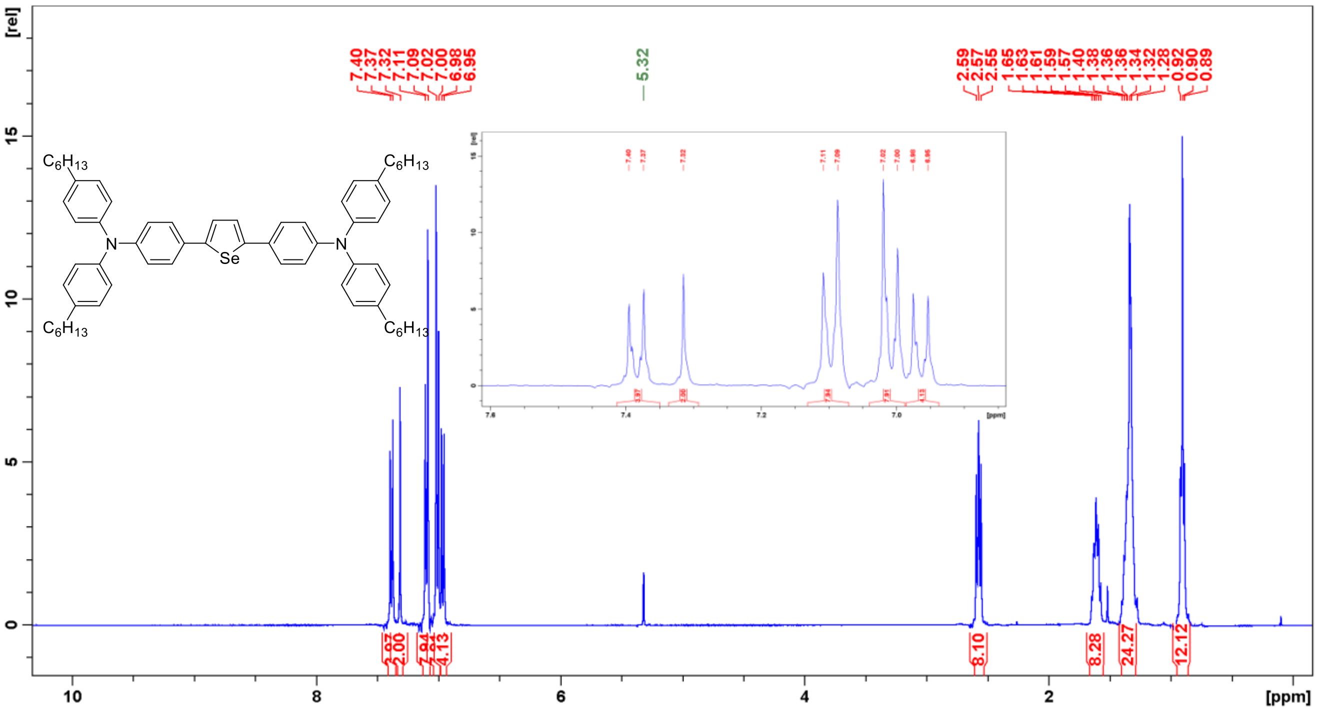

Figure S33. Proton NMR spectrum of compound **TPA-S**.

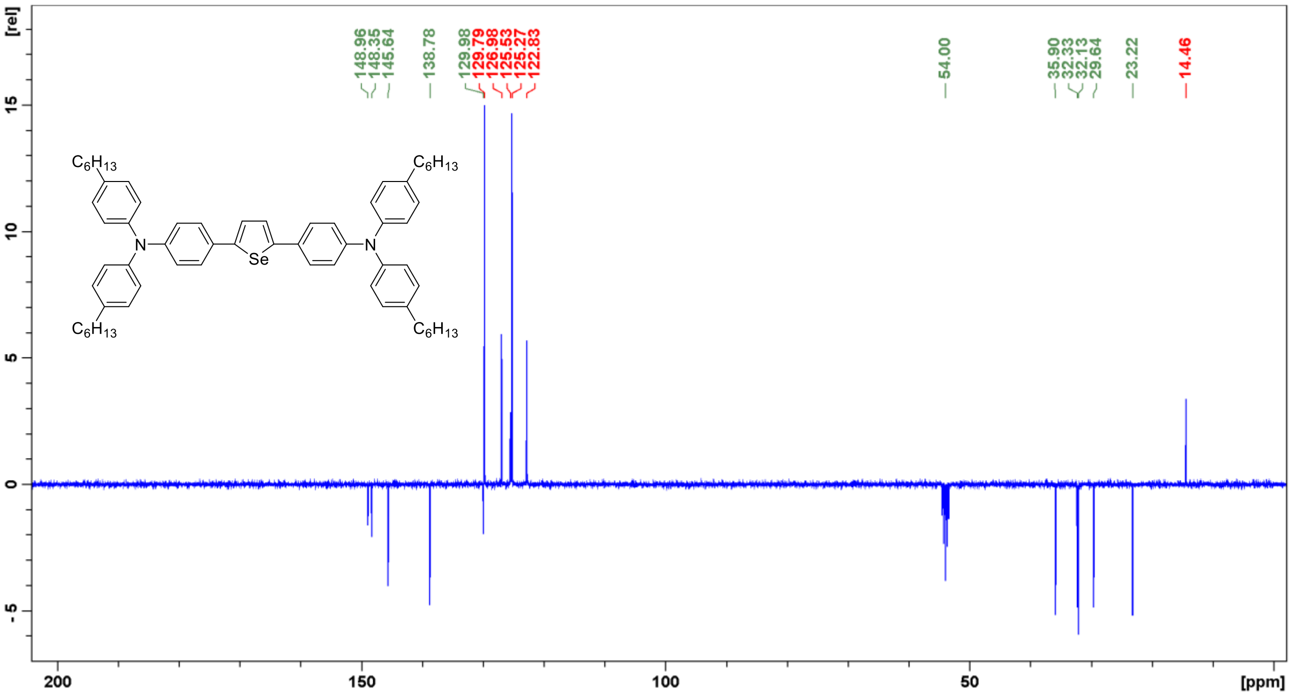

Figure S34. Carbon NMR spectrum of compound **TPA-S**.

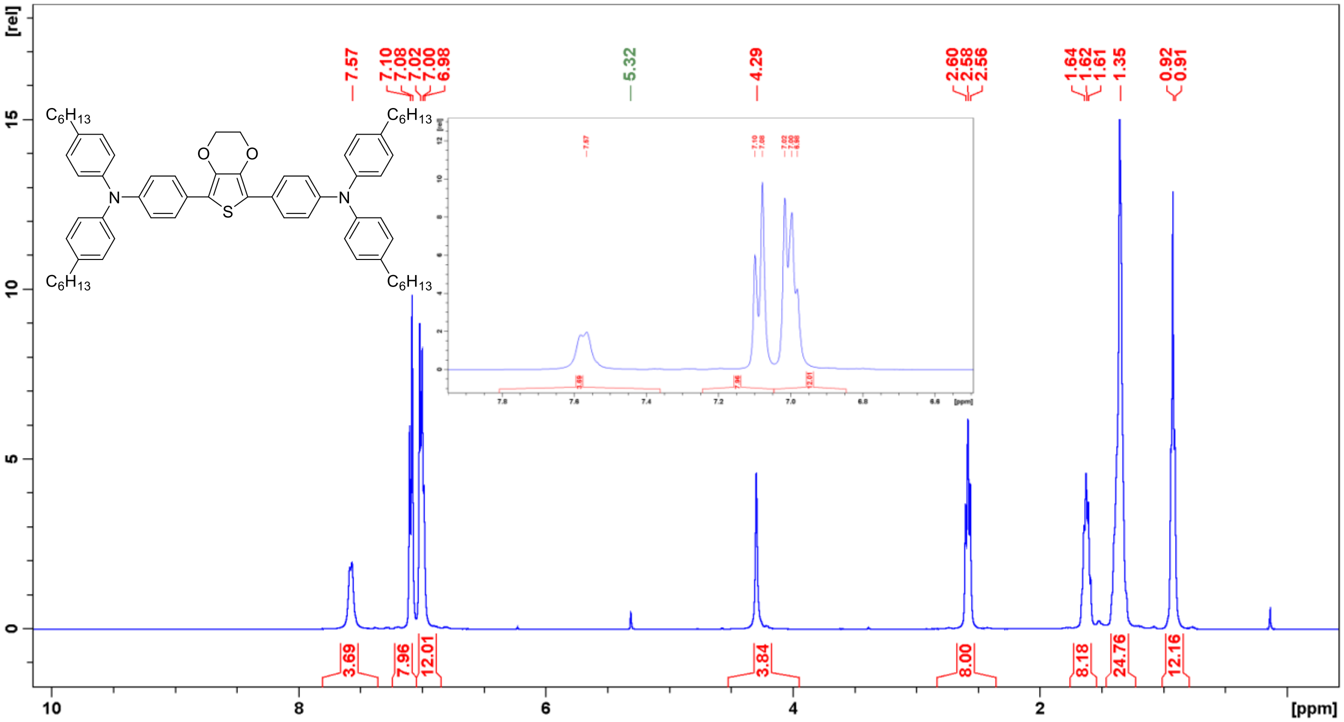


Figure S35. Proton NMR spectrum of compound **TPA-EDOT**.

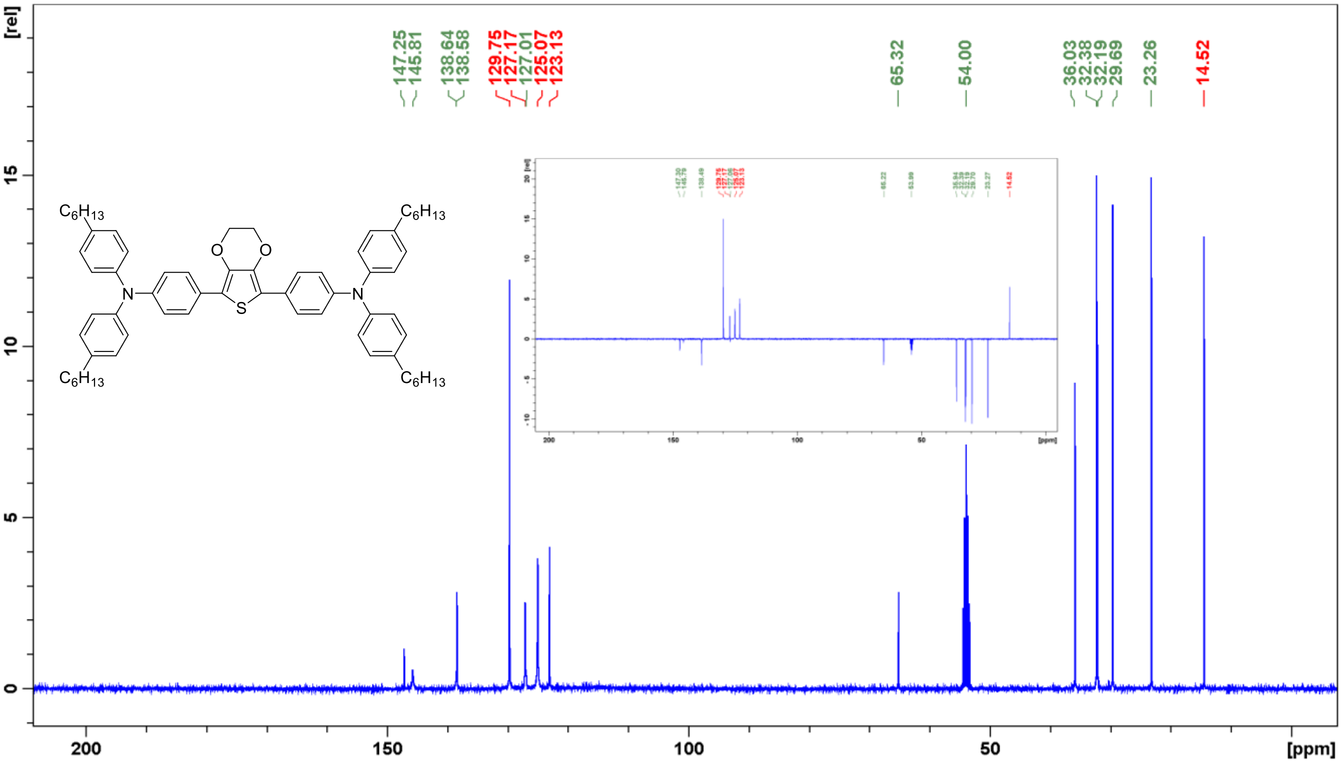


Figure S36. Carbon NMR spectrum of compound **TPA-EDOT**.

# D) Computational Chemistry

All calculations were performed in the gas phase at the DFT level of theory. The long-range corrected hybrid CAM-B3LYP[17] functional and the cc-pVDZ basis set were used throughout. $C_2$ symmetry was employed during the calculations for all the molecules. Geometrical optimizations were performed using the Gaussian09 software (Rev. D)[18] with default convergence criteria but ultra-fine numerical integration grid, since a larger grid is more suitable when optimizing more complex molecules with some soft modes, like ones involving the methyl substituents. Moreover, the D3 version of Grimmes dispersion[19] was included during the ground state geometry optimizations. The excitation energies, the transition dipole moments and the 2PA strengths calculated as single residuum of the quadratic response function ($\delta_{\mathrm{Q}R}^{(2)}$),[20–23] were obtained instead using Dalton 2015.1 code.[24] The value of the 2PA QR cross-sections were obtained from the rotationally averaged 2PA strength, $\langle\delta_{\mathrm{Q}R}^{(2)}\rangle$, using[25,26]

$$\sigma_{\mathrm{Q}R}^{(2)} = \frac{N\pi^3 \alpha a_0^5 \nu^2}{c} \langle\delta_{\mathrm{Q}R}^{(2)}\rangle \qquad (1)$$

where $N$ is an integer related mainly to the used experimental setup, α is the fine structure constant, $a_0$ is the Bohr radius (in cm), c is the speed of light (in cm/s), $\nu$ is the photon energy (in atomic units, a.u.). The obtained cross-sections were converted to Göppert-Mayer (GM) and the values reported in Figure 5 represent the maxima in the calculated spectra where the lowest 7 vertical transitions were considered.

The CAM-B3LYP functional systematically overestimates the transition energies by about 0.6 eV. This is a well-known issue of this functional,[27–29] worsened by the fact that the vertical transitions were computed in the gas phase. However, the positive of the CAM-B3LYP functional is that its quality is independent of the size of the conjugated system and the degree of charge transfer, so the error can be considered as constant offset.

# E) Structuring Tests

Table 1: A **dimensionless FOM**[47] was calculated for all spectral polymerisation threshold-values using the following specifications: repetition rate 80 MHz, pulse width 90 fs (720 nm), 75 fs (740 nm) and 70 fs, scanning speed 1000 mms$^{-1}$ (cubes) and 100 mms$^{-1}$ (wires), numerical aperture 0.4 (10x objective, woodpile cubes) and 1.4 (63x objective, nanowires). 2PA photoinitiators were applied at concentrations of 0.05 µmol g$^{-1}$, the benchmark PIs **Irg369** and **BAPO** at concentrations of 1 µmol g$^{-1}$ and 100 µmol g$^{-1}$.

| Photoinitiator | 720 nm | 740 nm | 760 nm | 780 nm | 800 nm | 830 nm | 860 nm |
|---|---|---|---|---|---|---|---|
| **63x objective / nanowires** | | | | | | | |
| **TPA-1T** | $6.78 \times 10^0$ | $6.38 \times 10^0$ | $4.23 \times 10^0$ | $50.0 \times 10^0$ | $38.6 \times 10^0$ | $5.06 \times 10^0$ | - |
| **TPA-1S** | $1.25 \times 10^1$ | $1.31 \times 10^1$ | $1.14 \times 10^1$ | $7.47 \times 10^0$ | $5.40 \times 10^0$ | $5.51 \times 10^0$ | $4.80 \times 10^0$ |
| **TPA-BBS** | $2.02 \times 10^1$ | $2.21 \times 10^1$ | $2.24 \times 10^1$ | $2.00 \times 10^1$ | $1.55 \times 10^1$ | $1.73 \times 10^1$ | $1.00 \times 10^1$ |
| **10x objective / cubes** | | | | | | | |
| **Irg369** 1 µmol g$^{-1}$ | $2.17 \times 10^3$ | $5.65 \times 10^2$ | $2.58 \times 10^2$ | $1.13 \times 10^2$ | $1.17 \times 10^2$ | - | - |
| **Irg369** 100 µmol g$^{-1}$ | $1.17 \times 10^5$ | $2.37 \times 10^5$ | $6.00 \times 10^4$ | $6.49 \times 10^4$ | $3.11 \times 10^4$ | $1.95 \times 10^4$ | $6.18 \times 10^3$ |
| **BAPO** 1 µmol g$^{-1}$ | $2.03 \times 10^2$ | $1.78 \times 10^2$ | $1.46 \times 10^2$ | $9.07 \times 10^1$ | - | - | - |
| **BAPO** 100 µmol g$^{-1}$ | $1.64 \times 10^4$ | $2.64 \times 10^4$ | $1.96 \times 10^4$ | $2.12 \times 10^4$ | $1.75 \times 10^4$ | $1.03 \times 10^4$ | $3.16 \times 10^3$ |
| **TPA-1T** | $8.68 \times 10^3$ | $6.60 \times 10^3$ | $4.90 \times 10^3$ | $4.61 \times 10^3$ | $2.54 \times 10^3$ | $4.33 \times 10^3$ | $4.81 \times 10^3$ |
| **TPA-1S** | $1.64 \times 10^4$ | $1.48 \times 10^4$ | $1.19 \times 10^4$ | $6.14 \times 10^3$ | $3.88 \times 10^3$ | $4.89 \times 10^3$ | $3.48 \times 10^3$ |
| **TPA-BBS** | $2.14 \times 10^4$ | $1.94 \times 10^4$ | $1.96 \times 10^4$ | $1.62 \times 10^4$ | $1.12 \times 10^4$ | $1.03 \times 10^4$ | $8.23 \times 10^4$ |

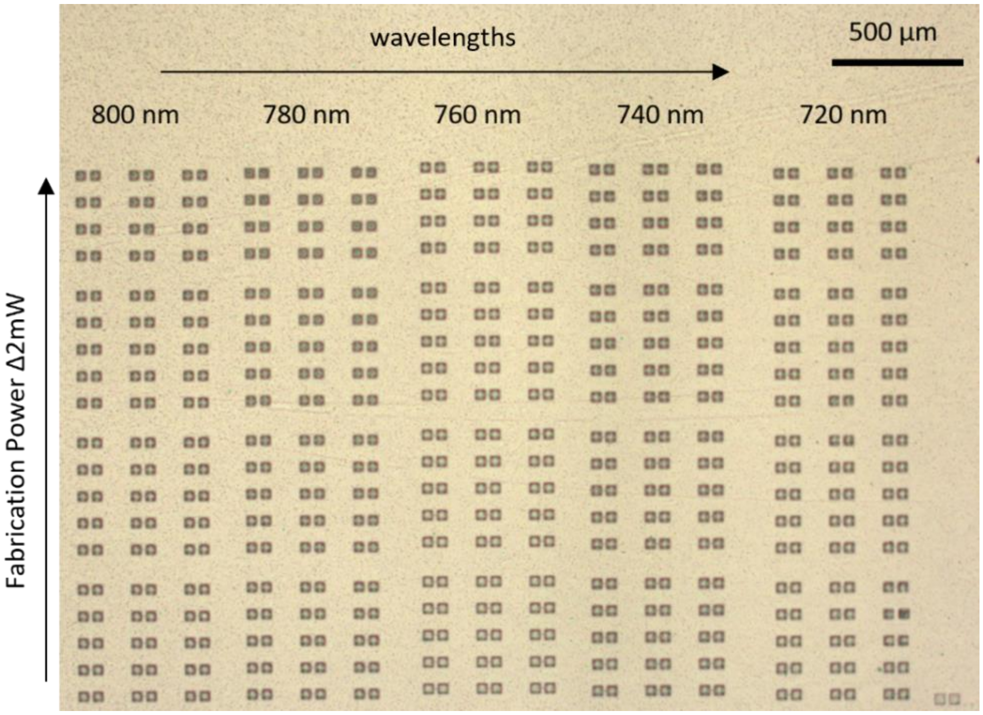


Figure S37. **Exemplary image of line-tests** to determine the spectral polymerization thresholds with a **63x objective**. Supporting pillars were first fabricated followed by a line in between. For each wavelength and power setting triplicates were fabricated. The lowest power $P_{avg}$ at which at least two stable lines were detectable after development was considered the polymerization threshold power. Lines in the bottom row were fabricated at 2 mW, the power was increased by 2 mW each row. In the presented image **TPA-1S** was tested at wavelengths 720–800 nm. Two-photon 3D printing system: MaiTai DeepSee, Objective: 63x / NA 1.4; scanning speed: 150 mm s$^{-1}$ (pillars) and 100 mm s$^{-1}$ (lines); hatch: 0.3 µm; slice distance: 0.4 µm. DIC imaging was performed with a 10x objective.

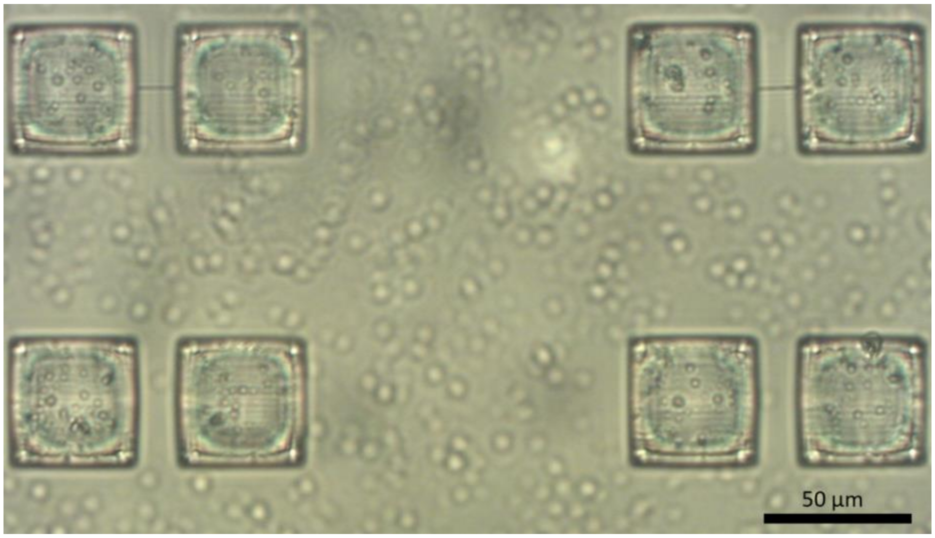


Figure S38. **Exemplary image of line-tests.** Supporting pillars were first fabricated followed by a line in between. For each wavelength and power setting triplicates were fabricated. The lowest power $P_{avg}$ at which at least two stable lines were detectable after development was considered the polymerization threshold power. The image shows line-tests of **TPA-1S** written at 740 nm. The bottom row lines were structured with 24 mW, where the polymerization threshold was not reached, and no polymerization occurred. Lines in the top row were structured with 26 mW, the power setting at which lines first occurred, indicating the polymerization threshold. Two-photon 3D printing system: MaiTai DeepSee, Objective: 63x / NA 1.4; wavelength: 740 nm; scanning speed: 150 mm $s^{-1}$ (pillars), 100 mm $s^{-1}$ (lines); hatch: 0.3 µm; slice distance: 0.4 µm. DIC imaging was performed with a 32x objective.

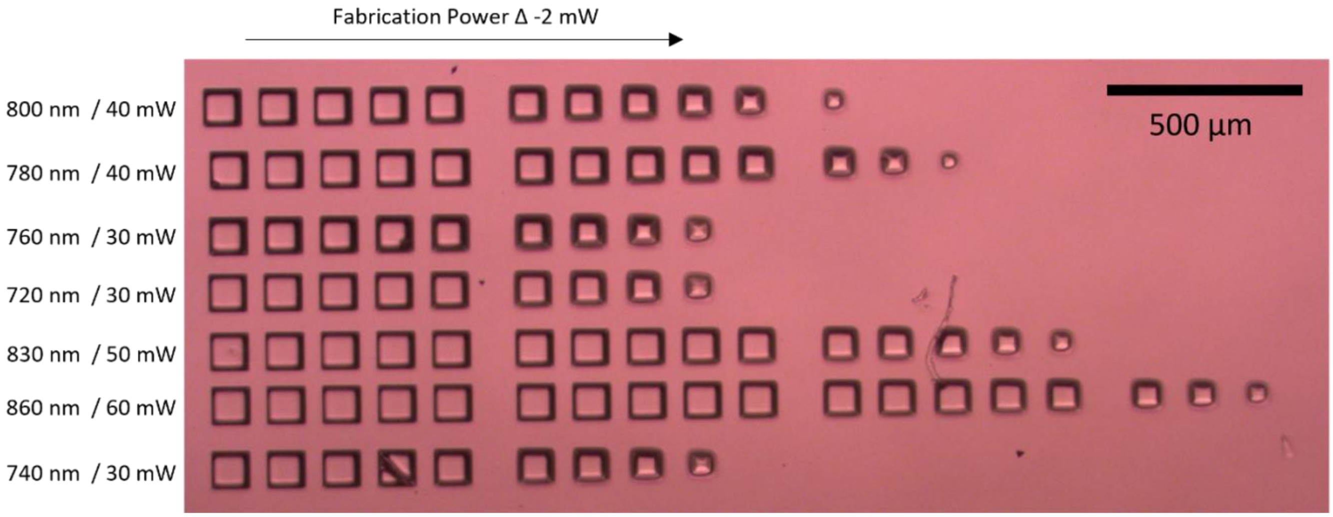


Figure S39. **Exemplary image of cube-tests** to determine the spectral polymerization thresholds with a **10x objective**. 100 x 100 x 100 $\mu m^3$ cubes were structured with decreasing power $P_{avg}$ (Δ -2 mW per cube). The lowest power $P_{avg}$ at which polymerization was still detectable after development was considered the polymerization threshold power. In the presented image **TPA-BBS** was tested at wavelengths 720-860 nm, starting power settings are indicated on the left next to the used wavelengths. Two-photon 3D printing system: MaiTai DeepSee, Objective: 10x / NA 0.4; scanning speed: 1000 mm $s^{-1}$; hatch: 0.3 µm; slice distance: 0.4 µm. DIC imaging was performed with a 10x objective.

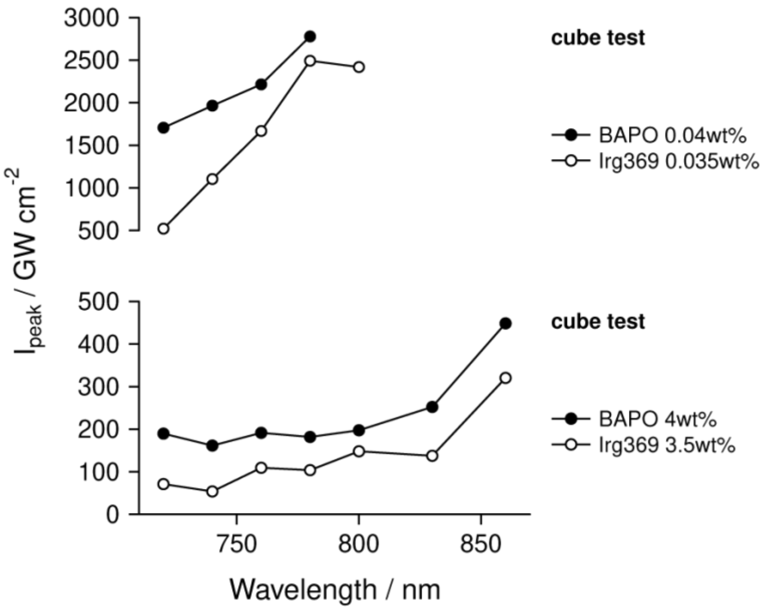


Figure S40. Spectral 2PP-threshold-tests were performed at wavelengths between 720 and 860 nm for benchmark UV-initiators **Irg369** and **BAPO** by structuring 100 x 100 x 100 µ$m^3$ cubes with a 10x objective. The commercial UV-initiators were tested at concentrations of 1 µmol $g^{-1}$ (**BAPO** 0.04 wt%, **Irg369** 0.035 wt%) and 100 µmol $g^{-1}$ (**BAPO** 4 wt%, **Irg369** 3.5 wt%).

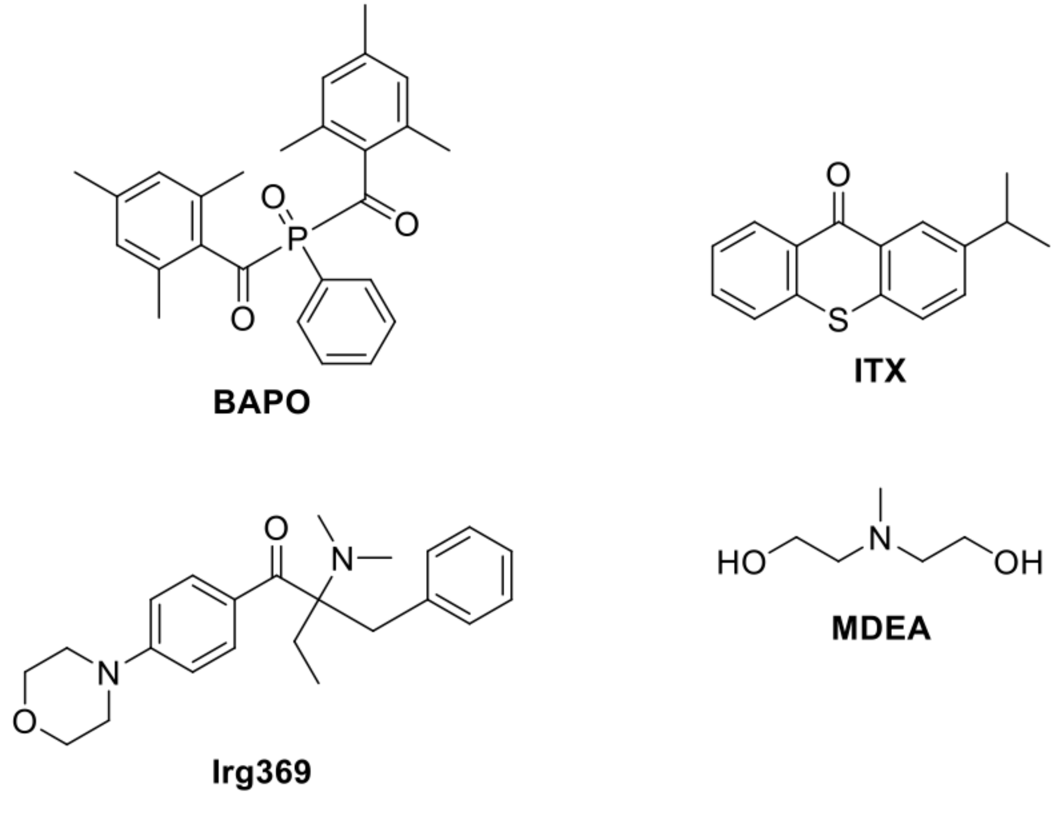


Figure S41. Molecular structures of commercial type I photoinitiators **BAPO** and **Irg369** as well as type II initiator **ITX** and tertiary amine coinitiator **MDEA**.

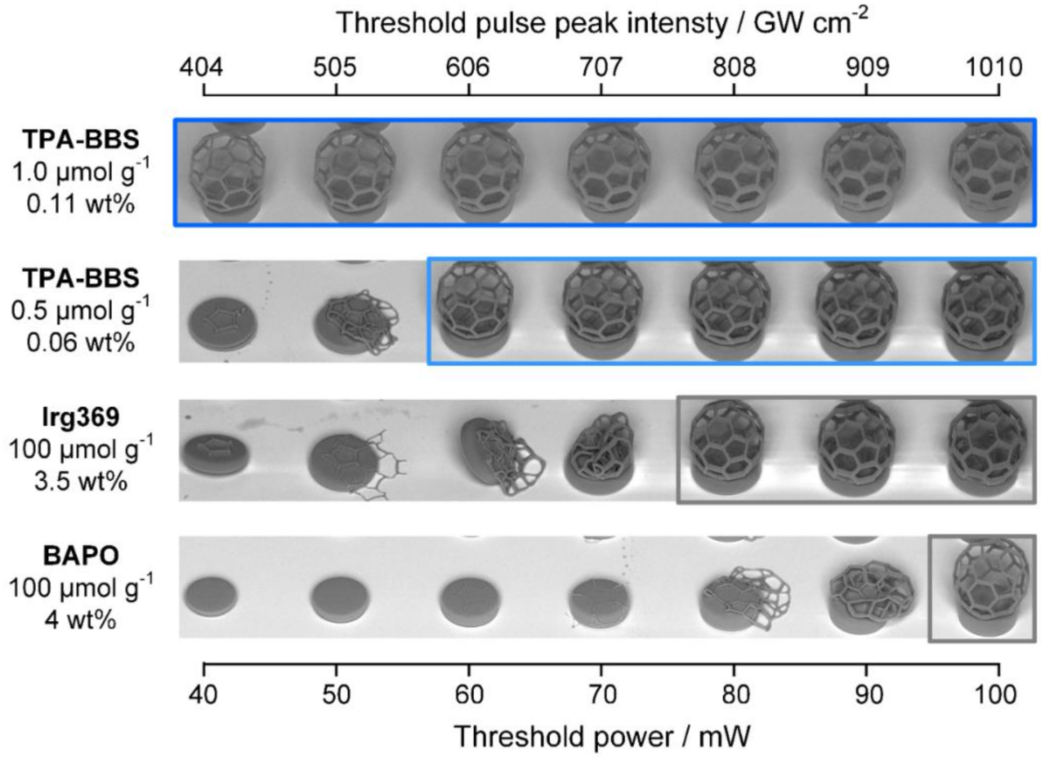


Figure S42. **Overview of form-threshold tests.** A fullerene-like structure (Ø = 250 µm) on a small platform (Ø = 250 µm, h = 40 µm) was fabricated at different laser powers $P_{avg}$ to determine the threshold laser power for formation of a stable structure. Despite its 200-times lower molar concentration (0.5 µmol $g^{-1}$) compared to commercial photocleavable UV-photoinitiators (type I) **BAPO** and **Irg369** (both 100 µmol $g^{-1}$), **TPA-BBS** gave stable structures at already 60 mW (606 GW $cm^{-2}$) compared to 100 mW (1010 GW $cm^{-2}$) and 80 mW (808 GW $cm^{-2}$), respectively, due to its high $\sigma^{(2)}_{ECS}$ at 780 nm. In doubling the concentration of **TPA-BBS** to 1.0 µmol $g^{-1}$, the threshold could further be decreased to 40 mW (404 GW $cm^{-2}$). Two-photon 3D printing system: NanoOne, Objective: 10x / NA 0.4, wavelength: 780 nm; scanning speed: 600 mm $s^{-1}$; hatch: 0.5 µm; slice distance: 2.5 µm.

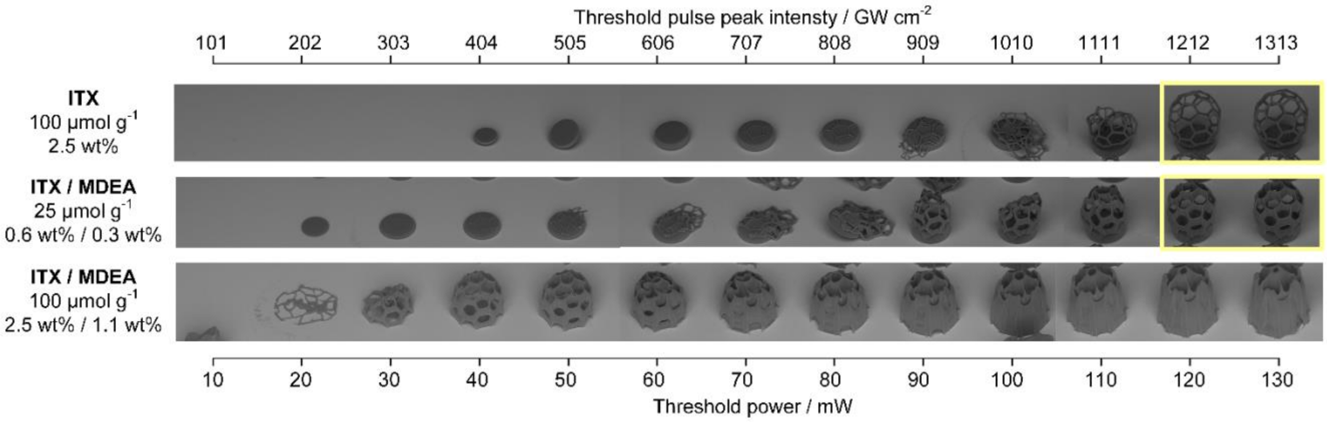


Figure S43. **Overview of form-threshold tests with ITX and coinitiator MDEA.** A fullerene-like structure (Ø = 250 µm) on a small platform (Ø = 250 µm, h = 40 µm) was fabricated at different laser powers $P_{avg}$ to determine the threshold laser power for formation of a stable structure. For comparison, also the type II initiator **ITX** was used. As recently suggested by Kiefer et al.,[30] the effect of adding the tertiary amine coinitiator **MEDA** was tested as well, which should result in a further reduction of the threshold laser power. Using only the commercial photosensitizer **ITX** at high concentration (100 µmol g$^{-1}$), stable structures formed starting at laser powers from 110 mW (1111 GW cm$^{-2}$). When amine coinitiator **MDEA** was added at equimolar concentration, the polymerization threshold was drastically increased with a non-collapsing structure already forming at a laser power of 40 mW (404 GW cm$^{-2}$). However, intense overpolymerization along the z-axis occurred,[31] leading to very distorted structures and complete loss in z-resolution, which increased proportionally with the laser power used. As a consequence, no shape fidelity could be achieved with this formulation at any laser power. When diluting this formulation to a PI concentration of 25 µmol g$^{-1}$, the performance was equal to pure **ITX** at 100 µmol g$^{-1}$ with a form-threshold of 110 mW (1111 GW cm$^{-2}$). Yet, the z-resolution was reduced due to overpolymerization, especially when compared to the formulation of pure **ITX**. Two-photon 3D printing system: NanoOne, Objective: 10x / NA 0.4, wavelength: 780 nm; scanning speed: 600 mm s$^{-1}$; hatch: 0.5 µm; slice distance: 2.5 µm.

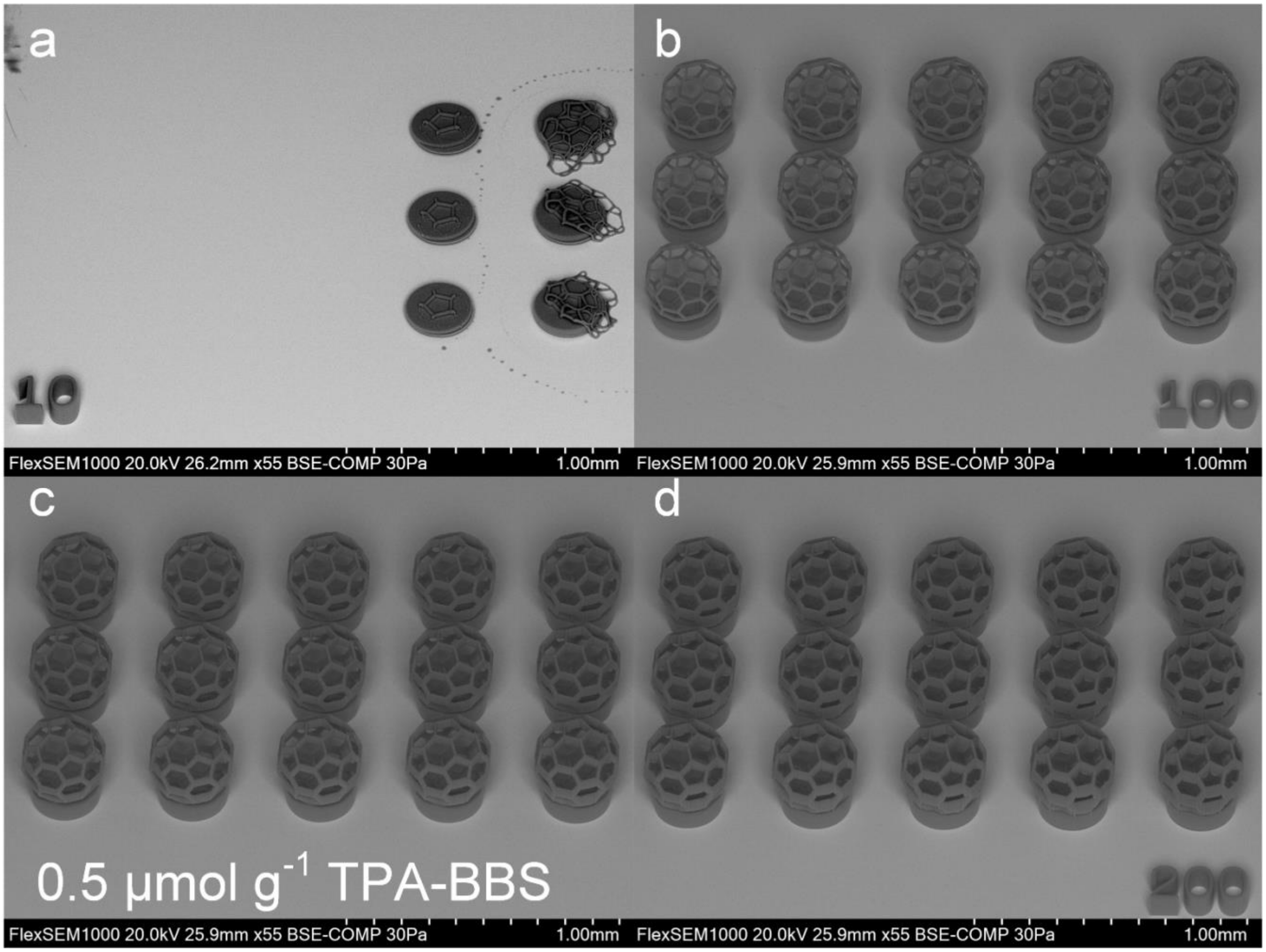


Figure S44. **Form-threshold tests of the formulation containing 0.5 µmol g$^{-1}$ TPA-BBS.** A fullerene-like structure (Ø = 250 µm) on a small platform (Ø = 250 µm, h = 40 µm) was fabricated in triplicates at different laser powers $P_{avg}$: a) 10-50 mW; b) 60-100 mW; c) 110-150 mW; d) 160-200 mW. The form-threshold was determined at 60 mW (606 GW cm$^{-2}$), as this laser power yielded 3 stable structures. With rising laser power, the voxel size increases, resulting in thicker bars. Two-photon 3D printing system: NanoOne, Objective: 10x / NA 0.4; wavelength: 780 nm; scanning speed: 600 mm s$^{-1}$; hatch: 0.5 µm; slice distance: 2.5 µm.

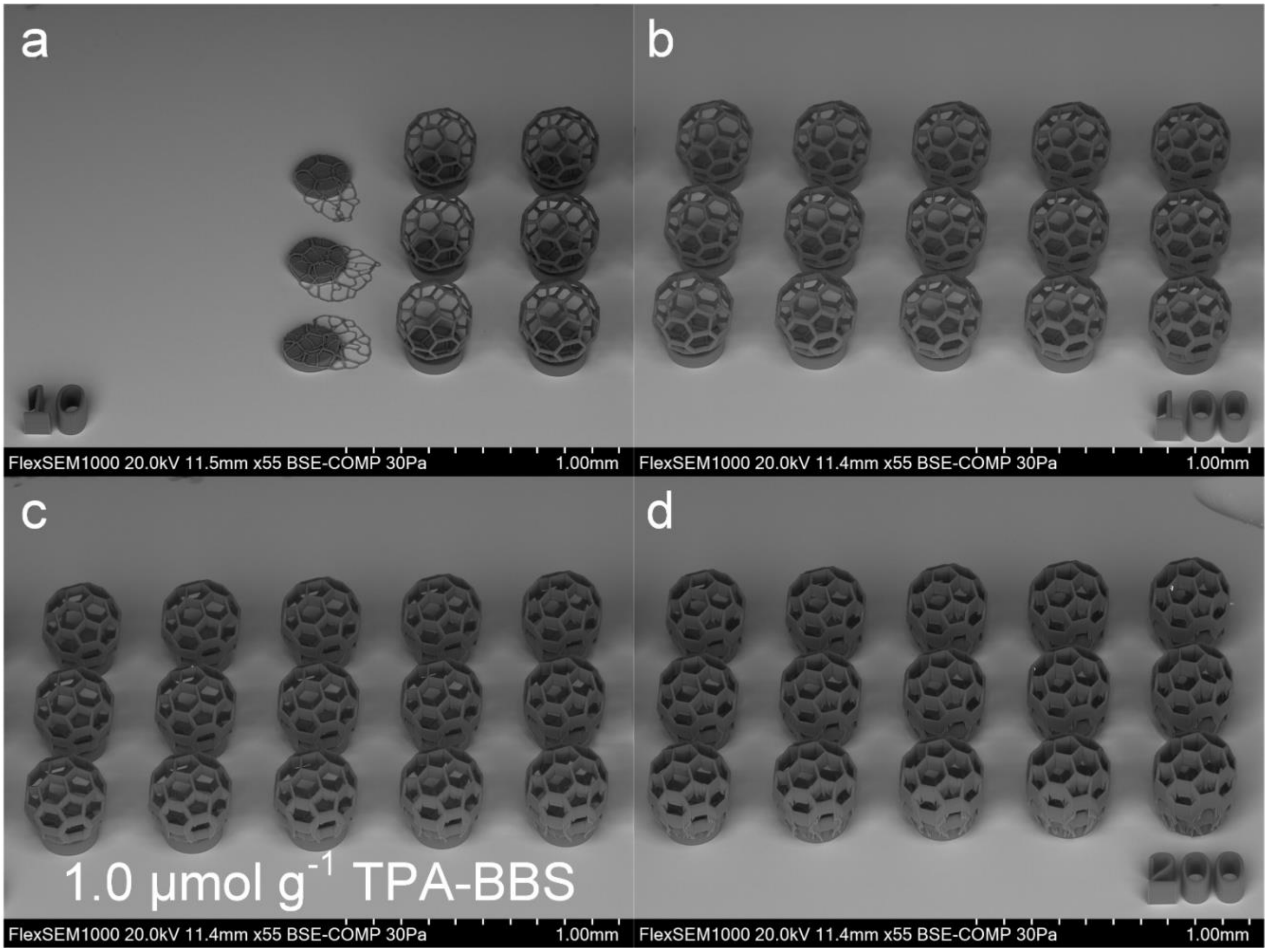


Figure S45. **Form-threshold tests of the formulation containing 1.0 µmol g$^{-1}$ TPA-BBS.** A fullerene-like structure (Ø = 250 µm) on a small platform (Ø = 250 µm, h = 40 µm) was fabricated in triplicates at different laser powers $P_{avg}$: a) 10-50 mW; b) 60-100 mW; c) 110-150 mW; d) 160-200 mW. The form-threshold was determined at 40 mW (404 GW cm$^{-2}$), as this laser power yielded 3 stable structures. With rising laser power, the voxel size increases, resulting in thicker bars. Two-photon 3D printing system: NanoOne, Objective: 10x / NA 0.4; wavelength: 780 nm; scanning speed: 600 mm s$^{-1}$; hatch: 0.5 µm; slice distance: 2.5 µm.

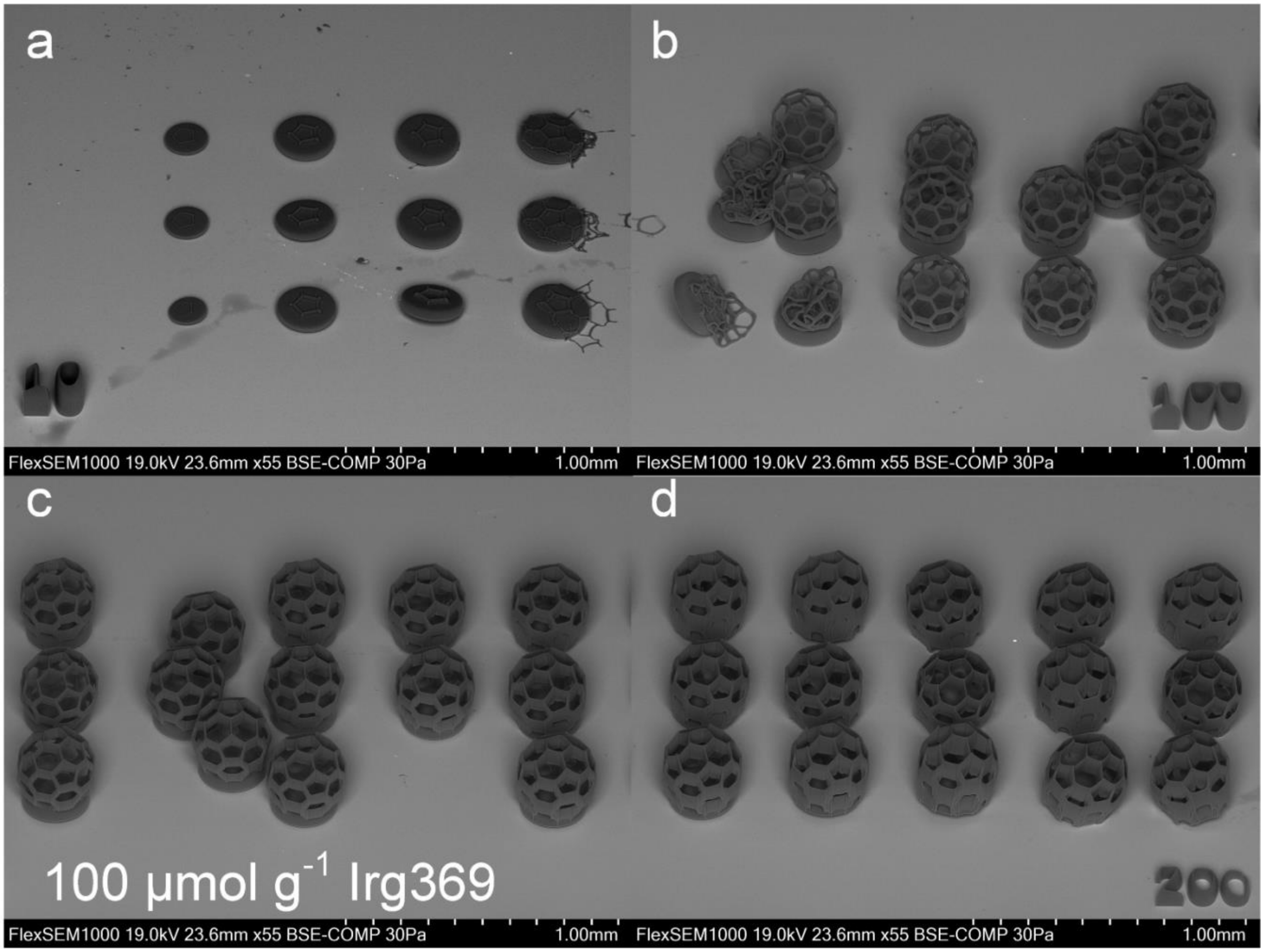


Figure S46. **Form-threshold tests of the formulation containing 100 µmol g$^{-1}$ Irg369.** A fullerene-like structure (Ø = 250 µm) on a small platform (Ø = 250 µm, h = 40 µm) was fabricated in triplicates at different laser powers $P_{avg}$: a) 10-50 mW; b) 60-100 mW; c) 110-150 mW; d) 160-200 mW. Some structures detached during imaging. Yet, the form-threshold could clearly be determined at 80 mW (808 GW cm$^{-2}$), since this laser power yielded 3 stable structures. With rising laser power, the voxel size increased, resulting in thicker bars. Two-photon 3D printing system: NanoOne, Objective: 10x / NA 0.4; wavelength: 780 nm; scanning speed: 600 mm s$^{-1}$; hatch: 0.5 µm; slice distance: 2.5 µm.

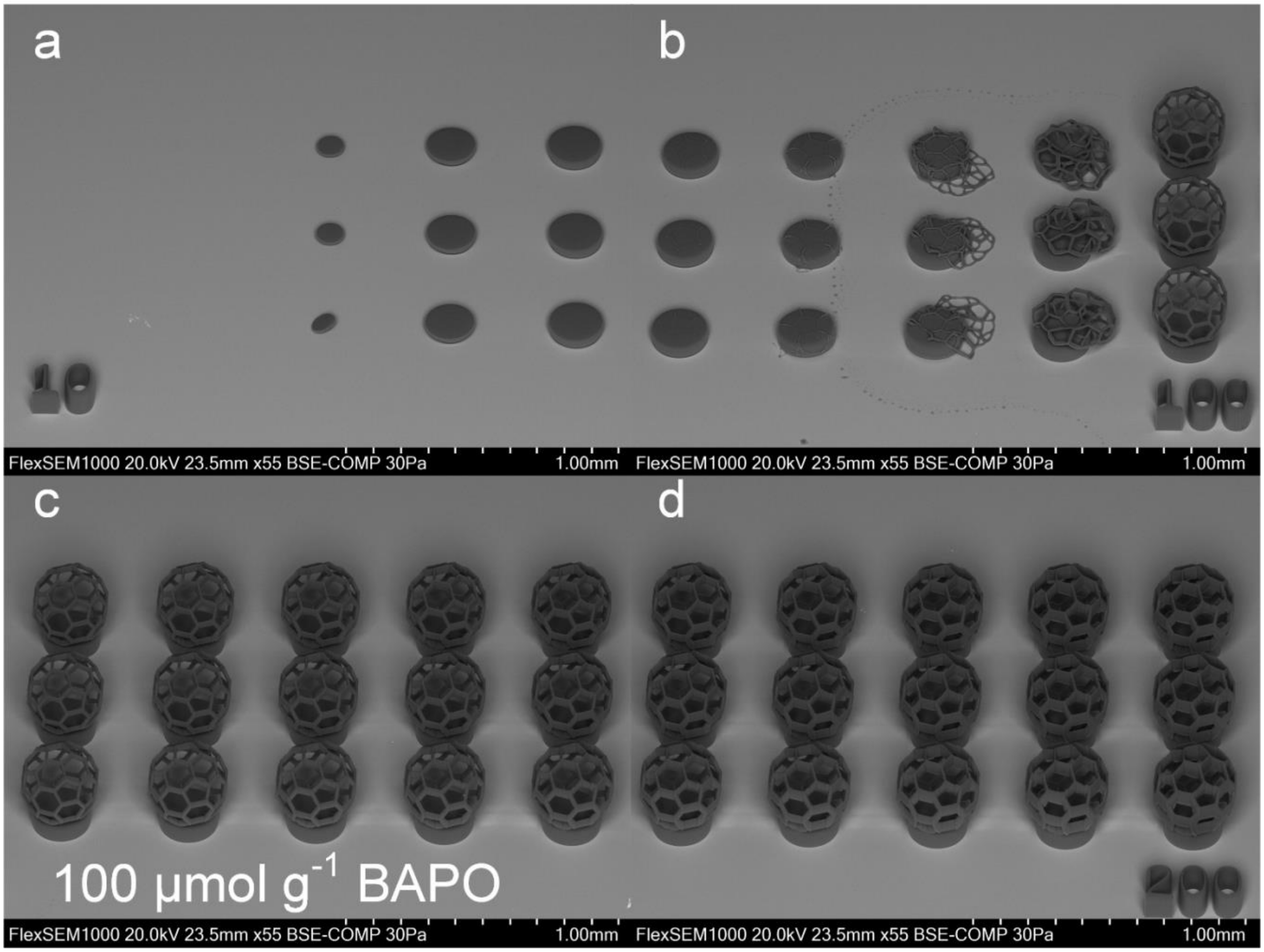


Figure S47. **Form-threshold tests of the formulation containing 100 µmol g$^{-1}$ BAPO.** A fullerene-like structure (Ø = 250 µm) on a small platform (Ø = 250 µm, h = 40 µm) was fabricated in triplicates at different laser powers $P_{avg}$: a) 10-50 mW; b) 60-100 mW; c) 110-150 mW; d) 160-200 mW. The form-threshold was determined at 100 mW (1010 GW cm$^{-2}$), as this laser power yielded 3 stable structures. With rising laser power, the voxel size increased, resulting in thicker bars. Two-photon 3D printing system: NanoOne, Objective: 10x / NA 0.4; wavelength: 780 nm; scanning speed: 600 mm s$^{-1}$; hatch: 0.5 µm; slice distance: 2.5 µm.

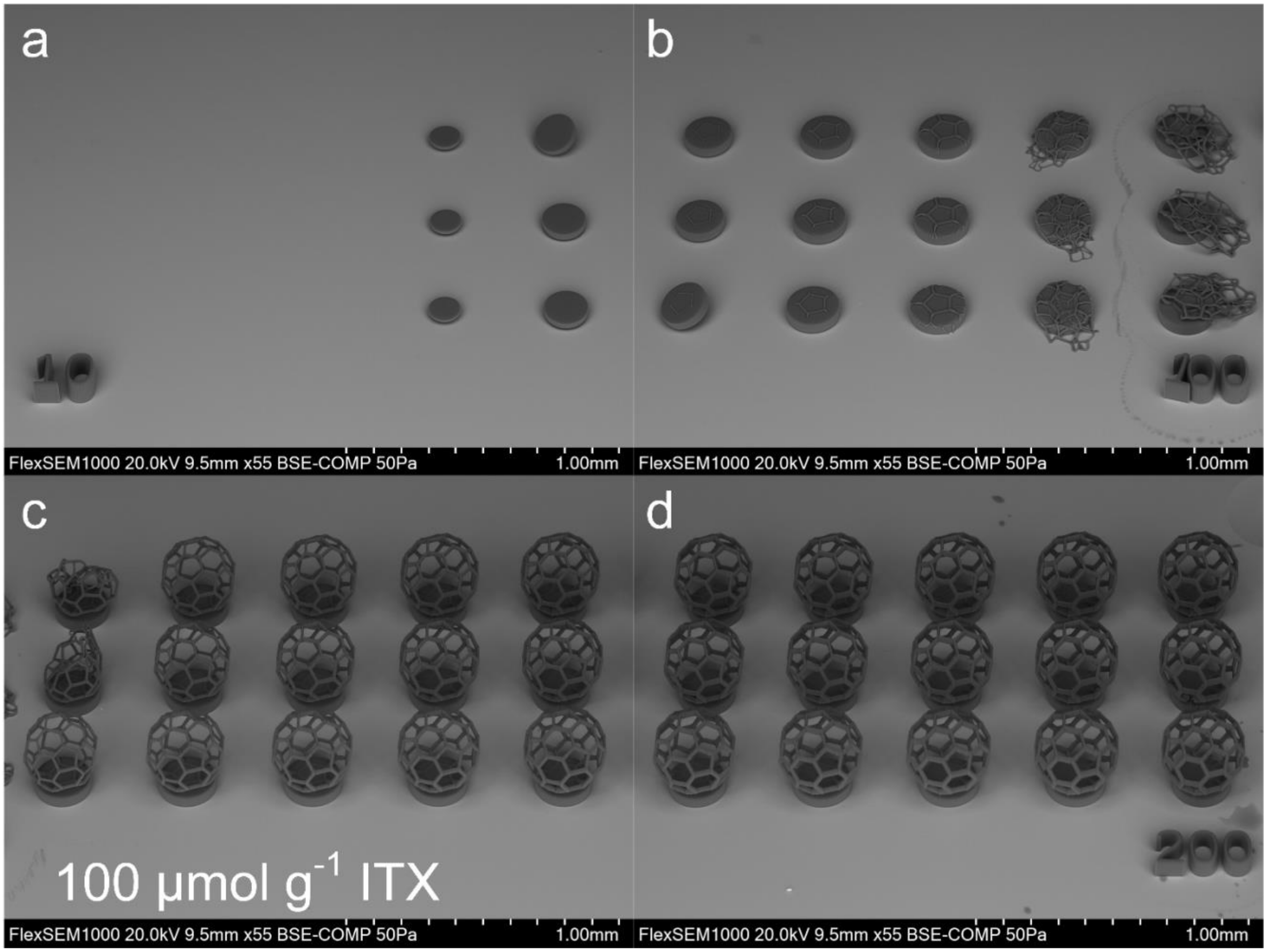


Figure S48. **Form-threshold tests of the formulation containing 100 µmol g$^{-1}$ ITX.** A fullerene-like structure (Ø = 250 µm) on a small platform (Ø = 250 µm, h = 40 µm) was fabricated in triplicates at different laser powers $P_{avg}$: a) 10-50 mW; b) 60-100 mW; c) 110-150 mW; d) 160-200 mW. The form-threshold was determined at 120 mW (1020 GW cm$^{-2}$), as this laser power yielded 3 stable structures. Interestingly, with rising laser power, the voxel size and thus the thickness of the bars increased much slower as is in the case of the other formulations. Two-photon 3D printing system: NanoOne, Objective: 10x / NA 0.4; wavelength: 780 nm; scanning speed: 600 mm s$^{-1}$; hatch: 0.5 µm; slice distance: 2.5 µm.

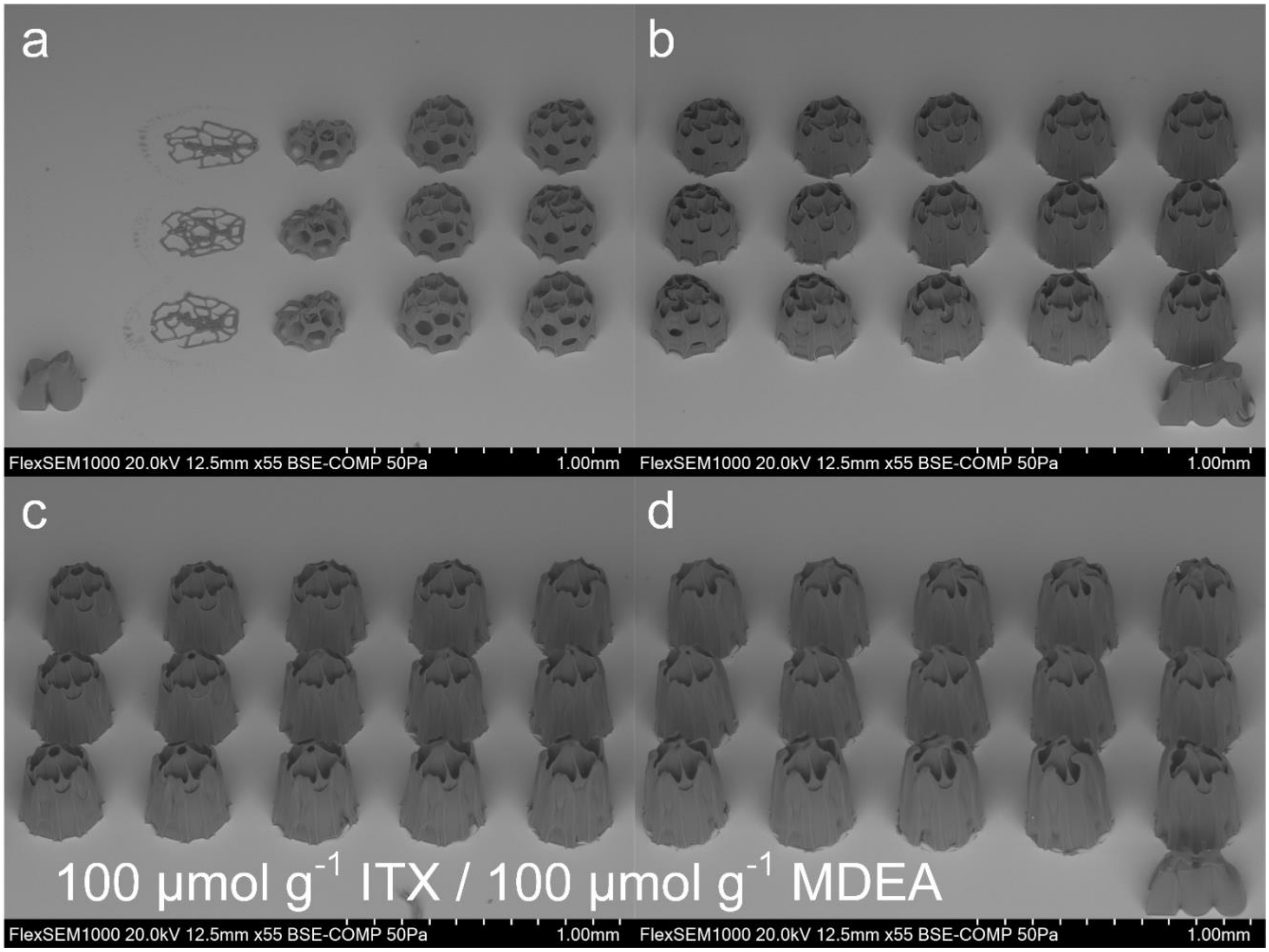


Figure S49. **Form-threshold tests of the formulation containing 100 µmol g$^{-1}$ ITX and 100 µmol g$^{-1}$ MDEA as coinitiator.** A fullerene-like structure (Ø = 250 µm) on a small platform (Ø = 250 µm, h = 40 µm) was fabricated in triplicates at different laser powers $P_{avg}$: a) 10-50 mW; b) 60-100 mW; c) 110-150 mW; d) 160-200 mW. A stable structure formed at a laser power of as low as 40 mW (404 GW cm$^{-2}$). However, no shape fidelity could be achieved with this formulation at any laser power. Extreme overpolymerization occurred, leading to very distorted structures and complete loss in z-resolution, which increased proportionally with the laser power used. Two-photon 3D printing system: NanoOne, Objective: 10x / NA 0.4; wavelength: 780 nm; scanning speed: 600 mm s$^{-1}$; hatch: 0.5 µm; slice distance: 2.5 µm.

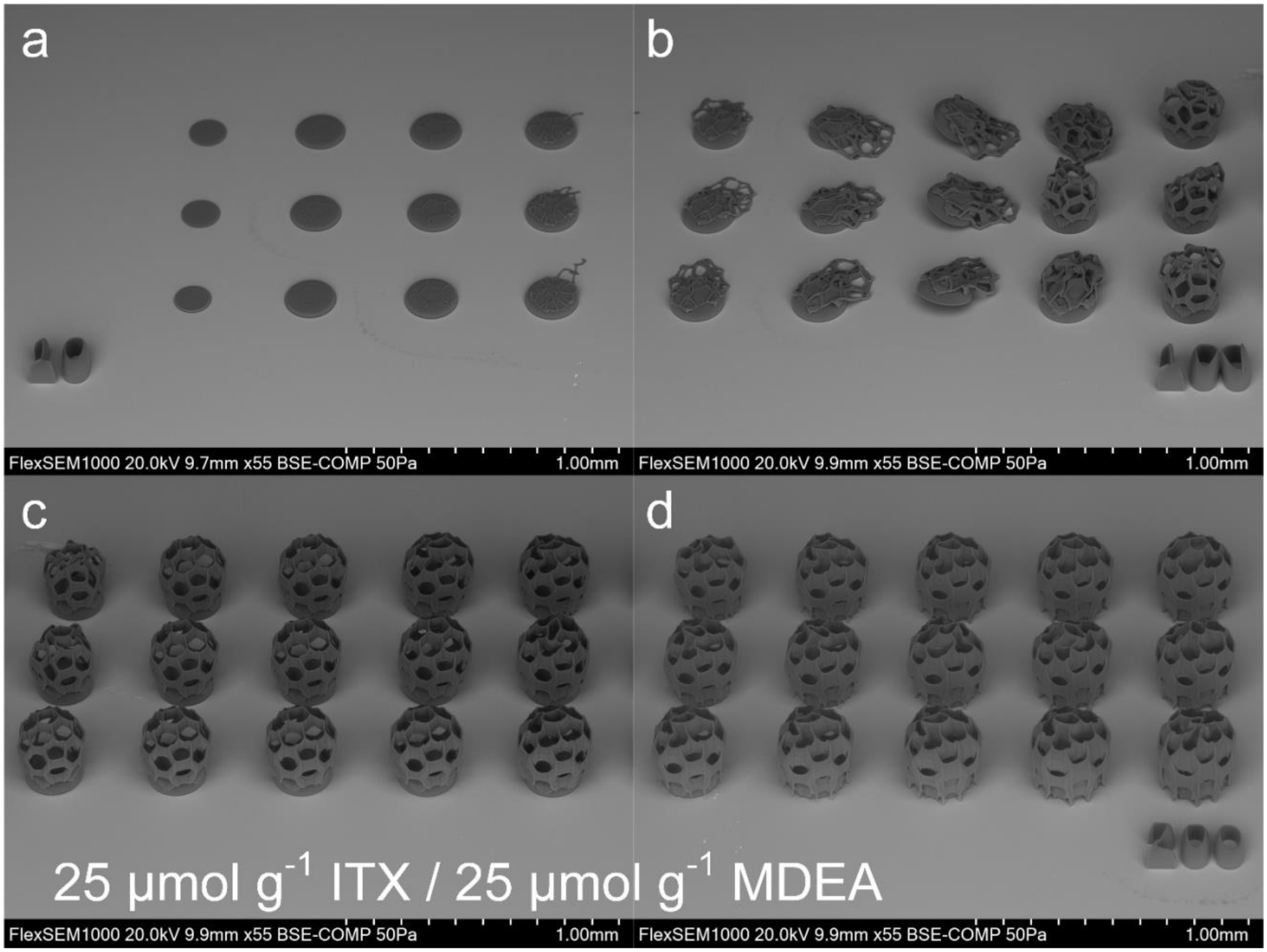


Figure S50. **Form-threshold tests of the formulation containing 25 µmol $g^{-1}$ ITX and 25 µmol $g^{-1}$ MDEA as coinitiator.** A fullerene-like structure (Ø = 250 µm) on a small platform (Ø = 250 µm, h = 40 µm) was fabricated in triplicates at different laser powers $P_{avg}$: a) 10-50 mW; b) 60-100 mW; c) 110-150 mW; d) 160-200 mW. The form-threshold was determined at 120 mW (1020 GW $cm^{-2}$), same as when 100 µmol $g^{-1}$ **ITX** with no coinitiator was used (Figure S46). Whereas the polymerization threshold could be maintained at 120 mW, while the photoinitiator concentration was reduced by a factor of 4 by adding an equimolar amount of **MDEA**, the shape fidelity was reduced. Overpolymerization occurred, leading to low z-resolution. Two-photon 3D printing system: NanoOne, Objective: 10x / NA 0.4; wavelength: 780 nm; scanning speed: 600 mm $s^{-1}$; hatch: 0.5 µm; slice distance: 2.5 µm.

# F) Photophysical Data

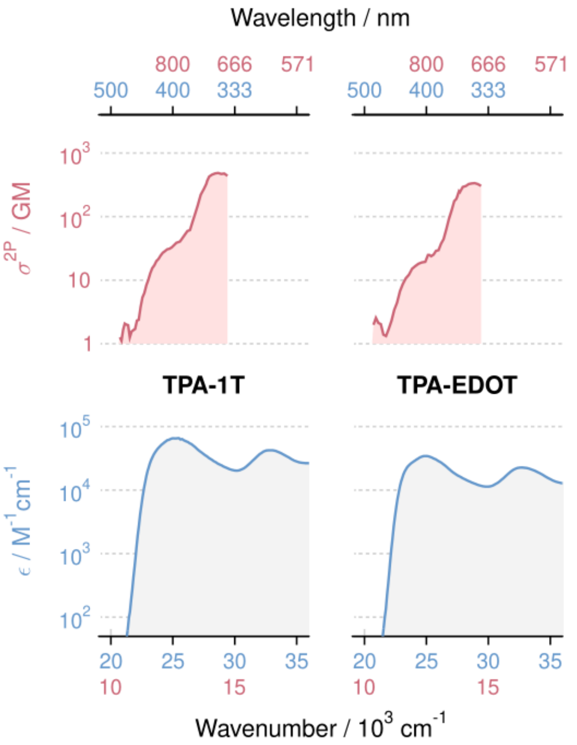


Figure S51. One and two-photon cross-sections of **TPA-1T** and **TPA-EDOT**. Solvent is THF.

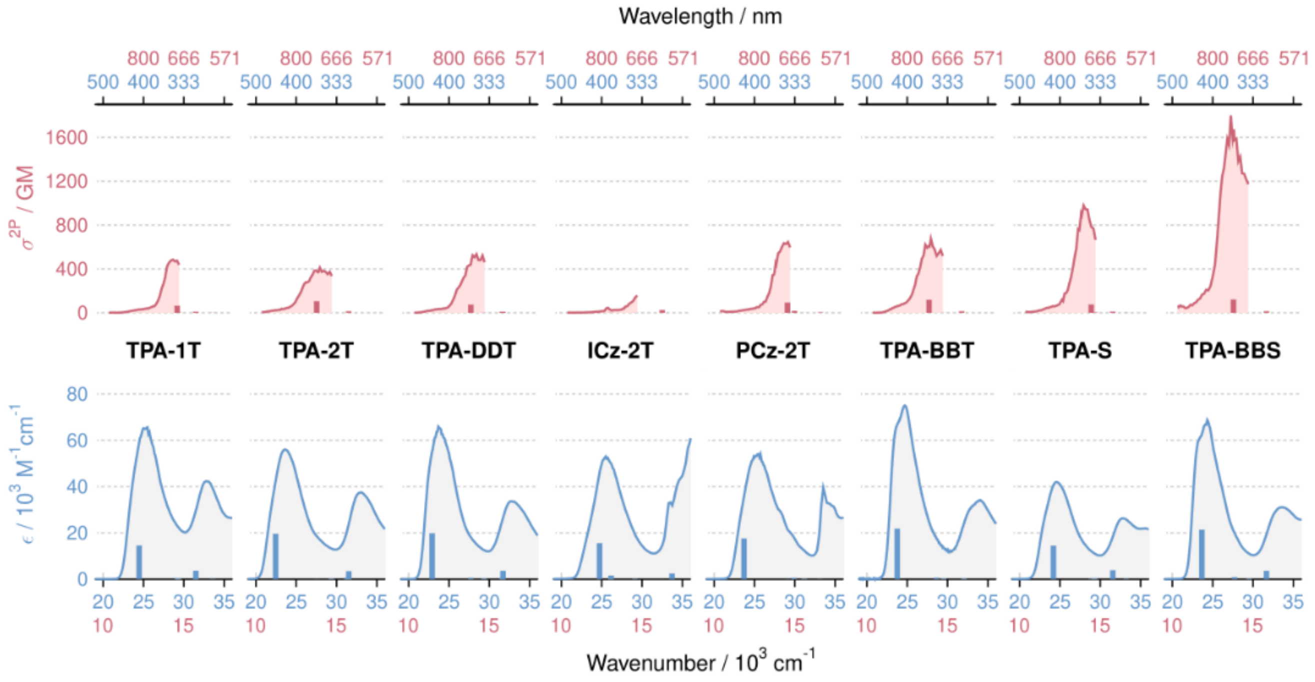


Figure S52. One and two-photon cross-sections of measured compounds shown on a linear scale. Solvent is THF. Light blue sticks are calculated 1P transitions, light red sticks are calculated 2P transitions (the calculated energies were down-shifted shifted by 4500 $cm^{-1}$).

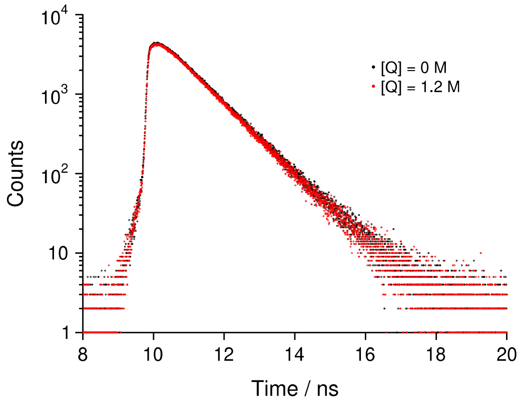


Figure S53. Time-resolved emission decay curves of **TPA-BBT** in THF in the absence (black curve) and presence of 1.2M trimethylolpropane triacrylate (TMPT, red curve) with an excitation wavelength of 405 nm using a laser diode (< 1pJ output) and detection wavelength of 510 nm.

Table 2. Photophysical data for the compounds reported. ACN = Acetonitrile; BCN = benzonitrile.

| Compound | Solvent | $\lambda_{max}$ /nm | $10^{-4}$ $\varepsilon$ /$M^{-1}$ $dm^{-3}$ | $\mu_{01}$ /D | $\tau$ /ns | $\Phi_F$ | $\mu_{10}$ /D | $10^{-8}$ $k_r$ /$s^{-1}$ | $10^{-9}$ $k_{nr}$ /$s^{-1}$ | $\Phi_{ISC}$ | $10^{-9}$ $k_{isc}$ /$s^{-1}$ | $10^{-8}$ $k_{ic}$ /$s^{-1}$ |
|---|---|---|---|---|---|---|---|---|---|---|---|---|
| **TPA-1T** | Hexane | 393 | 6.7 | 10.20 | 0.50 | 0.30 | 10.22 | 6.00 | 1.39 | 0.65 | 1.29 | 0.97 |
| | THF | 395 | 6.6 | 10.23 | 0.82 | 0.46 | 10.13 | 5.68 | 0.66 | 0.41 | 0.50 | 1.56 |
| | ACN | 395 | 6.6 | 10.21 | 1.34 | 0.61 | 9.95 | 4.59 | 0.29 | 0.32 | 0.24 | 0.47 |
| **TPA-2T** | Hexane | 413 | 5.7 | 9.77 | 0.57 | 0.25 | 9.79 | 4.32 | 1.32 | 0.40 | 0.70 | 6.20 |
| | THF | 422 | 5.6 | 9.84 | 0.52 | 0.21 | 9.80 | 4.00 | 1.51 | 0.57 | 1.09 | 4.16 |
| | ACN | 419 | 5.6 | 9.82 | 0.63 | 0.20 | 9.58 | 3.23 | 1.27 | 0.35 | 0.55 | 7.19 |
| **TPA-DDT** | Hexane | 412 | 6.8 | 9.98 | 0.87 | 0.46 | 10.05 | 5.21 | 0.62 | 0.26 | 0.30 | 3.24 |
| | THF | 417 | 6.8 | 10.10 | 0.85 | 0.44 | 10.27 | 5.17 | 0.66 | 0.33 | 0.38 | 2.78 |
| | ACN | 414 | 6.8 | 10.13 | 1.05 | 0.43 | 9.99 | 4.12 | 0.54 | | | |
| **ICz-2T** | Hexane | 386 | 5.4 | 9.67 | 0.22 | 0.09 | 9.44 | 4.55 | 4.07 | 0.86 | 3.91 | 1.62 |
| | THF | 392 | 5.3 | 9.47 | 0.25 | 0.10 | 9.05 | 4.09 | 3.65 | 0.87 | 3.52 | 1.33 |
| | BCN | 397 | 5.3 | 9.63 | 0.33 | 0.14 | 8.86 | 4.28 | 2.63 | 0.45 | 1.37 | 12.64 |
| **PCz-2T** | Hexane | 391 | 5.6 | 9.78 | 0.43 | 0.21 | 9.63 | 4.82 | 1.83 | 0.71 | 1.64 | 1.92 |
| | THF | 393 | 5.5 | 9.68 | 0.41 | 0.18 | 9.19 | 4.37 | 2.00 | 0.63 | 1.53 | 4.79 |
| | ACN | 392 | 5.5 | 9.65 | 0.38 | 0.17 | 9.81 | 4.53 | 2.16 | 0.43 | 1.12 | 10.38 |
| **TPA-BBT** | Hexane | 401 | 7.8 | 10.10 | 0.77 | 0.47 | 10.03 | 6.11 | 0.69 | 0.25 | 0.33 | 3.68 |
| | THF | 410 | 7.5 | 10.23 | 0.97 | 0.52 | 9.88 | 5.35 | 0.50 | 0.13 | 0.14 | 3.61 |
| | ACN | 402 | 7.5 | 10.40 | 1.49 | 0.48 | 8.87 | 3.26 | 0.35 | 0.15 | 0.10 | 2.43 |
| **TPA-S** | Hexane | 400 | 4.2 | 8.09 | 0.04 | 0.01 | 8.14 | 3.54 | 27.42 | 0.95 | 26.35 | 10.70 |
| | THF | 406 | 4.2 | 8.17 | 0.15 | 0.05 | 8.04 | 3.26 | 6.30 | 0.92 | 6.07 | 2.26 |
| | ACN | 402 | 4.2 | 8.16 | 0.28 | 0.09 | 8.68 | 3.17 | 3.23 | 0.90 | 3.21 | 0.20 |
| **TPA-BBS** | Hexane | 408 | 7.5 | 10.15 | 0.10 | 0.06 | 10.16 | 5.96 | 9.53 | 0.93 | 9.40 | 1.27 |
| | THF | 414 | 7 | 10.11 | 0.16 | 0.08 | 9.73 | 4.86 | 5.94 | 0.91 | 5.83 | 1.16 |
| | ACN | 407 | 7.5 | 10.15 | 0.22 | 0.08 | 10.95 | 3.54 | 4.23 | 0.81 | 3.73 | 5.06 |
| **TPA-EDOT** | Hexane | 397 | 4.8 | 8.49 | 0.51 | 0.21 | 8.49 | 4.17 | 1.53 | 0.70 | 1.36 | 1.68 |
| | THF | 403 | 4.7 | 8.52 | 0.87 | 0.35 | 8.51 | 4.06 | 0.75 | 0.41 | 0.47 | 2.80 |
| | ACN | 398 | 4.6 | 8.46 | 1.17 | 0.39 | 8.27 | 3.32 | 0.52 | 0.31 | 0.27 | 2.58 |

# G) References


(1) Andersen, M. W.; Hildebrandt, B.; Köster, G.; Hoffmann, R. W. Stereoselective Synthesis of Alcohols, XXX: E- and Z-Pentenylboronates, Reagents for Simple Diastereoselection on Addition to Aldehydes. *Chem. Ber.* **1989**, *122* (9), 1777–1782. https://doi.org/10.1002/cber.19891220925.

(2) Navarro, O.; Nolan, S. P. Large-Scale One-Pot Synthesis of N-Heterocyclic Carbene-Pd(Allyl)Cl Complexes. *Synthesis* **2006**, 366–367. https://doi.org/10.1055/s-2005-918497.

(3) Yasuda, T.; Shimizu, T.; Liu, F.; Ungar, G.; Kato, T. Electro-Functional Octupolar π-Conjugated Columnar Liquid Crystals. *Journal of the American Chemical Society* **2011**, *133* (34), 13437–13444. https://doi.org/10.1021/ja2035255.

(4) Søndergaard, R.; Manceau, M.; Jørgensen, M.; Krebs, F. C. New Low-Bandgap Materials with Good Stabilities and Efficiencies Comparable to P3HT in R2R-Coated Solar Cells. *Advanced Energy Materials* **2012**, *2* (4), 415–418. https://doi.org/10.1002/aenm.201100517.

(5) Leenen, M. A. M.; Vian, F.; Cucinotta, F.; Pisula, W.; Thiem, H.; Anselmann, R.; De Cola, L. Synthesis, Characterization and Field-Effect Transistor Performance of Poly[2,6-Bis(3-Alkylthiophen-2-Yl)Benzo[1,2-b;4,5-B′]Diselenophene]s. *Macromolecular Chemistry and Physics* **2010**, *211* (21), 2286–2291. https://doi.org/10.1002/macp.201000447.

(6) Haid, S.; Mishra, A.; Weil, M.; Uhrich, C.; Pfeiffer, M.; Bäuerle, P. Synthesis and Structure-Property Correlations of Dicyanovinyl-Substituted Oligoselenophenes and Their Application in Organic Solar Cells. *Advanced Functional Materials* **2012**, *22* (20), 4322–4333. https://doi.org/10.1002/adfm.201201018.

(7) Roncali, J.; Giffard, M.; Frere, P.; Jubault, M.; Gorgues, A. Extensively Conjugated Tetrathiafulvalene (TTF) π-Electron Donors with Oligothiophenes Spacer Groups. *Journal of the Chemical Society, Chemical Communications* **1993**, No. 8, 689. https://doi.org/10.1039/c39930000689.

(8) Wang, N.-X. Synthesis of 2-Bromo-2′-Phenyl-5,5′-Thiophene: Suzuki Reaction Versus Negishi Reaction. *Synthetic Communications* **2003**, *33* (12), 2119–2124. https://doi.org/10.1081/SCC-120021039.

(9) Odom, S. A.; Lancaster, K.; Beverina, L.; Lefler, K. M.; Thompson, N. J.; Coropceanu, V.; Brédas, J.-L.; Marder, S. R.; Barlow, S. Bis[Bis-(4-Alkoxyphenyl)Amino] Derivatives of Dithienylethene, Bithiophene, Dithienothiophene and Dithienopyrrole: Palladium-Catalysed Synthesis and Highly Delocalised Radical Cations. *Chemistry - A European Journal* **2007**, *13* (34), 9637–9646. https://doi.org/10.1002/chem.200700668.

(10) Doszczak, L.; Kraft, P.; Weber, H.-P.; Bertermann, R.; Triller, A.; Hatt, H.; Tacke, R. Prediction of Perception: Probing the HOR17-4 Olfactory Receptor Model with Silicon Analogues of Bourgeonal and Lilial. *Angewandte Chemie International Edition* **2007**, *46* (18), 3367–3371. https://doi.org/10.1002/anie.200605002.

(11) Goodbrand, H. B.; Hu, N.-X. Ligand-Accelerated Catalysis of the Ullmann Condensation: Application to Hole Conducting Triarylamines. *The Journal of Organic Chemistry* **1999**, *64* (2), 670–674. https://doi.org/10.1021/jo981804o.

(12) Aizawa, N.; Pu, Y.-J.; Sasabe, H.; Kido, J. Solution-Processable Carbazole-Based Host Materials for Phosphorescent Organic Light-Emitting Devices. *Organic Electronics* **2012**, *13* (11), 2235–2242. https://doi.org/10.1016/j.orgel.2012.06.036.

(13) Kautny, P.; Lumpi, D.; Wang, Y.; Tissot, A.; Bintinger, J.; Horkel, E.; Stöger, B.; Hametner, C.; Hagemann, H.; Ma, D.; Fröhlich, J. Oxadiazole Based Bipolar Host Materials Employing Planarized Triarylamine Donors for RGB PHOLEDs with Low Efficiency Roll-Off. *Journal of Materials Chemistry C* **2014**, *2* (11), 2069. https://doi.org/10.1039/c3tc32338b.

(14) Anémian, R.; Cupertino, D. C.; Mackie, P. R.; Yeates, S. G. Solution Phase Studies towards the Synthesis of Triarylamine Oligomers Using a Germanium Linker on a Solid Support. *Tetrahedron Letters* **2005**, *46* (39), 6717–6721. https://doi.org/10.1016/j.tetlet.2005.07.150.

(15) Marion, N.; Navarro, O.; Mei, J.; Stevens, E. D.; Scott, N. M.; Nolan, S. P. Modified (NHC)Pd(Allyl)Cl (NHC = *N* - Heterocyclic Carbene) Complexes for Room-Temperature Suzuki−Miyaura and Buchwald−Hartwig Reactions. *Journal of the American Chemical Society* **2006**, *128* (12), 4101–4111. https://doi.org/10.1021/ja057704z.

(16) Liu, C.-Y.; Zhao, H.; Yu, H. Efficient Synthesis of 3,4-Ethylenedioxythiophene (EDOT)-Based Functional π-Conjugated Molecules through Direct C–H Bond Arylations. *Organic Letters* **2011**, *13* (15), 4068–4071. https://doi.org/10.1021/ol201571u.

(17) Yanai, T.; Tew, D. P.; Handy, N. C. A New Hybrid Exchange–Correlation Functional Using the Coulomb-Attenuating Method (CAM-B3LYP). *Chem. Phys. Lett.* **2004**, *393* (1–3), 51–57. https://doi.org/10.1016/j.cplett.2004.06.011.

(18) Frisch, M. J.; Trucks, G. W.; Schlegel, H. B.; Scuseria, G. E.; Robb, M. A.; Cheeseman, J. R.; Scalmani, G.; Barone, V.; Mennucci, B.; Petersson, G. A.; Nakatsuji, H.; Caricato, M.; Li, X.; Hratchian, H. P.; Izmaylov, A. F.; Bloino, J.; Zheng, G.; Sonnenberg, J. L.; Hada, M.; Ehara, M.; Toyota, K.; Fukuda, R.; Hasegawa, J.; Ishida, M.; Nakajima, T.; Honda, Y.; Kitao, O.; Nakai, H.; Vreven, T.; Montgomery Jr., J. A.; Peralta, J. E.; Ogliaro, F.; Bearpark, M. J.; Heyd, J.; Brothers, E. N.; Kudin, K. N.; Staroverov, V. N.; Kobayashi, R.; Normand, J.; Raghavachari, K.; Rendell, A. P.; Burant, J. C.; Iyengar, S. S.; Tomasi, J.; Cossi, M.; Rega, N.; Millam, N. J.; Klene, M.; Knox, J. E.; Cross, J. B.; Bakken, V.; Adamo, C.; Jaramillo, J.; Gomperts, R.; Stratmann, R. E.; Yazyev, O.; Austin, A. J.; Cammi, R.; Pomelli, C.; Ochterski, J. W.; Martin, R. L.; Morokuma, K.; Zakrzewski, V. G.; Voth, G. A.; Salvador, P.; Dannenberg, J. J.; Dapprich, S.; Daniels, A. D.; Farkas, Ö.; Foresman, J. B.; Ortiz, J. V.; Cioslowski, J.; Fox, D. J. *Gaussian 09*; Gaussian, Inc.: Wallingford, CT, USA, 2009.

(19) Grimme, S.; Antony, J.; Ehrlich, S.; Krieg, H. A Consistent and Accurate *Ab Initio* Parametrization of Density Functional Dispersion Correction (DFT-D) for the 94 Elements H-Pu. *J. Chem. Phys.* **2010**, *132* (15), 154104. https://doi.org/10.1063/1.3382344.

(20) Ferrighi, L.; Frediani, L.; Fossgaard, E.; Ruud, K. Two-Photon Absorption of [2.2]Paracyclophane Derivatives in Solution: A Theoretical Investigation. *J. Chem. Phys.* **2007**, *127* (24), 244103. https://doi.org/10.1063/1.2814168.

(21) McClain, W. M. Excited State Symmetry Assignment Through Polarized Two-Photon Absorption Studies of Fluids. *J. Chem. Phys.* **1971**, *55* (6), 2789–2796. https://doi.org/10.1063/1.1676494.

(22) Friese, D. H.; Beerepoot, M. T. P.; Ringholm, M.; Ruud, K. Open-Ended Recursive Approach for the Calculation of Multiphoton Absorption Matrix Elements. *J. Chem. Theory Comput.* **2015**, *11* (3), 1129–1144. https://doi.org/10.1021/ct501113y.

(23) Ågren, H.; Vahtras, O.; Koch, H.; Jørgensen, P.; Helgaker, T. Direct Atomic Orbital Based Self-consistent-field Calculations of Nonlinear Molecular Properties. Application to the Frequency Dependent Hyperpolarizability of *Para* -nitroaniline. *J. Chem. Phys.* **1993**, *98* (8), 6417–6423. https://doi.org/10.1063/1.465099.

(24) Aidas, K.; Angeli, C.; Bak, K. L.; Bakken, V.; Bast, R.; Boman, L.; Christiansen, O.; Cimiraglia, R.; Coriani, S.; Dahle, P.; Dalskov, E. K.; Ekström, U.; Enevoldsen, T.; Eriksen, J. J.; Ettenhuber, P.; Fernández, B.; Ferrighi, L.; Fliegl, H.; Frediani, L.; Hald, K.; Halkier, A.; Hättig, C.; Heiberg, H.; Helgaker, T.; Hennum, A. C.; Hettema, H.; Hjertenaes, E.; Høst, S.; Høyvik, I.-M.; Iozzi, M. F.; Jansík, B.; Jensen, H. J. Aa.; Jonsson, D.; Jørgensen, P.; Kauczor, J.; Kirpekar, S.; Kjaergaard, T.; Klopper, W.; Knecht, S.; Kobayashi, R.; Koch, H.; Kongsted, J.; Krapp, A.; Kristensen, K.; Ligabue, A.; Lutnaes, O. B.; Melo, J. I.; Mikkelsen, K. V.; Myhre, R. H.; Neiss, C.; Nielsen, C. B.; Norman, P.; Olsen, J.; Olsen, J. M. H.; Osted, A.; Packer, M. J.; Pawlowski, F.; Pedersen, T. B.; Provasi, P. F.; Reine, S.; Rinkevicius, Z.; Ruden, T. A.; Ruud, K.; Rybkin, V. V.; Sałek, P.; Samson, C. C. M.; de Merás, A. S.; Saue, T.; Sauer, S. P. A.; Schimmelpfennig, B.; Sneskov, K.; Steindal, A. H.; Sylvester-Hvid, K. O.; Taylor, P. R.; Teale, A. M.; Tellgren, E. I.; Tew, D. P.; Thorvaldsen, A. J.; Thøgersen, L.; Vahtras, O.; Watson, M. A.; Wilson, D. J. D.; Ziolkowski, M.; Ågren, H. The Dalton Quantum Chemistry Program System: The Dalton Program. *Wiley Interdisciplinary Reviews: Computational Molecular Science* **2014**, *4* (3), 269–284. https://doi.org/10.1002/wcms.1172.

(25) Beerepoot, M. T. P.; Friese, D. H.; List, N. H.; Kongsted, J.; Ruud, K. Benchmarking Two-Photon Absorption Cross Sections: Performance of CC2 and CAM-B3LYP. *Phys. Chem. Chem. Phys.* **2015**, *17* (29), 19306–19314. https://doi.org/10.1039/C5CP03241E.

(26) Frediani, L.; Rinkevicius, Z.; Ågren, H. Two-Photon Absorption in Solution by Means of Time-Dependent Density-Functional Theory and the Polarizable Continuum Model. *10.1063/1.1944727* **2005**, *122* (24), 244104. https://doi.org/10.1063/1.1944727.

(27) Friese, D. H.; Mikhaylov, A.; Krzeszewski, M.; Poronik, Y. M.; Rebane, A.; Ruud, K.; Gryko, D. T. Pyrrolo[3,2-b]Pyrroles-From Unprecedented Solvatofluorochromism to Two-Photon Absorption. *Chemistry - A European Journal* **2015**, *21* (50), 18364–18374. https://doi.org/10.1002/chem.201502762.

(28) Fukuda, R.; Ehara, M. Excited States and Electronic Spectra of Annulated Dinuclear Free-Base Phthalocyanines: A Theoretical Study on near-Infrared-Absorbing Dyes. *J. Chem. Phys.* **2012**, *136* (11), 114304. https://doi.org/10.1063/1.3692964.

(29) List, N. H.; Olsen, J. M.; Rocha-Rinza, T.; Christiansen, O.; Kongsted, J. Performance of Popular XC-Functionals for the Description of Excitation Energies in GFP-like Chromophore Models. *Int. J. Quantum Chem.* **2012**, *112* (3), 789–800. https://doi.org/10.1002/qua.23059.

(30) Kiefer, P.; Hahn, V.; Nardi, M.; Yang, L.; Blasco, E.; Barner-Kowollik, C.; Wegener, M. Sensitive Photoresists for Rapid Multiphoton 3D Laser Micro- and Nanoprinting. *Adv. Optical Mater.* **2020**, *8* (19), 2000895. https://doi.org/10.1002/adom.202000895.

(31) Kolb, C.; Lindemann, N.; Wolter, H.; Sextl, G. 3D-printing of Highly Translucent ORMOCER®-based Resin Using Light Absorber for High Dimensional Accuracy. *J. Appl. Polym. Sci.* **2021**, *138* (3), 49691. https://doi.org/10.1002/app.49691.
